# Biodiversity in Urban Areas: The Extraordinary Case of Appia Antica Regional Park (Rome, Italy)

**DOI:** 10.3390/plants11162122

**Published:** 2022-08-15

**Authors:** Duilio Iamonico

**Affiliations:** Ce.R.S.I.Te.S., University of Rome Sapienza, Viale XXIV Maggio 7, 04100 Latina, Italy; d.iamonico@yahoo.it

**Keywords:** alien status, biological records, Europe, Latium, Mediterranean flora, Rome, urban biodiversity

## Abstract

The first inventory of the flora of Appia Antica Regional Park (Italy), one of the largest protected urban areas in Europe (4580 ha), its biological, ecological and biogeographical composition, and notes of the vegetation physiognomies and landscape are presented; physical characteristics of the territory (geomorphology, lithotypes, and phytoclimate) are also given. The landscape is defined by an agricultural matrix with natural and seminatural areas as patches, and riparian vegetation communities as corridors. The vegetation physiognomies are represented by types linked to the Mediterranean climate (mixed, Mediterranean, and riparian forests; scrubby, rocky, aquatic, and helophytic vegetation; anthropogenic communities). The floristic list includes 714 taxa (104 families and 403 genera). Therophytes prevail over hemicryptophytes; woody flora comprises about 30% of alien species. As regards chorotypes, together with a considerable number of Mediterranean species, there are many exotic species with wide distribution areas testifying to a long-lasting anthropic impact. Floristic novelties (european, national, and regional levels) for 21 taxa are reported. The extraordinary species diversity discovered (43% of flora of Rome and 20% of regional flora) is linked to the landscape heterogeneity, the characteristics of which are: (1) persistence of residual natural patches, (2) occurrence of quite well-preserved aquatic habitats and humid meadows, (3) a rich anthropogenic flora, (4) an interesting flora of archeological sites, (5) occurrence of species not common in Latium, (6) occurrence of populations of aliens in crops (which cause economic impact), (7) presence of aliens on archeological ruins (which cause economic-social impacts). The extensive set of data provided represents a general base framework for guiding future research efforts and landscape action plans consistent with environmental sustainability.

## 1. Introduction

Biodiversity loss is a phenomenon mainly related to the intensification of land use and management, as well as the conversion of natural lands to agricultural, forestry, and building areas [1]. This phenomenon is particularly intense in urban areas where several processes, e.g., loss of soil, biological invasions or pollution, are highly exacerbated due to human activities and have a significant impact on the native flora and residual fragments of natural vegetation [2,3,4]. In addition, as widely known, urbanization has been accelerating for several decades at an alarming rate around the world, and most of the world’s population is now concentrated in urban areas [5]. Urbanization is one of the major causes of plant diversity loss at the local and regional scales [6].

Urban areas are “hotspots” of biological invasions, being not only key points of entry for many non-native species, but also playing an important role in the secondary spread of aliens towards surrounding territories, i.e., rural or natural landscapes [3,7]. However, at the same time, urban parks and natural areas in cities are important as detailed-scale biodiversity hotspots [8]. So, maintenance of the biodiversity in urban areas represents a very important conservation issue [9,10]. With this aim in mind, the first fundamental step for sustainable actions is to improve the floristic knowledge of these areas by preparing inventories of taxa [9,11]. Floristic catalogues provide useful data for subsequent studies, e.g., distributional patterns [12], or for future actions aimed, for instance, at managing non-native taxa [13].

The flora of Mediterranean cities still remains poorly known [9]. Concerning Italy, most urban floras refer to cities of northern Italy, e.g., Milan in Lombardy region [14] or Trieste in Friuli-Venezia Giulia region [15]. Concerning central and southern Italy, few contributions have been published, e.g., for Naples in Campania region [16] or Rome in Lazio region [9].

Rome, and its administrative territory, is one of the largest and most populated urban areas in southern Europe, and it has been investigated since the 19th century from a botanical point of view. The first published floristic works of the Roman area were by Sebastiani in 1813 and 1815 [17,18] and Sebastiani and Mauri in 1818 [19], whereas the more recent list of spontaneous plants growing in Rome was published about 10 years ago [9]. The territory of Rome Municipality covers 1287 km^2^, and it is characterized by very high landscape heterogeneity dependent primarily on its location in the center of the Mediterranean basin. This location favors, in turn, the influence by various environmental factors, e.g., biogeographic effects (from western and eastern Europe), proximity of the Tyrrhenian Sea (on the west) and Apennine Mountains (on the east), different types of phytoclimates, 3000 years of human impact, etc. [9,20]. These environmental features contribute to a high floristic richness (1649 taxa according to [9]) as well as the occurrence of many types of natural vegetation communities and potential types, the dominant ones being the sub-Mediterranean deciduous oak woods with *Quercus cerris* L., *Q. frainetto* Ten., *Q. robur* L., and *Q. pubescens* Willd. Despite the quite extensive knowledge of flora of the Roman area, detailed studies on specific areas (e.g., those protected by regional or national laws) are still partially lacking. Some of these areas have been studied in recent years, e.g., the Monte Mario Natural Reserve in the northwestern part of the Rome Municipality [21], the Laurentino-Acqua Acetosa Natural Reserve in the southwestern part [22], or the Augazzano Urban Park [23] and Nomentum Natural Reserve [24] in the eastern part.

Appia Antica Regional Park, which is the topic of the present work, was not investigated in detail from a floristic point of view, despite being one of the larger protected areas of the Rome Municipality and, as an urban park, in the whole of Europe (with about 4580 ha). There is only a floristic catalogue on the Caffarella Valley that occupies only 190 ha (about 4% of the total area), but it was published 22 years ago [25].

As a part of ongoing studies on the flora of urban areas, with special attention to the Rome territory [21,22,23,26], I here present the flora of Appia Antica Regional Park, also giving notes on abiotic factors (climate, geology, geomorphology, etc.) as well as landscape structure and main vegetation physiognomies.

## 2. Materials and Methods

This work was based mainly on field surveys that were carried out during the period 2010–2022. Collected material was deposited mainly at the Herbarium RO (secondly at FI and HFLA). Further specimens, kept at A, AC, BM, BR, CAS, COL, F, FI, G, GH, GOET, HFLA, JE, K, MICH, MO, NA, P, RO, RSA, SI, UC, US, URT, and YU, were examined (acronyms of the herbaria follow *Index Herbariorum* [27]). Relevant botanical literature was also analyzed (citations occur throughout the text).

The taxa (species, subspecies, and variety ranks) were identified using the new edition of the *Flora of Italy* [28]; recent monographs were also considered for critical genera (e.g., *Amaranthus* [29] or *Vitis* [30]). The nomenclature follows the Italian Checklists of both native and non-native flora and the subsequent updates [31,32,33]. Concerning the genera *Amaranthus* and *Cyanus*, I considered, respectively, my recent monograph [29] and the new edition of the *Flora of Italy* [28]. Biological forms and chorotypes of each taxon follow the new edition of the *Flora of Italy* [28]; grouped chorotypes follow [26], except for the category “Aliens”, which includes, in the present paper, all the non-native taxa. The alien status and definitions of categories follow the recent Italian Checklist of non-native flora [31] and Pyšek and collaborators [34].

In the floristic list (Appendix A), the systematic order of the families follows Italian Checklists of both native and non-native flora [31,32]. Within each family, the taxa are ordered alphabetically. For each taxon, after the accepted scientific name, the following information is reported: endemic, cryptogenic, alien status.

Geological and geomorphological information and the pertinent nomenclature refer mainly to [35,36] and [37,38], respectively. Further, field observations were conducted.

Climatic and phytoclimatic data derive from the climate classification by Zepner and collaborators [39] and the map of the Roman area by Blasi and Michetti [40].

Landscape remarks are based on [41,42], but also on direct observations in the field.

Vegetation physiognomies were observed directly in the field, lacking a detailed phytosociological study of the park area (see also [41]). Nomenclature of the main syntaxonomic ranks follows the recent classification system for European vegetation [43].

Photographs are original and produced by the author of the present manuscript, except for the bottom picture of Figure 8 (“Cava di Fioranello”), which was produced using a drone and kindly provided by Lucio Virzì (Rome).

## 3. Study Area

### 3.1. Geographical Context

The Appia Antica Regional Park is located mostly in the southeastern part of the urban area of Rome Municipality (less than 5% of the territory is included in the Municipalities of Ciampino and Marino, toward the south) and was established in 1988 by the Regional Law No. 66. Later, in 2002, by the Regional Law 31 May 2002, an extension of the original area was proposed and approved. Currently, the park covers about 4580 ha; altitude ranges from about 15 m in the north sector of the park to 189 m a.s.l. in the south (Frattocchie locality, Marino Municipality).

**Figure 1:**
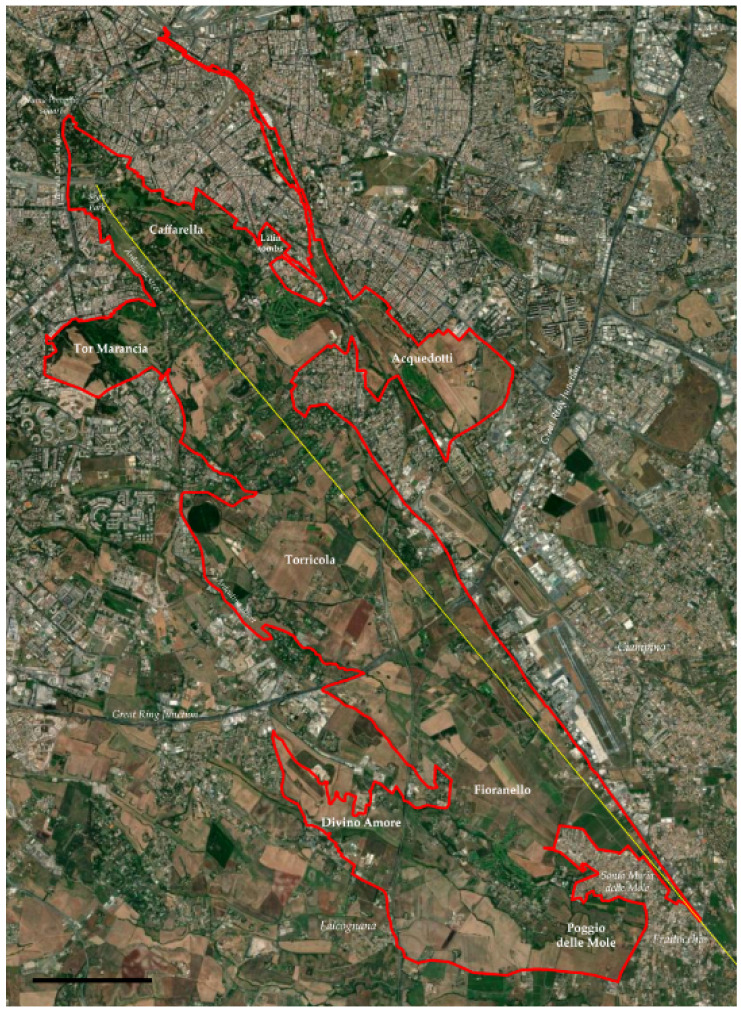
Map of Appia Antica Regional Park (boundary in red line) with main localities and streets; yellow line: Appia Antica street. Scale bar = 2 km.

The general shape of the park is a long wedge defined by one main axis, the Appia Antica street, which runs for about 16 km from northeast (Numa Pompilio square, in the vicinity of the archeological area of *Caracalla* thermal baths, in the historic center of Rome) to southwest (Ciampino and Marino Municipalities). Coordinates of the park are: 41°50′00″ latitude N, 12°33′00″ longitude E. The main boundaries of the park are (see Figure 1):

⮚ on the west: Terme di Caracalla street up to the Scott Park in *Ardeatino* district (toward the south); Ardeatina street up to the hamlet *Falcognana* (part of the Municipality of Rome located about 4 km from the Great Ring Junction toward the south) with the exclusion of an area around Fioranello street. In addition, the area named *Tor Marancia* (about 220 ha) occurs to the west of Ardeatina street (*Ardeatino* district, north of the park);
⮚ on the south: Falcognana street between the hamlets *Falcognana* (to the west) and *Poggio Le Mole* (Municipality of Marino, to the east), with the exclusion of the hamlet *Santa Maria delle Mole*;
⮚ on the east: Appia Nuova street from the hamlet *Frattocchie* (Municipality of Marino) to the Roman urban area named *Arco di Travertino*; Latina street (with the exclusion of part of the district *Appio Latino* between Antonio Coppi street and the railway) up to Numa Pompilio square. In addition, two archeological areas occur to the east of Appia Nuova street, named *Acquedotti* (about 240 ha) and *Latin tombs* (about 2 ha);
⮚ on the north: Numa Pompilio square.

### 3.2. Geological Features

**Figure 2:**
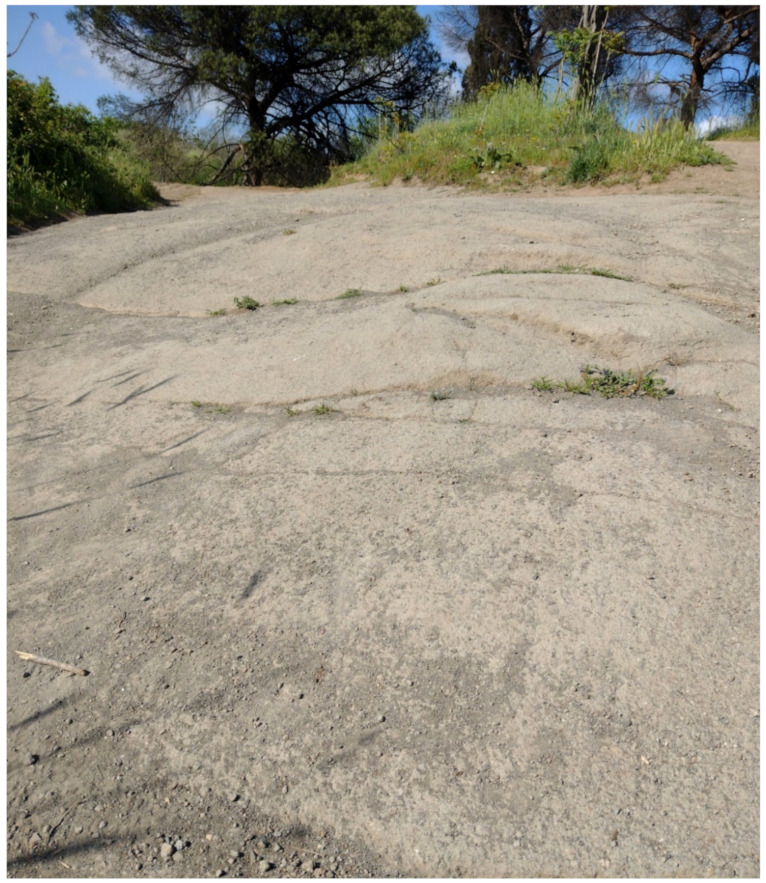
Lava *Colata di Capo di Bove* (south of Caffarella valley, north of the park).

**Figure 3:**
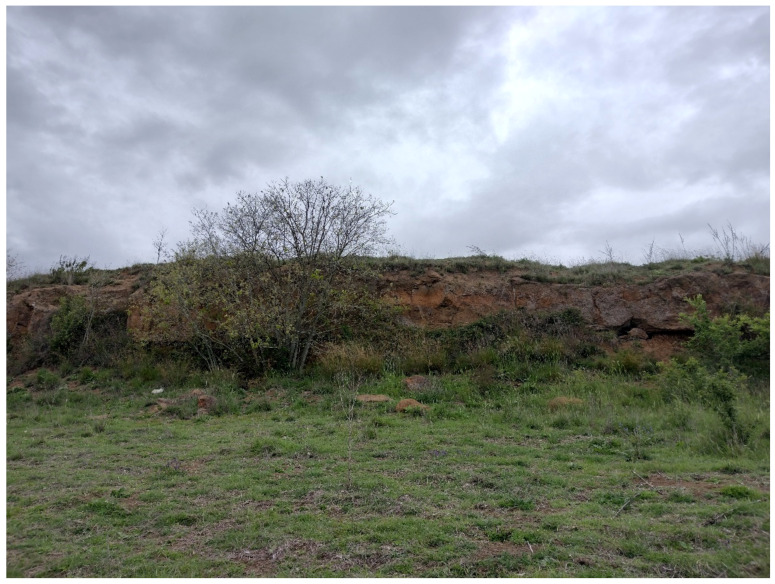
Unconsolidated pyroclastic deposits (*Pozzolane*) in the northwestern sector of Caffarella valley (north of the park).

The area of Appia Antica Regional Park had a geological history strictly linked with the activity of the Colli Albani volcano (currently known as *Castelli Romani*), which started about 600,000 years ago [26]. During the first phase (0.60–0.36 Ma), violent eruptions occurred, causing the formation of a large volcanic structure. This later collapsed, giving rise to the Tuscolano-Artemisian caldera. A second volcanic phase (0.27–0.10 Ma) was marked by the rise of a second volcanic structure, internal to the previous one. Finally, there was a third phase (0.10–0.01 Ma), which was characterized by intense hydromagmatic activity, creating the lakes Albano and Nemi plus several minor basins, drained in the past by man for agricultural purposes (Vallericcia, Laghetto, Valle Marciana, Prata Porci, Pantano Secco). The territory of Appia Antica Regional Park, which is located from the north-western slope of *Castelli Romani* to the historic center of Rome (toward the north), is covered by lithotypes related to volcanic rocks with alkaline-potassic chemism [26,35]. Three main types occur: lavas, tuffs, and unconsolidated pyroclastic deposits (named also as *Pozzolane*). The former is related to the volcanic activity of the above-mentioned second phase, when just one erupted material flowed out of the Tuscolano-Artemisian caldera toward the current urban area of Rome. This lava was named *Colata di Capo di Bove* (literally “Lava flow of Bove Capo”) and it appears in various parts of the park (Figure 2). The remaining area is covered by various types of tuffs and unconsolidated pyroclastic deposits (Figure 3). In addition to lavas, tuffs, and *Pozzolane*, lahar deposits occur in the *Acquedotti* area (east of the park) being part of the Ciampino Plain, the origin of which represents the most recent activity of Colli Albani volcano (25 Ka; [36]).

Holocene deposits are represented by alluvials and colluvials of sands, silty sand, or silty clayey sand related to the fluvial processes [35].

### 3.3. Geomorphological Features

As a whole, the natural landscape in which the city of Rome has developed was mainly molded by fluvial processes. In addition, polygenetic, structural, and gravitational forms also occurred and are widespread; finally, there are many landforms deriving from the millennian human activities [38].

The study area includes all four main types of landforms that can be found in the whole Roman area [38], i.e.,:

⮚ Fluvial landforms: the study area is characterized by a more or less structured hydrographic network composed mainly of channels, mostly with steady water flows, natural ponds, and freshwater springs. The most complex networks can be found in the *Caffarella* (north of the park) and *Acquedotti* (east) areas.

**Figure 4:**
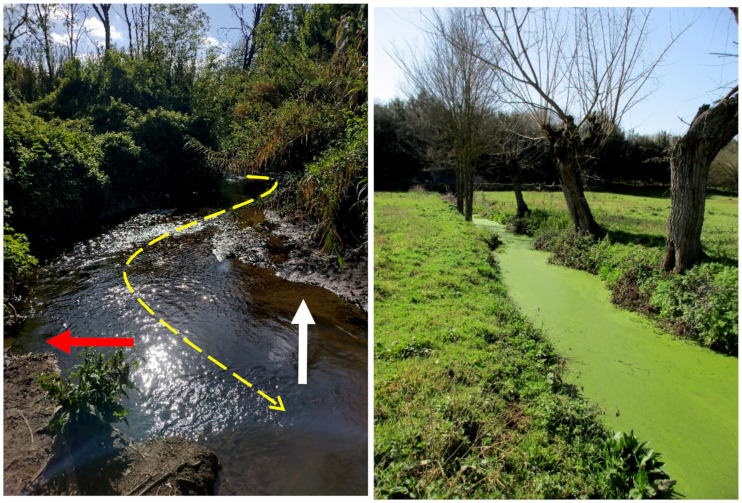
Meander of river Almone (yellow line: flow direction): deposition of material on the inside bank of the bend (white arrow) and erosion of the outside bank (red arrow) (left side photo); secondary channel in Caffarella valley (right side photo).

The *Caffarella* area is a typical V-shaped valley molded by the river Almone and its tributaries (channels, locally named *Marrane*). Almone is a left-side tributary of the river Tevere originated in the Colli Albani volcano and joining the river Tevere in the *Ostiense* district (southwestern Rome); the total length of Almone is about 21 km, and its drainage basin is about 51 km^2^ [44]. In particular, the river Almone, which has a mean river flow of 1.7 m^2^/s, forms on some parts of its alluvial plain a series of meanders with deposition of material on the inside of each bend and erosion of the outside bank of the bend (Figure 4).

The *Acquedotti* area is flat with a main channel (named *Acqua Mariana*, that originates in Molara Valley in the Castelli Romani Regional Park [45] about 20 km toward the south) and secondary channels (Figure 4). *Acqua Mariana* is an artificial channel created during the 12th century by redirecting a tributary of the natural channel *Acqua Crabra* [36,44]. The hydrographic network was affected by erosion of lahar deposits distributed in the Ciampino plain (<23 Ma) [46].

**Figure 5:**
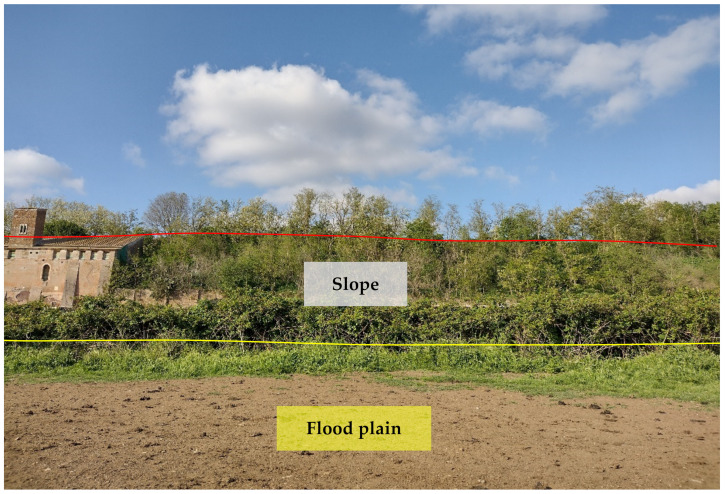
Flat surface on east side of the Caffarella valley. Flood plain is at about 20 m a.s.l., top of the structural landform (red line) is at about 43 m a.s.l. Yellow line: base of the slope (45–80°).

⮚ Structural landforms: these forms are the results of the fluvial erosion cut of the flat ignimbritic plateau generated after the eruption of the Colli Albani volcano (middle Pleistocene); surfaces are often bordered by cliffs affected by various weathering and denudation processes [37]. Some structural landforms can be observed in the Caffarella valley, where the volcanic plateau was cut by the Almone River (Figure 5).

**Figure 6:**
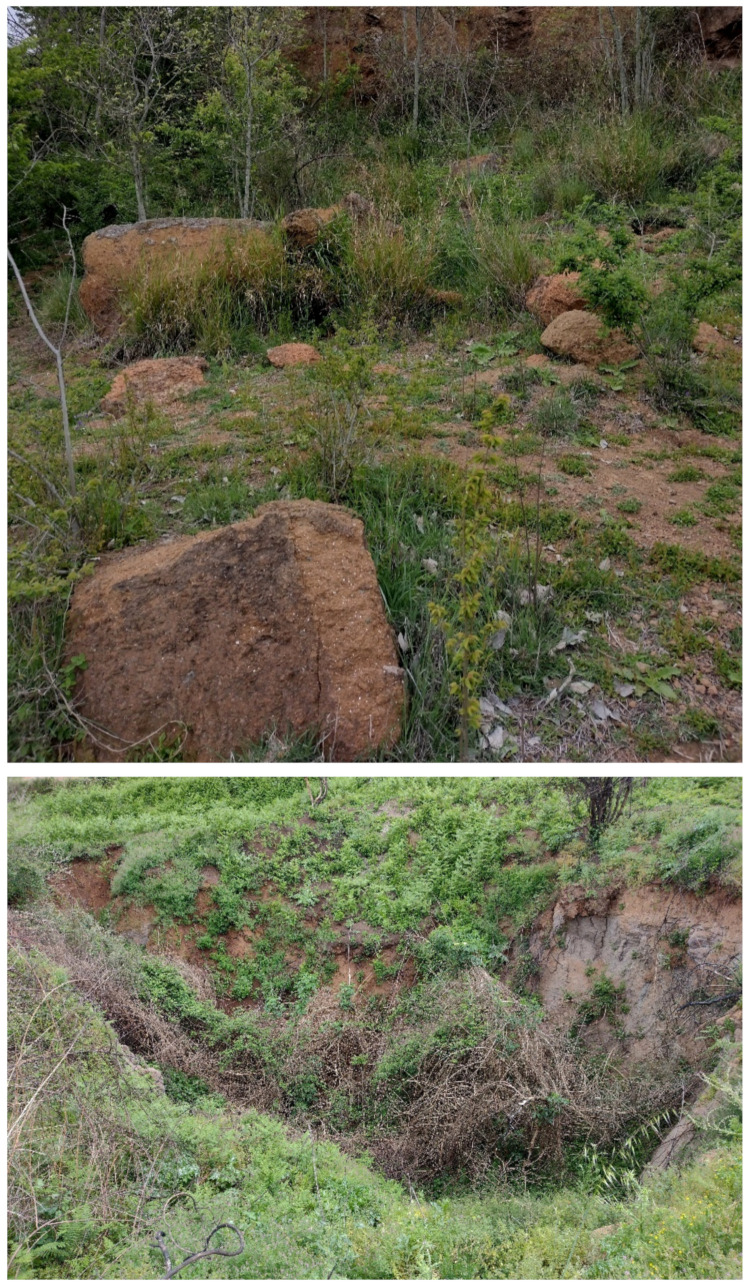
Gravitational landforms: fall on the east side of Caffarella valley (top photo); sinkhole, diameter about 12 m, depth about 5 m (bottom photo).

⮚ Gravitational landforms: two main types were observed, i.e., falls, which occur especially in the Caffarella valley and Tor Marancia locality where there are some slopes with a high gradient (more than 80%, even vertical) (Figure 6) (no data occur in the map of the Italian landslide for the study area [47]) and sinkholes originating from the collapse of underground cavities (see below under “Man-made landforms”) and the subsequent subsidence of the shallower layers of the soil [48]. The latter gravitational landforms have dimensions ranging from 1–6 m of depth and 1–12 m of diameter (pers. obs.) (Figure 6).

⮚ Man-made landforms: the study area has been affected by several human activities mostly beginning thousand years ago. The marks of these activities are often juxtaposed with those related to the natural processes [37].

**Figure 7:**
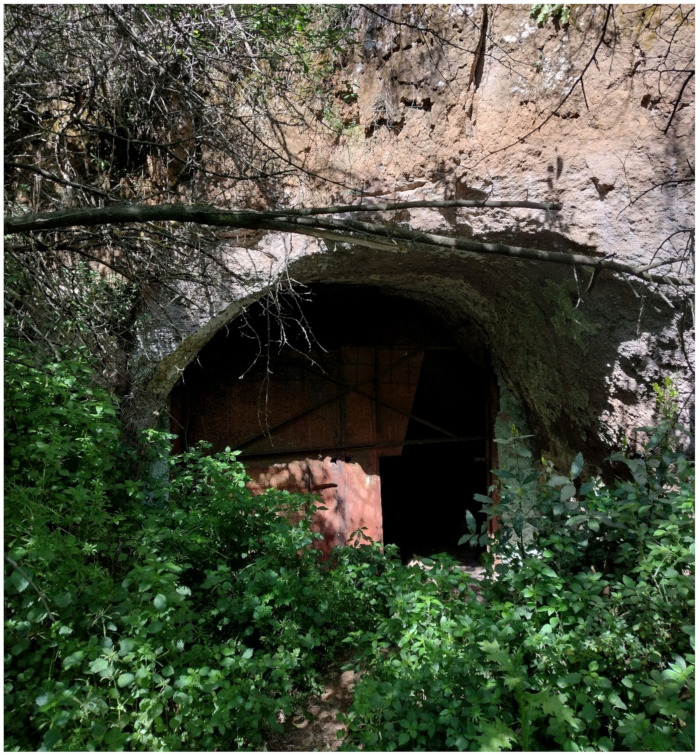
Entrance of an underground cavity (Caffarella valley).

The most widespread human-made landforms are represented by a dense network of underground cavities, which are especially concentrated in the Caffarella area (north of the park). These cavities (Figure 7) were firstly used to extract material for construction of buildings and catacombs, i.e., lithic tuffs and unconsolidated pyroclastic deposits (*Pozzolane*), and to distribute and collect water [48]. The more recent use of these cavities (up to the 1990s) was as mushroom patches (genera *Pleurotus* (Fr.) P. Kumm. and *Agaricus* L. (*A. bisporus* (J.E.Lange) Imbach, named “champignon”)).

**Figure 8:**
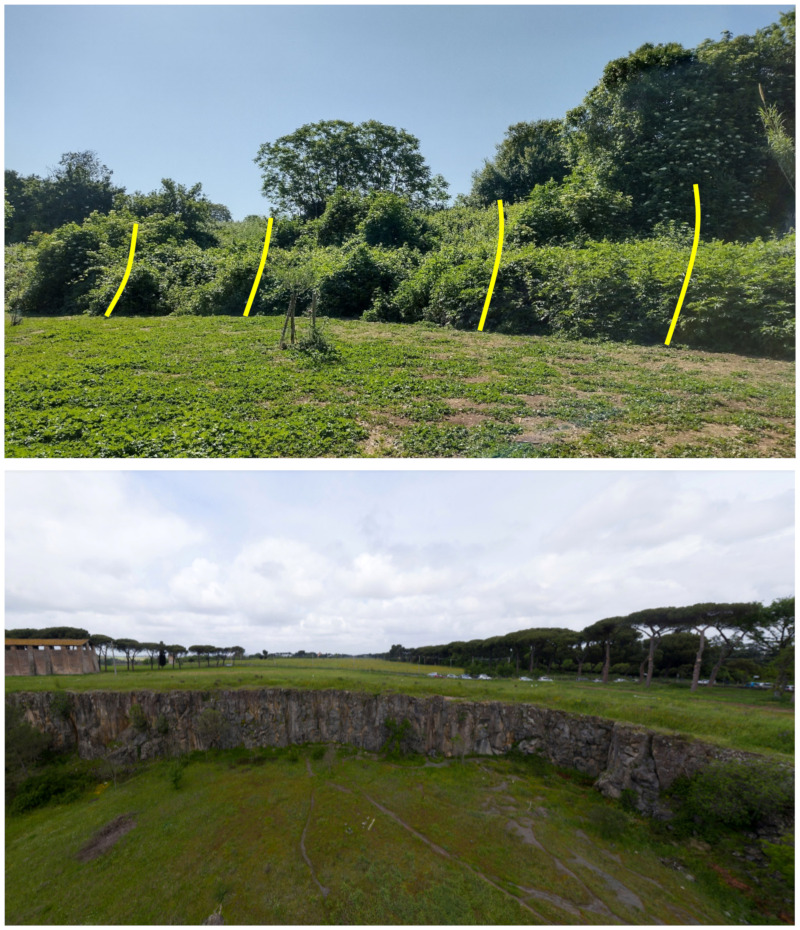
Mines: abandoned mine of Pozzolana located near the Latina street (northeast of the park); its slope (yellow lines) is covered by natural vegetation (top photo); mine *Cava di Fioranello* of basalt, near Ciampino airport, south of the park (bottom photo).

Further landforms occurring in Appia Antica Park are the mines (currently inactive). They started during the 6th–5th centuries BC [49] and are characterized by both straight scarps and step-like slopes. In some cases, the mines are no longer readily visible, being covered by the vegetation and/or subjected to naturalistic engineering operations ([50]; Figure 8). A famous mine (named *Cava di Fioranello*; Figure 8) occurs near the Ciampino airport (south of the park) and was used to extract basalt to prepare a type of cobblestone resembling truncated and square-based pyramids (locally named *sanpietrini*) that represents the traditional pavement of many streets and squares in the city of Rome [51]; Fioranello’s mine is currently used for climbing sport activity.

**Figure 9:**
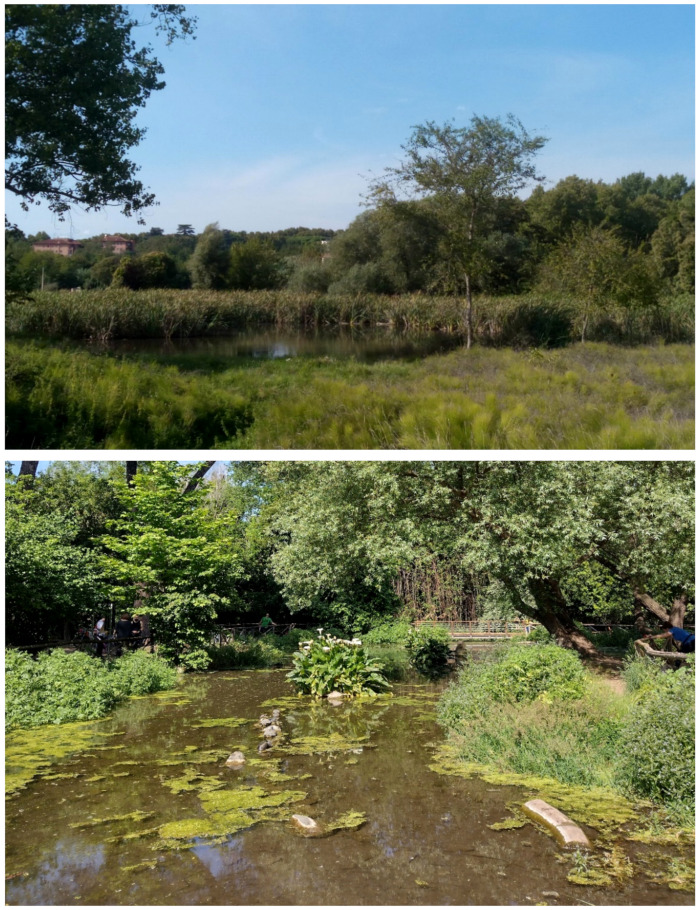
Artificial lakes occurring in Caffarella valley (top photo) and Acquedotti locality (bottom photo).

In addition to underground cavities and mines, there are further man-made elements that deserve to be mentioned since they locally changed the landscape from both environmental and social points of view. These man-made landforms are the artificial lakes occurring in Caffarella valley and Acquedotti locality (Figure 9):

⮚ the first lake (locally named *Laghetto della Caffarella* = Caffarella’s small lake) was created in 2004 for the natural regeneration of aquatic habitats. Caffarella’s lake has one tributary and one emissary (linked with the nearby channel), covers an area of about 2000 m^2^ (including the surrounding humid meadows), and has a maximum depth of 1.5 m;
⮚ the second lake (locally named *Laghetto degli Acquedotti* = Acquedotti’s small lake) was redeveloped about 10 years ago under planning for both the body of water and the adjacent channel *Acqua Mariana*, which serves as both tributary and emissary. The Aquedotti’s lake covers an area of about 800 m^2^ and has a maximum depth of 0.5 m.

### 3.4. Climate and Phytoclimate

According to the online ClimateCharts.net (accessed on 11 July 2022) database, which is based on the Köppen-Geiger’s climate classification [39], the study area would be included in the Temperate type (“C”), which is defined by a temperature of the hottest month of ≥10 °C and temperature of the coldest month ranging between 0 and 18 °C. In particular, the subtype is “Temperate without dry season” (code “Cfa”), defined by precipitation in the driest month in summer of >40 mm rain and temperature in the hottest month of ≥22 °C.

**Figure 10:**
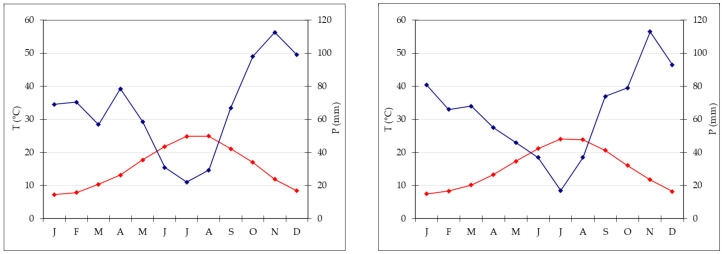
Thermo-Pluviometric Bagnolous-Gaussen diagrams of the stations Ciampino (left side diagram) and Monte Mario (right side diagram). Blue lines refer to rainfalls; red lines refer to temperatures. Axis x refers to months (e.g., “J” = January).

From the phytoclimatic point of view, the whole Roman area belongs to the Mediterranean region and the Meso Mediterranean type [40]. Appia Antica Regional Park is included in the following two subtypes:

⮚ Mesomediterranean Subhumid-Thermomediterranean Subhumid: this subtype (area outside of the Great Ring Junction) is characterized by a mean annual precipitation of 680–820 mm, mean summer precipitation of 82.23–96.34 mm, mean annual temperature of 14.60–15.21 °C, maximum annual temperature of 19.95–21.39 °C, minimum annual temperature of 9.27–10.41 °C. The potential natural vegetation concerns four vegetation series: Turkey oak Series [*Teucrio siculi-Quercion cerris* Ubaldi 1988 (*Crataego levigatae-Quercion cerridis* Arrigoni 1997 *sensu* [43])], Downy and Turkey oaks Series [*Ostryo-Carpinion orientalis* Horvat 1959 (*Fraxino orni-Ostryon* Tomažič 1940 *sensu* [43]) and *Lonicero etruscae-Quercion pubescentis* Arrigoni et Foggi ex Foggi et al. 1990 (*Crataego laevigatae-Quercio cerridis* Arrigoni 1997 *sensu* [43])], Holm oak Series (*Quercion ilicis* Br.-Bl. ex Molinier 1934), and Hornbeam Series [*Doronico-Fagion* Ubaldi et al. 1990 (*Geranio striati-Fagion* Gentile 1970 *sensu* [43])]. As a meteorological reference point, the thermo-pluviometric station is that named *Ciampino* (129 m a.s.l.); the thermo-pluviometric diagram (Figure 10) shows mean temperatures of the coldest (January) and the hottest (August) months of, respectively, 7.3 and 24.9 °C; mean annual rainfall is 792.8 mm [maximum monthly value in November (112.6 mm), minimum in July (22.1 mm)]; aridity is about 3 months;
⮚ Mesomediterranean Subhumid-Thermomediterranean Dry: this subtype (interior area to the Great Ring Junction) is characterized by a mean annual precipitation of 650–820 mm, mean summer precipitation of 56.9–76.6 mm, mean annual temperature of 14.60–15.21 °C, maximum annual temperature of 18.88–21.16 °C, minimum annual temperature of 9.27–10.41 °C. Potential natural vegetation regards three vegetation series: Turkey oak Series [*Teucrio siculi-Quercion cerris* (*Crataego laevigatae-Quercio cerridis sensu* [43])], Downy and Turkey oak Series [*Ostryio-Carpinion orientalis* (*Fraxino orni-Ostryon sensu* [43]) and *Lonicero-Quercion pubescentis* (*Crataego laevigatae-Quercio cerridis sensu* [43])], and Holm oak Series (*Quercion ilicis*). The meteorological reference point is the thermo-pluviometric station of *Monte Mario* (143 m a.s.l.); the thermo-pluviometric diagram (Figure 10) shows mean temperatures of the coldest (January) and the hottest (July) months of, respectively, 7.4 and 24.0 °C; mean annual rainfall is 766.0 mm [maximum monthly value in November (113.0 mm), minimum value in July (17.0 mm)]; aridity is about 2 months.

### 3.5. Landscape Remarks and Actual Vegetation Physiognomies

**Figure 11:**
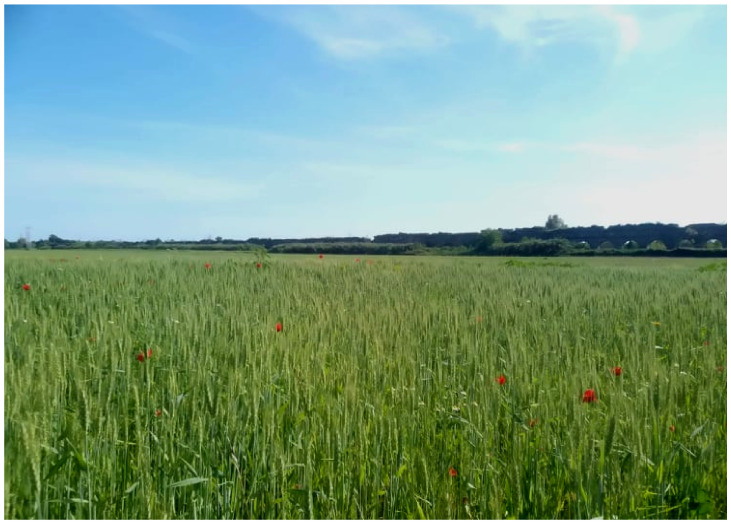
Landscape elements of Appia Antica Regional Park: matrix. Wheat field in locality Acquedotti.

**Figure 12:**
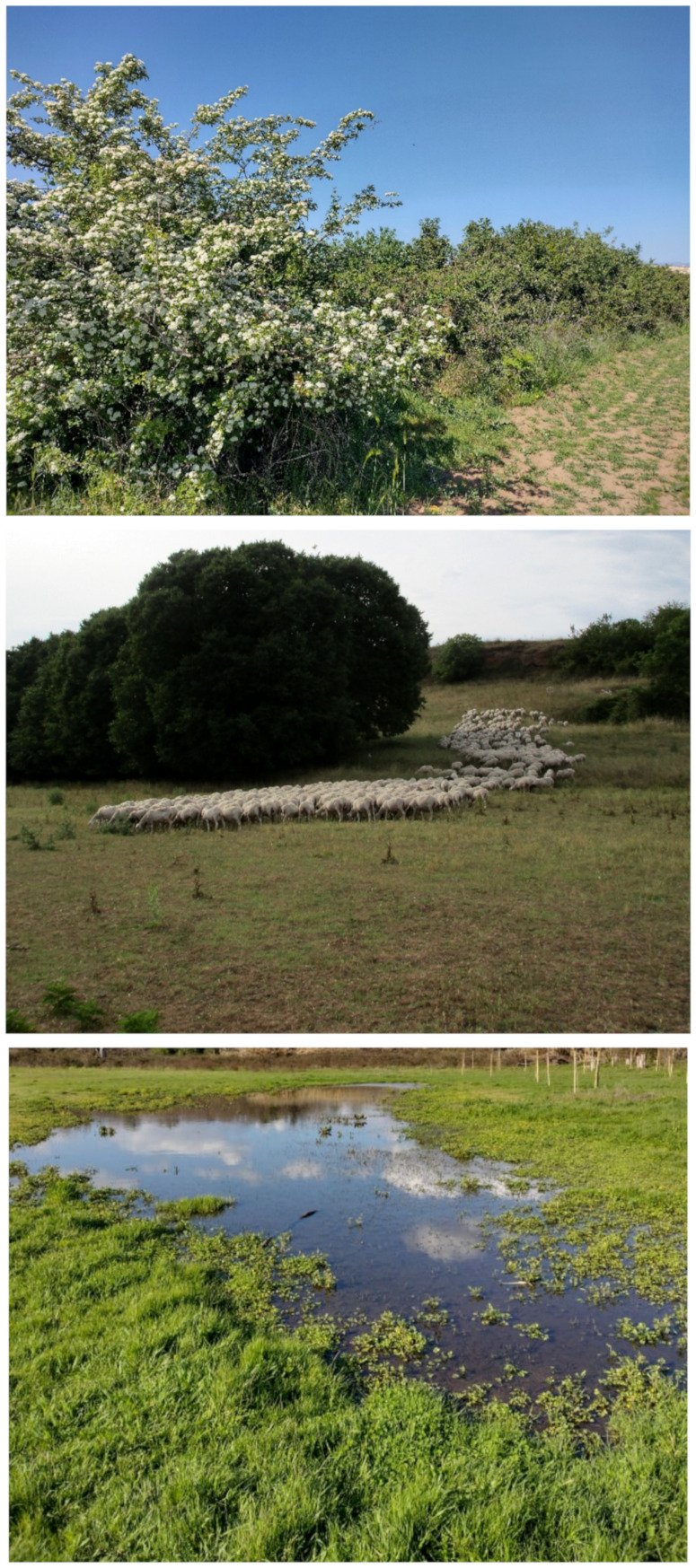
Landscape elements of Appia Antica Regional Park: patches. Shrubs adjacent to crops (top photo); pasture (central photo); humid meadow (bottom photo).

**Figure 13:**
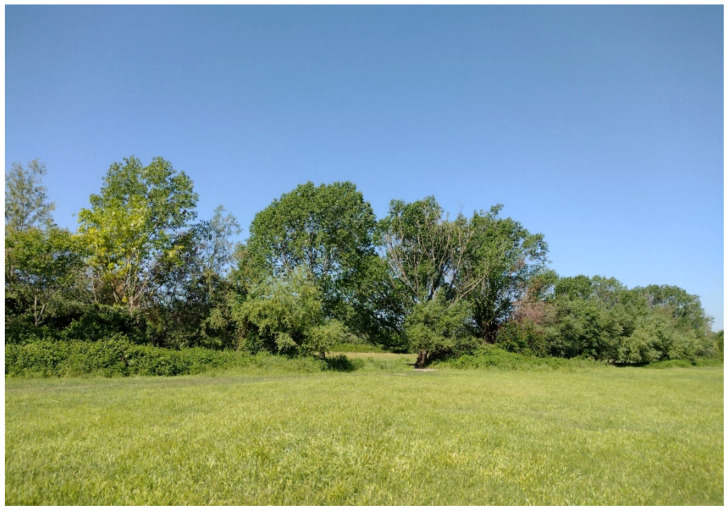
Landscape elements of Appia Antica Regional Park: corridors. Riparian forest in Caffarella valley.

The landscape of Appia Antica Regional Park is defined by an agricultural matrix (primarily wheat fields; Figure 11) that covers more than 50% of the total area. Natural patches are mostly *remnant*-type according to Forman and Godron [52], being caused by widespread disturbance from the matrix. These patches are represented in the study area by shrubs or forest residual areas (Figure 12) that resulted after both agricultural activities and grazing (sheep and cows) (the so-called *Anthropic determinism sensu* [53]). Other patches related to human activities, but not residual, are the mesophilous meadows used as pastures (Figure 12) and/or for recreational activities. In further cases, patches exist since the natural environmental conditions did not allow easy and favorable land uses (the so-called *Natural determinism sensu* [53]). Examples are the humid meadow areas occurring in the Almone valley (north of the park) which remain due to the near-surface aquifer (Figure 12). Concerning the landscape corridors, they are represented mainly by channels (locally named *Marrane*) with banks covered by both herbaceous or shrubby vegetation only and riparian forests (Figure 13).

**Figure 14:**
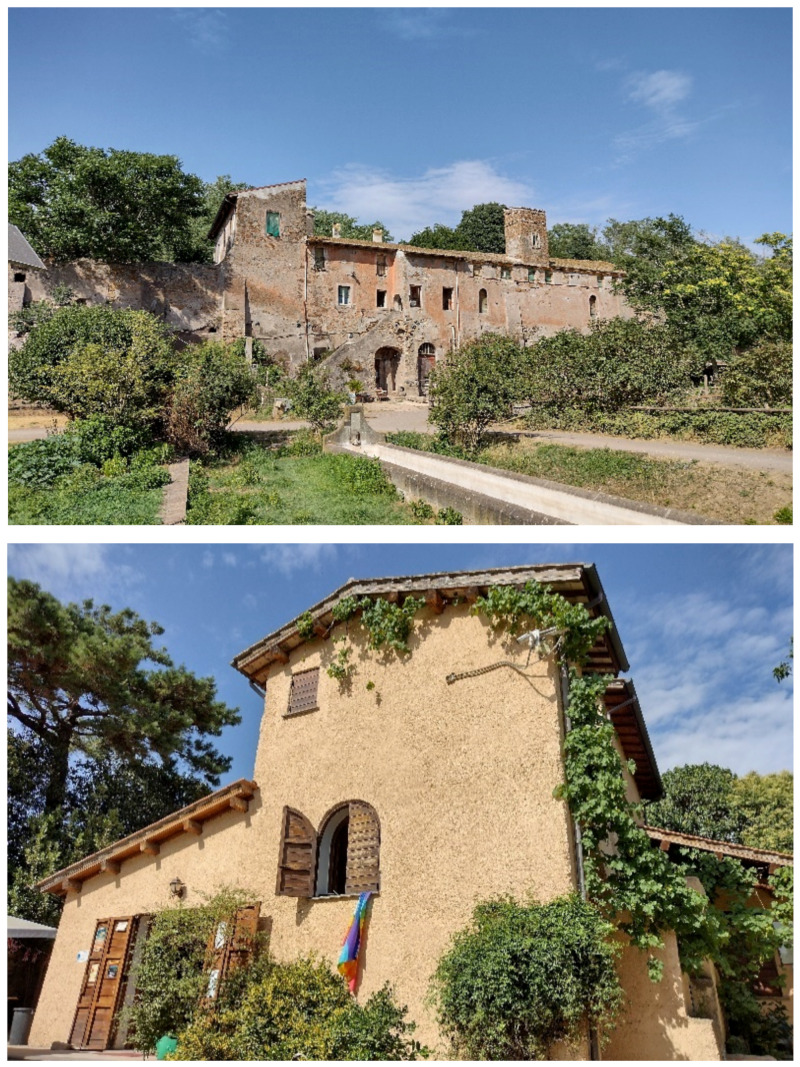
Historical farmhouses of Appia Antica Regional Park landscape: *Vaccareccia* (top photo); *Vigna Cardinali* (bottom photo).

**Figure 15:**
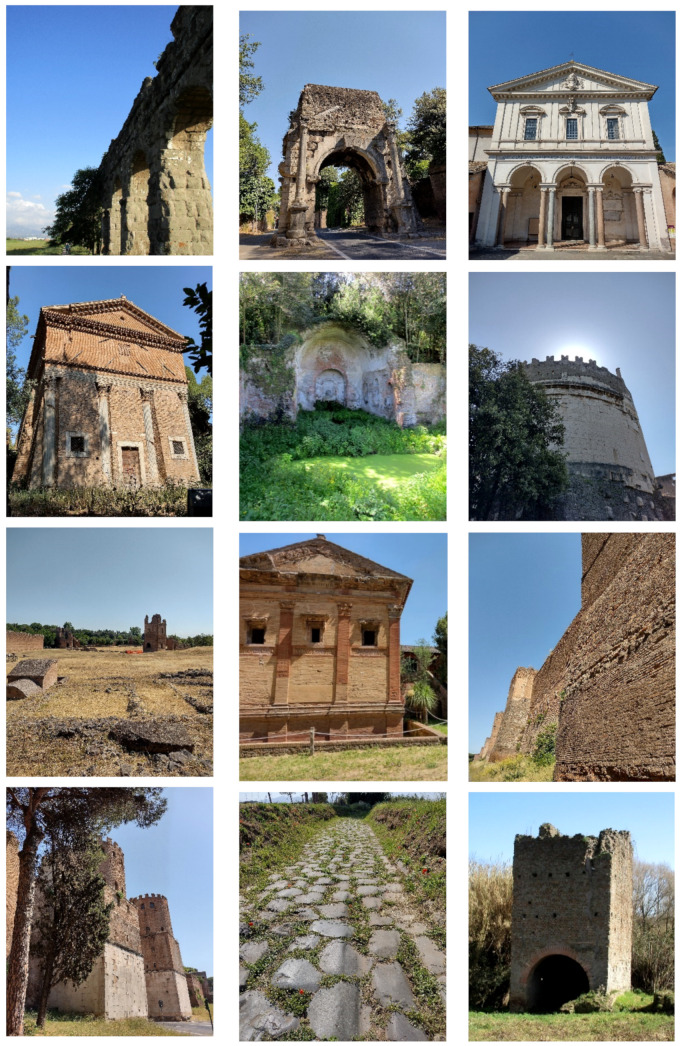
Archeological elements of Appia Antica Regional Park landscape. First row (from left to right): *Aqua Claudia* aqueduct; *Druso*’s Arch (*Antoniano* aqueduct); catacomb *San Sebastiano* (entrance). Second row: church *Sant’Urbano*; *Egeria* nymphaeum; *Cecilia Metella* sepulcher. Third row: *Massenzio* ruins; temple of *God Redicolo*; *Aureliane*’s wall. Fourth row: *San Sebastiano*’s door; *Latina* way; medieval tower *Valca*.

In addition to the natural and seminatural patches, the landscape of the park is characterized by many historical and archeological elements (Figure 14 and Figure 15). The former are represented mainly by farmhouses (e.g., *Vaccareccia* (16th century), *Ex Mulino* and *Vigna Cardinali* (19th century)). The archeological elements consist of many types of monuments, e.g., aqueducts (e.g., *Aqua Claudia* (38–52 a.C.)), *Felix Aqueduct* (around half of the 1st century a.C.), *Antoniano Aqueduct* (around half of 2nd century a.C.)), catacombs (e.g., *San Callisto*, *San Domitilla*, *San Sebastiano* (half of 2nd century a.C.)], churchs [*Domine Quo Vadis* (medieval), *Sant’Urbano* (6th century a.C.)), nymphaeum (*Egeria nymphaeum*, 2nd century a.C.), tombs and sepulchres (e.g., *Latin tombs* (1st–2nd century a.C.), *Cecilia Metella sepulchre* (30–10 a.C.)), palaces (e.g., *Circus* and *Massenzio’s imperial palace*, 2nd–3rd century a.C.), temples (e.g., *temple of God Redicolo*, second half of the 2nd century a.C.), walls (e.g., *Aureliane’s wall* with *San Sebastiano door* (270–275 a.C.)), ways (e.g., *Appia Antica* (end of 4th century a.C.), *Latina* (end of 4th century a.C. to beginning of 3rd century a.C.)); medieval towers or fortifications (e.g., Tor Fiscale and Valca towers) also occur.

As a whole, this landscape composition reveals a high landscape fragmentation that occurred during the past, which caused a decrease in the environmental quality of the territory. In addition, the ecologic connectivity appears to be low, especially in the southern part of the park, where few corridors occur and the matrix occupies a higher percentage of the total area than in the northern part.

The natural vegetation of Appia Antica Regional Park is represented by several types, more or less linked to the Mediterranean climate that characterizes the study area. Although a detailed vegetation study of the park is lacking, ongoing surveys (Iamonico in prep.) allow presenting a general view of the main vegetation physiognomies occurring in the park.

**Figure 16:**
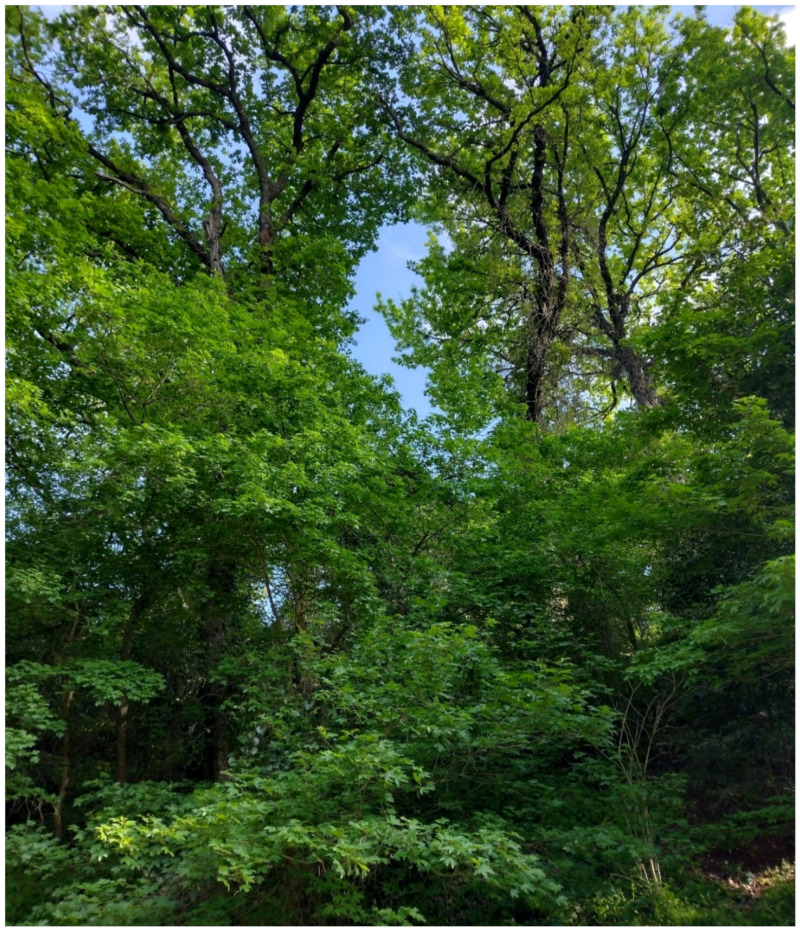
Broad-leaved forests (*Quercetalia pubescenti-petraeae*) in Caffarella valley.

**Figure 17:**
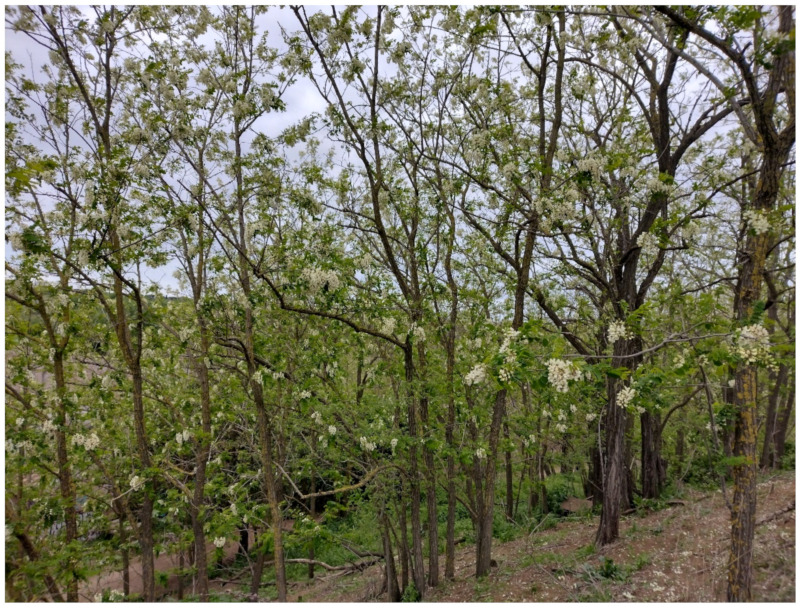
Anthropogenic woody vegetation (Robinieta): *Robinia pseudoacacia* dominated community (Caffarella valley).

**Figure 18:**
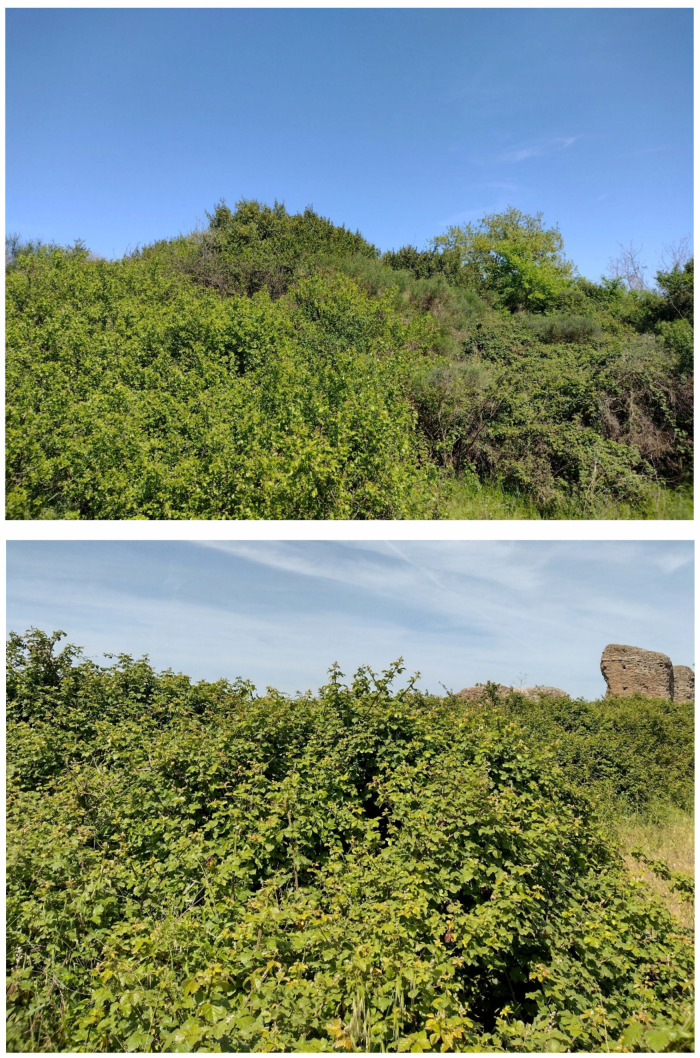
Scrub vegetation of the nemoral zone (Crataego-Prunetea): mixed scrub, locality Caffarella (top photo); monophytic communities with *Rubus ulmifolius*, locality Acquedotti (bottom photo).

**Figure 19:**
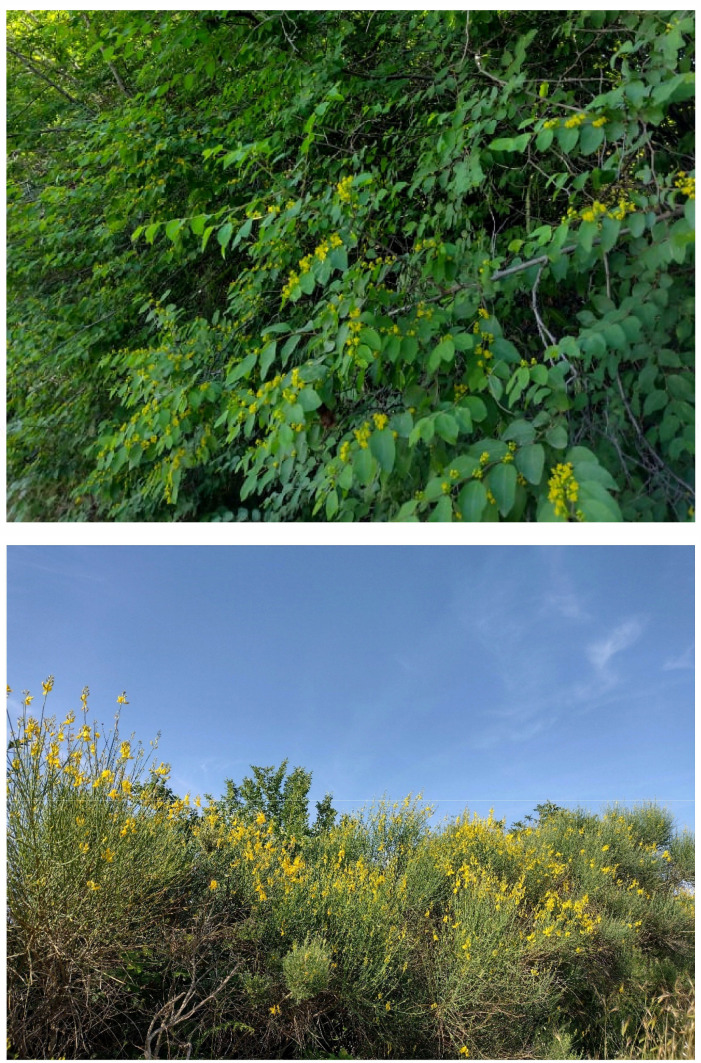
Scrub vegetation in the nemoral zone (Crataego-Prunetea): *Paliurus spina-christi* community, locality Caffarella (top photo); *Spartium junceum* community, locality Tor Marancia (bottom photo).

**Figure 20:**
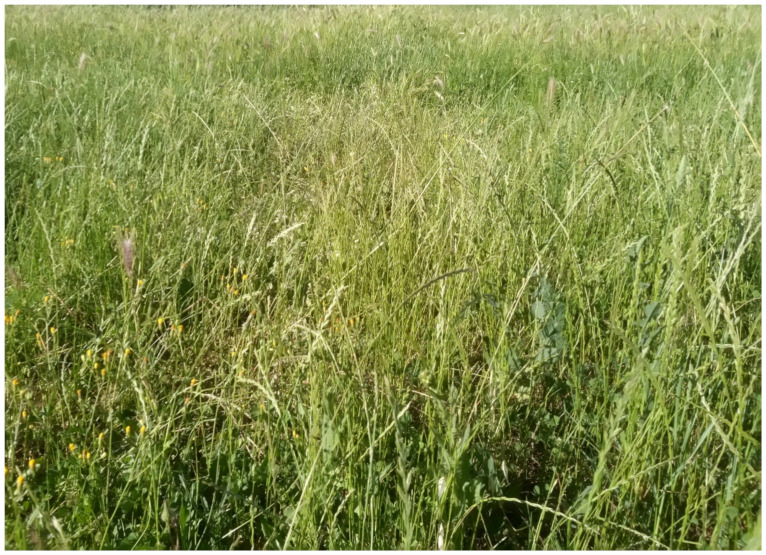
*Lolium perenne* dominated community, Cynosurion cristati (Divino Amore locality).

**Figure 21:**
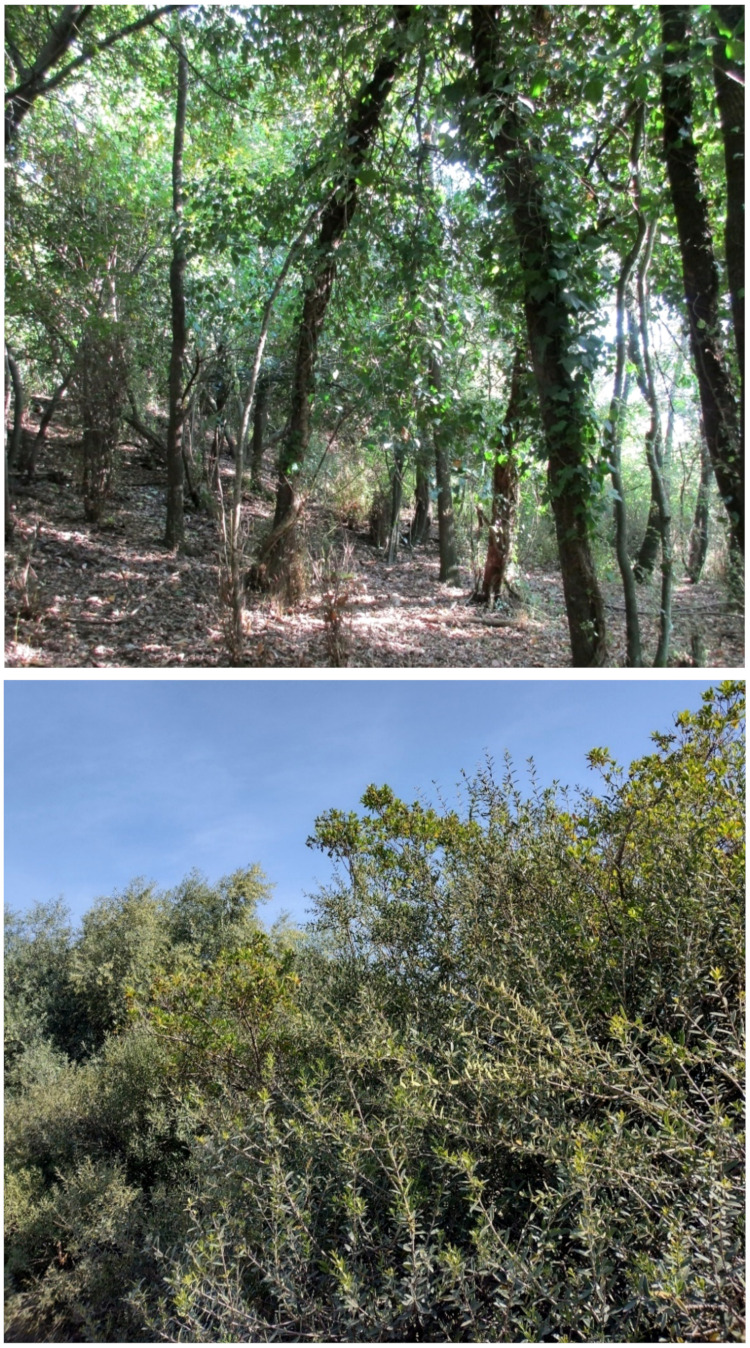
Quercetea ilicis: Olm Hoak forest in Caffarella valley (top photo); Mediterranean macchia in Acquedotti locality (bottom photo).

**Figure 22:**
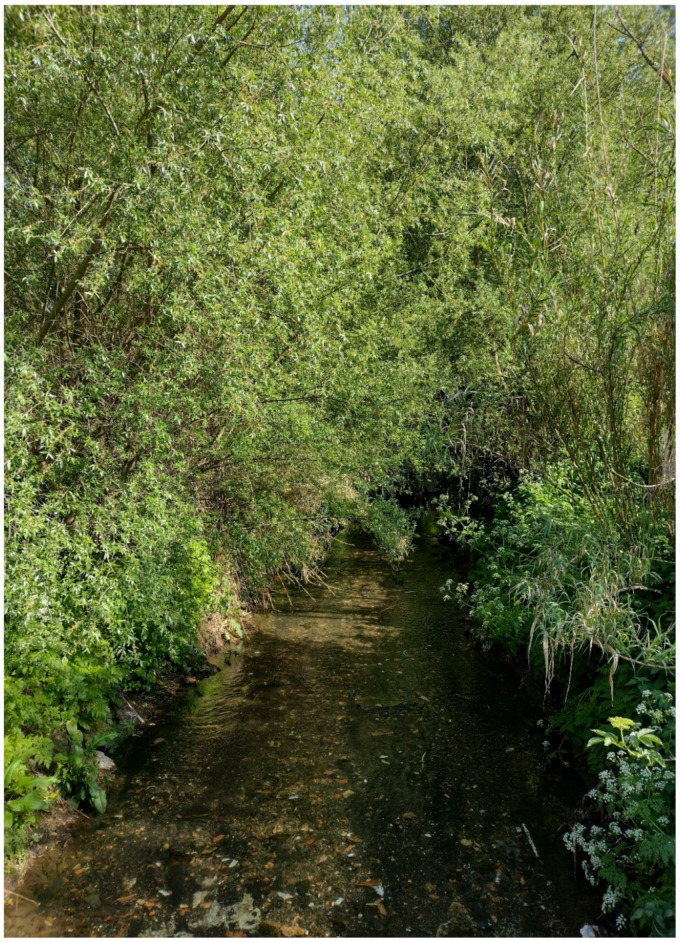
Riparian forest along river Almone (Caffarella locality).

**Figure 23:**
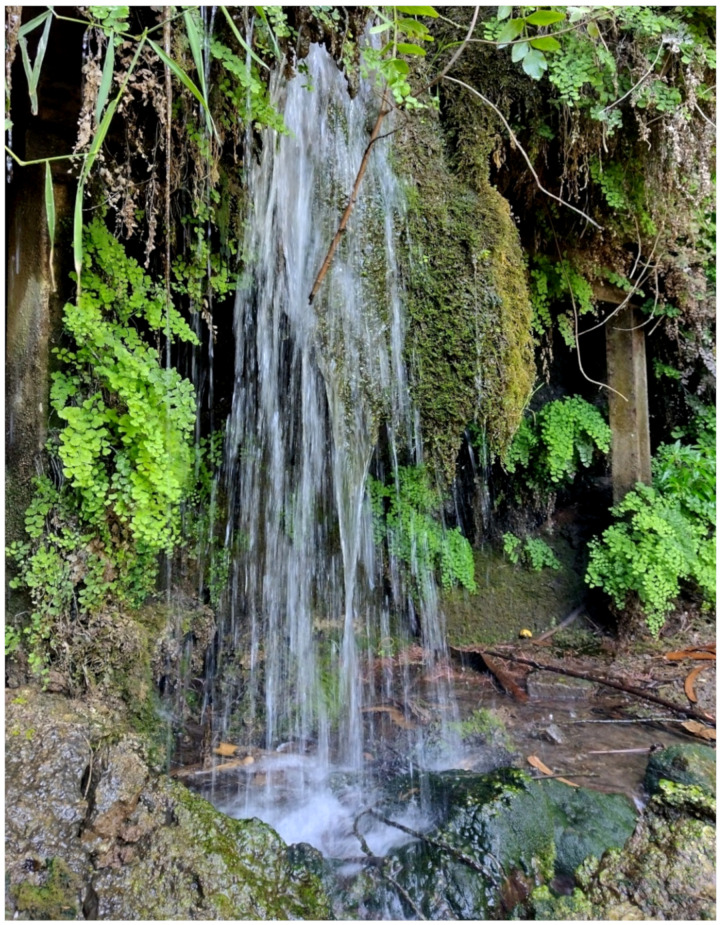
Adiantetea on the volcanic cliffs of channel Acqua Mariana (Acquedotti locality).

**Figure 24:**
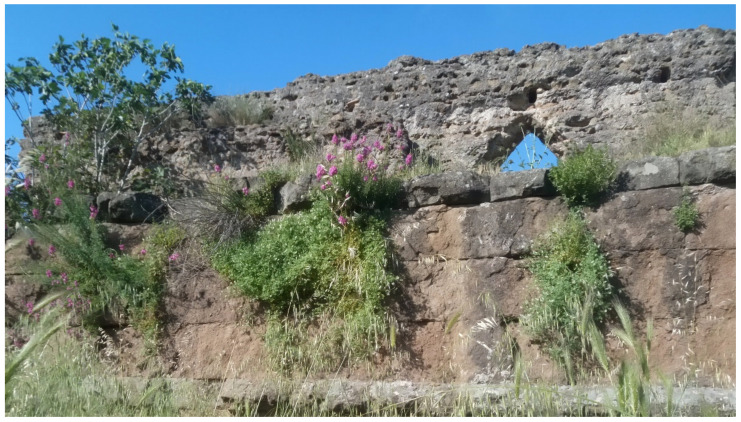
Cymbalario-Parietarietea diffusae on the ancient Roman Claudio aqueduct (Acquedotti locality).

**Figure 25:**
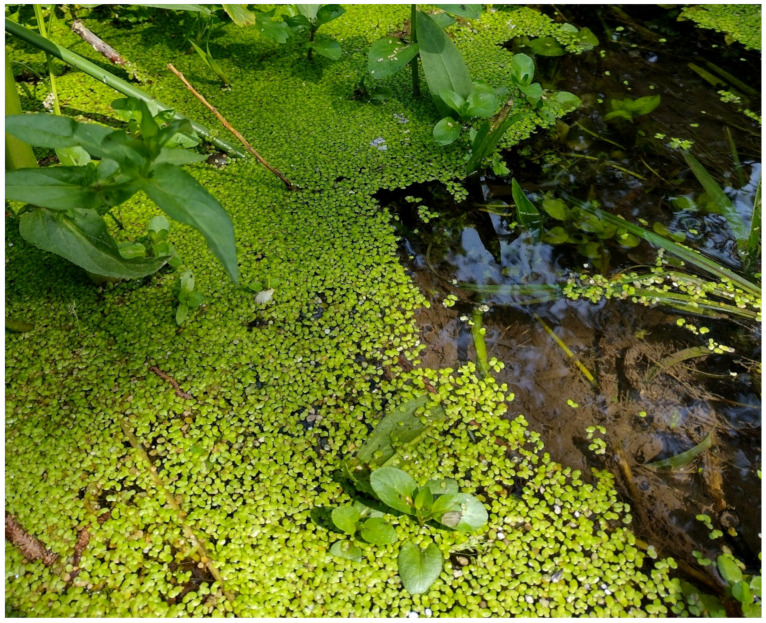
*Lemna minuta* community, Lemnetea (Acquedotty locality).

**Figure 26:**
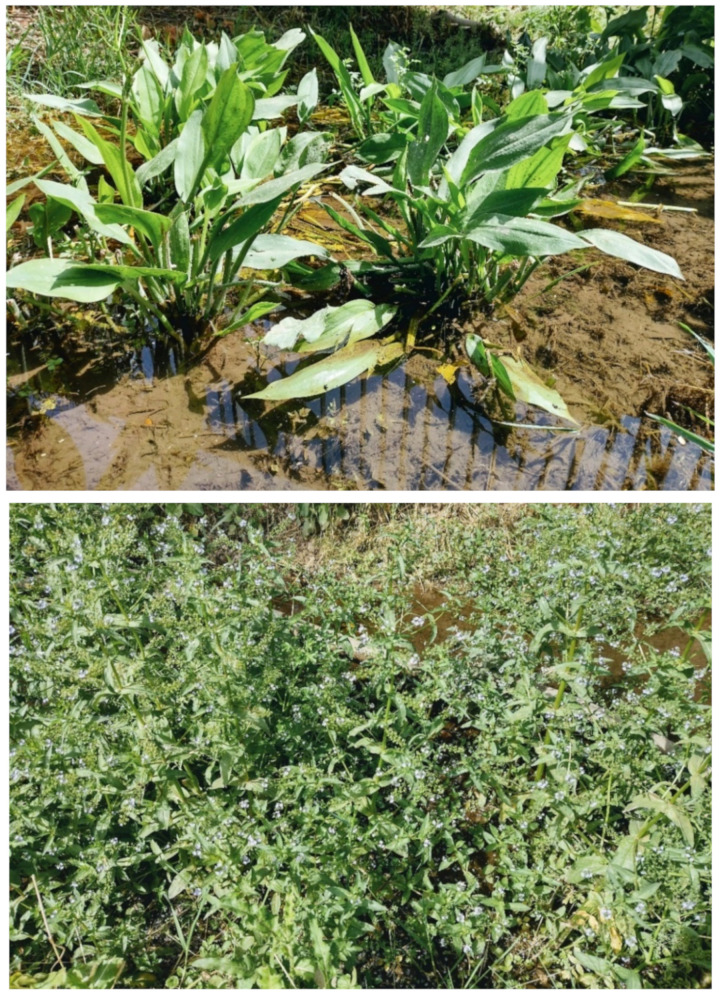
Phragmito-Magnocaricetea on channel Acqua Mariana (Acquedotti locality) with *Alisma plantago-aquatica* (top photo) and *Veronica anagallis-aquatica* dominated (bottom photo).

**Figure 27:**
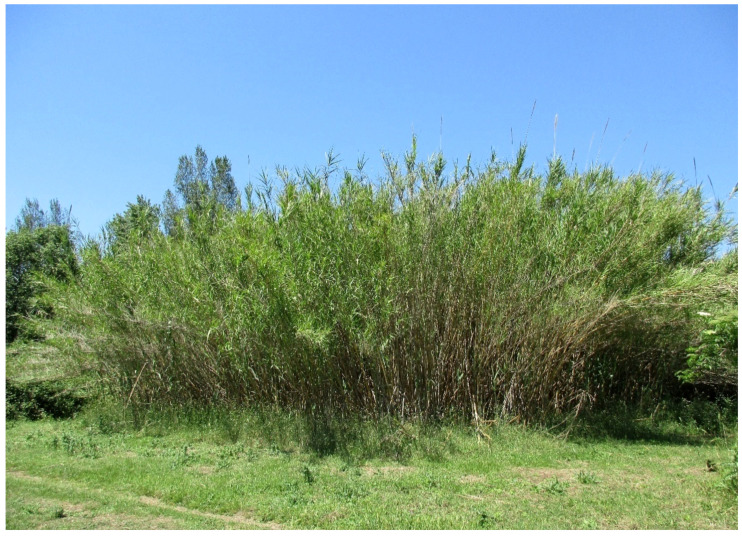
*Arundo donax* community (Divino Amore locality).

**Figure 28:**
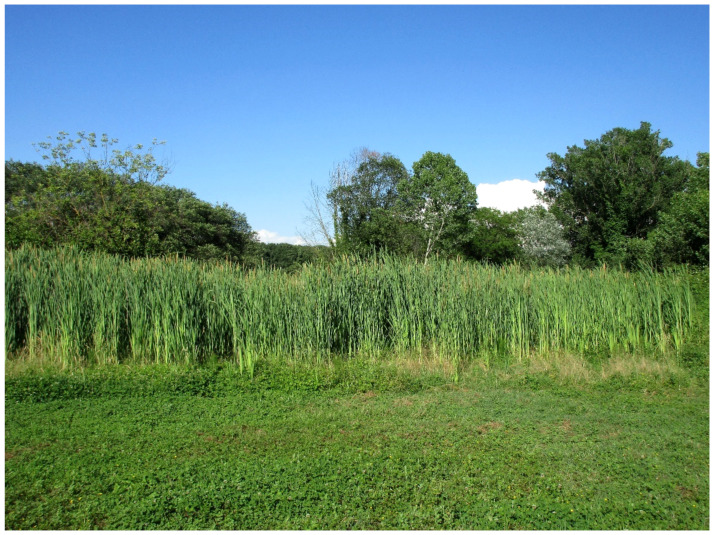
*Typha latifolia* community (Caffarella valley).

**Figure 29:**
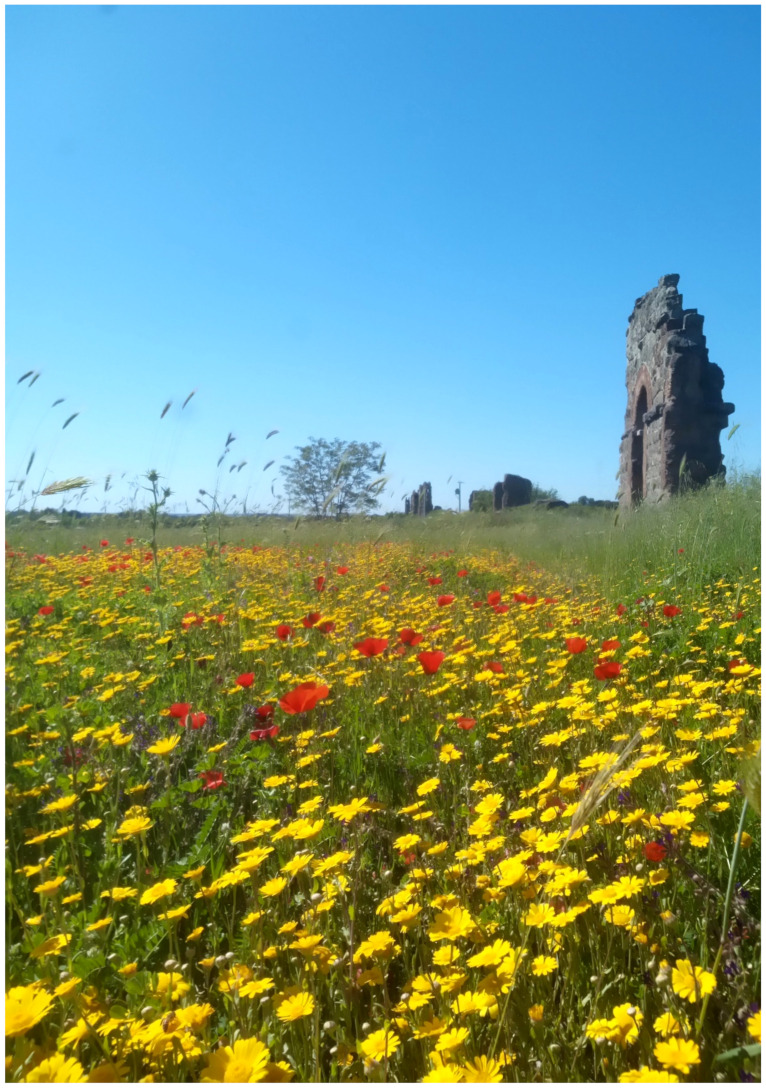
Segetal vegetation dominated by *Glebionis segetum* (L.) Fourr. and *Papaver rhoeas* L., Papaveretea rhoeadis (Acquedotty locality).

**Figure 30:**
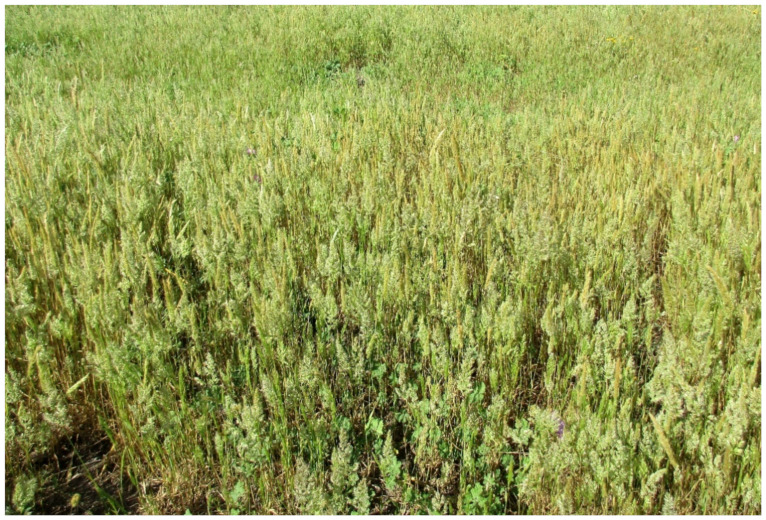
*Trisetaria panicea* dominated community, Sisymbrietea (Caffarella locality).

**Figure 31:**
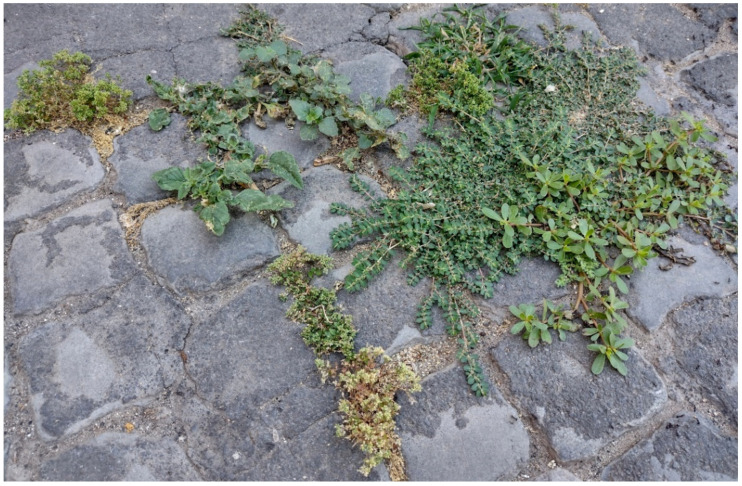
Polygono-Poetea annuae: therophyte communities on crevices of paved Appia Antica street; occurred taxa: *Amaranthus retroflexus* L., *Cynodon dactylon* (L.) Pers., *Euphorbia prostrata* Aiton, *Herniaria glabra* L. subsp. *glabra*, *Polycarpon tetraphyllum* subsp. *tetraphyllum*, *Portulaca oleracea* L. subsp. *oleracea*, *Solanum nigrum* L.

**Figure 32:**
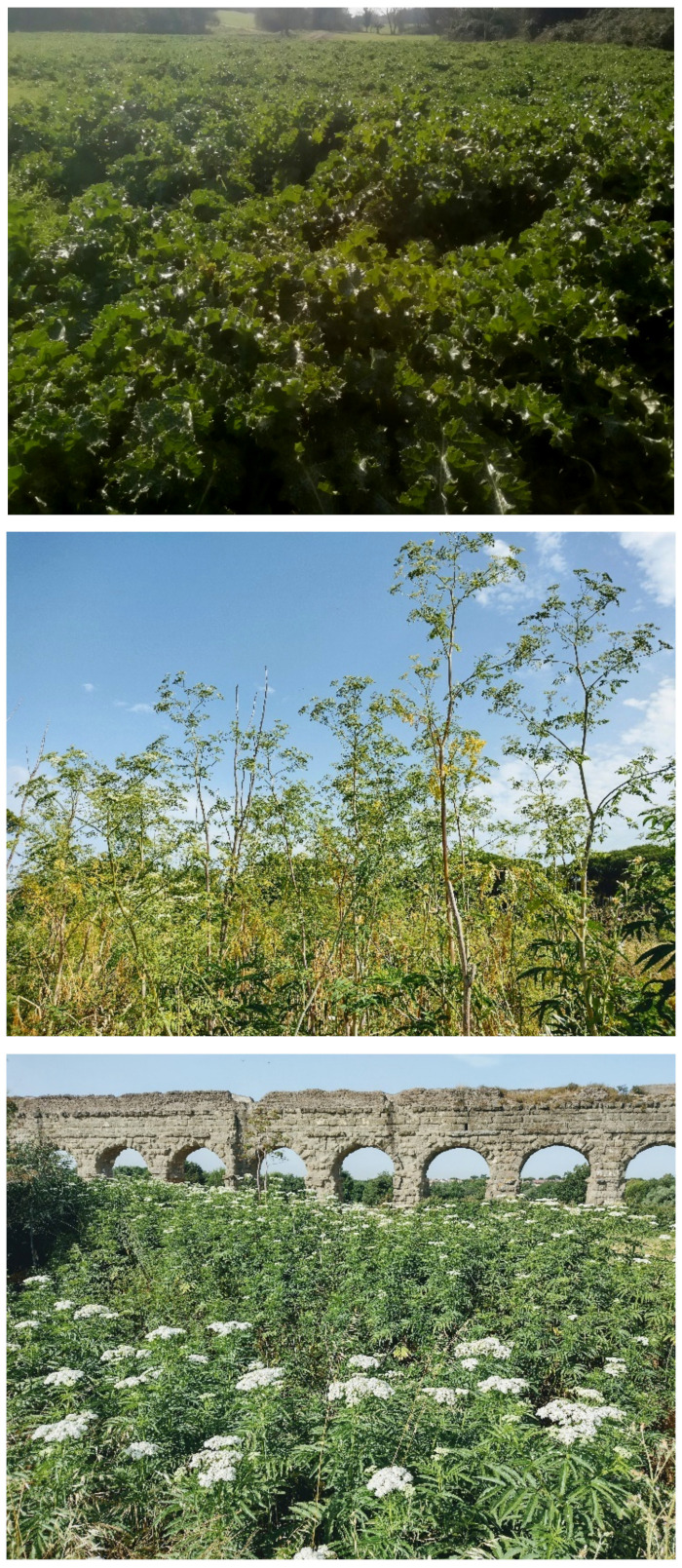
Artemisietea vulgaris: *Silybum marianum* community, Caffarella valley (top photo); *Conium maculatum* community, Acquedotti locality (central photo); *Sambucus ebulus* community, Acquedotti locality (bottom photo).

**Figure 33:**
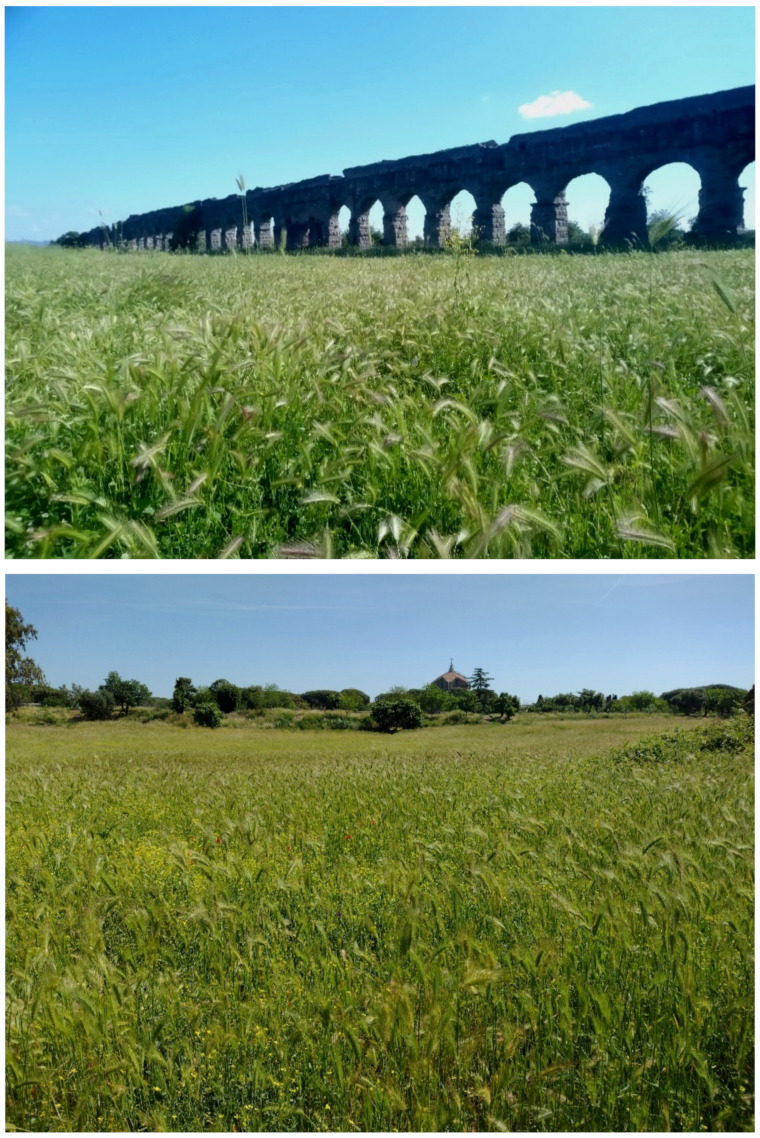
Chenopodietea: Hordeion-murini (Acquedotty locality; top photo); Securigero securidacae-Dasypyrion villosi (Acquedotti locality; bottom photo).

**Figure 34:**
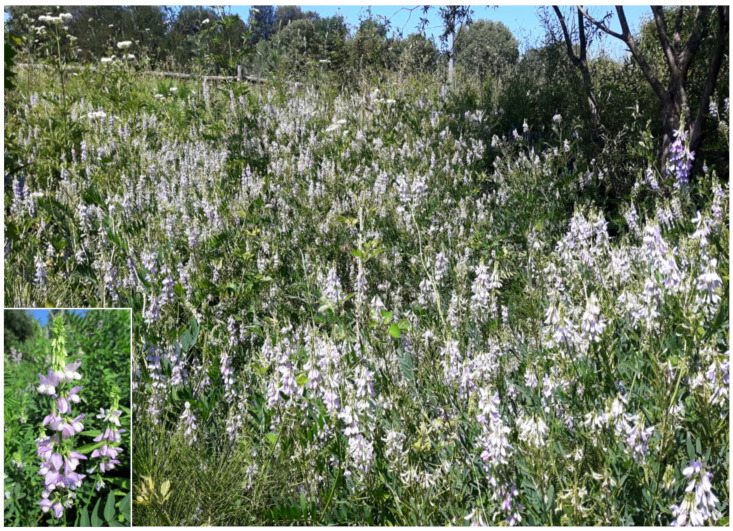
*Galega officinalis* L. dominated community, Epilobietea angustifolii (Acquedotti locality).

⮚ Zonal vegetation:
  - Vegetation of the nemoral forest zone:
    > Zonal temperate broad-leaved forests:
      1. Quercetea pubescentis Doing-Kraft ex Scamoni et Passarge 1959 (Figure 16): mixed forest communities of deciduous species mainly occurring in northern areas of the park; common species are: *Quercus ilex* L. subsp. *ilex*, *Q. pubescens*, *Q. petraea* (Matt.) Liebl., *Fraxinus ornus* L., *Acer campestre* L. among trees, and *Crataegus monogyna* Jacq., *Euonymus europaeus* L., *Cornus sanguinea* L. subsp. *sanguinea*, and *Viburnum tinus* L. among shrubs;
      2. Quercetea robori-petraeae Br.-Bl. et Tx. ex Oberd. 1957: a small forest dominated by *Quercus robur* L. subsp. *robur* occurs in the northern area of the park (*Cartiera Latina* locality);
    > Intrazonal scrub and woodlands of the nemoral zone:
      3. Robinietea Jurko ex Hadac et Sofron 1980: it comprises anthropogenic woody vegetation (Figure 17) characterized by an high presence of *Robinia pseudoacacia* L. and or *Ailanthus altissima* (Mill.) Swingle. Other common species are: *Sambucus nigra* L., *Ulmus minor* Mill. subsp. *minor* (among trees), *Rubus ulmifolius* Schott (among shrubs); herb layer is dominated by nitrophilous taxa, e.g., *Galium aparine* L. and *Urtica dioica* L. These types of vegetation occur sparsely throughout the study area;
      4. Crataego-Prunetea Tx. 1962: scrub vegetation occurring as patches among the cultivated fields or along the margins of the forests. The main communities occurring in the study area are those with *Cornus sanguinea* subsp. *sanguinea*, *Crataegus monogyna*, *Euonymus europaeus*, *Prunus spinosa* L. subsp. *spinosa*, *Rhamnus alaternus* L. subsp. *alaternus*, *Rosa canina* L., *Rubus ulmifolius*, etc. (Figure 18), whereas in the areas more affected by human pressures, monophytic communities with *Rubus ulmifolius* occur (Figure 18). Further shrub communities are those dominated by *Paliurus spina-christi* Mill. (which can be found in Caffarella valley in areas that are not or only marginally affected by pasture; Figure 19), *Spartium junceum* L. (often on the top of cliffs; Figure 19), and groups of species (*Cornus sanguinea* subsp. *sanguinea*, *Ligustrum vulgare* L., *Euonymus europaeus*, *Crataegus monogyna*, *Ulmus minor* subsp. *minor*) that occupy the anthropic sinkholes originating from the collapse of underground cavities;
    > Intrazonal boreo-temperate grasslands and heath:
      5. Molinio-Arrhenatheretea Tx. 1937: anthropogenic managed pastures, meadows and tall-herb meadow fringes on fertile deep soils at low and mid-altitudes.
        ■ Cool Temperate Group of Alliances:
          ⮚ Cynosurion cristati Tx. 1947 (Arrhenatheretalia elatioris Tx. 1931): mesophilous grasslands, grazed and mown once, growing on well-drained mineral/nutrient-rich soils. These communities are common throughout the park, particularly in the areas affected by human recreational use (Figure 20).
  - Vegetation of the mediterranean zone:
    > Zonal mediterranean forests and scrub:
      6. Quercetea ilicis Br.-Bl. ex A. Bolós et O. de Bolós in A. Bolós y Vayreda 1950: thermo-mesomediterranean oak forests and associated Mediterranean macchia. Small forest patches with *Quercus ilex* subsp. *ilex* (Figure 21) or *Q. suber* L. and Mediterranean macchia occur. Holm oak forests are present in Caffarella and Tor Marancia localities. Only one patch of corn oak forest remains (the so-called *Boschetto Farnese* = Farnese’s wood, included in the private farmstead “Farnesiana”); this forest is represented by a mixed evergreen/deciduous species where *Q. suber* grows together with *Q. pubescens* and *Q. ilex* subsp. *ilex*, whereas the shrub layer is composed by *Ulmus minor* subsp. *minor*, *Rubus ulmifolius*, *Crataegus monogyna*, *Prunus spinosa* subsp. *spinosa*, *Euonymus europaeus*, *Rhamnus alaternus* subsp. *alaternus*, *Cornus sanguinea* subsp. *sanguinea*, and *C. mas* L. Finally, the residual patches of Mediterranean macchia, which are dominated by *Arbutus unedo* L., *Phillyrea latifolia* L., and *Quercus ilex* subsp. *ilex* (Figure 21).
  - Azonal vegetation:
    > Alluvial forests and scrub:
      7. Alno glutinosae-Populetea albae P. Fukarek et Fabijanic 1968: Mediterranean riparian communities on soils with high water table; this type is well represented in the park along rivers and channels, especially in the central and north zones of Caffarella valley and Tor Marancia locality, where forests are dominated by *Populus nigra* L., *Salix alba* L. subsp. *alba*, and *Fraxinus angustifolia* Vahl. subsp. *oxycarpa* (M.Bieb ex Willd.) Franco & Rocha Afonso (Figure 22).
  - Vegetation of rock crevices and screes:
    > Adiantetea Br.-Bl. et al. 1952: communities dominated by *Adiantum capillus-veneris* L. and bryophytes that grow on siliceous dripping cliffs; they are common along deeper channels and springs (Figure 23).
      8. Cymbalario-Parietarietea diffusae Oberd. 1969: thermo-nitrophilous Mediterranean chasmophytic vegetation of walls and cliffs; quite distributed on both natural (volcanic cliffs) and anthropic (aqueducts, walls) surfaces. Frequent species are: *Antirrhinum majus* L., *Capparis orientalis* Veill., *Cymbalaria muralis* G.Gaertn., B.Mey. & Schreb. subsp. *muralis*, *Ficus carica* L., *Fumaria capreolata* L., *Parietaria judaica* L., *Reichardia picroides* Roth, *Sonchus tenerrimus* L., *Umbilicus rupestris* (Salisb.) Dandy (Figure 24).
  - Freshwater aquatic vegetation:
      9. Lemnetea O. de Bolos et Masclans 1955: pleustophytic vegetation that colonizes fresh waters; in the territory of the park, these communities mainly occur on weakly flowing waters (channels or lakes) where the alien *Lemna minuta* Kunth dominates the autochthonous *L. minor* L. (Figure 25).
  - Vegetation of freshwater springs, shorelines and swamps.
      10. Phragmito-Magnocaricetea Klika in Klika et Novak 1941: perennial helophytic communities colonizing lacustrine and fluvial areas on eu- to mesotrophic soils of freshwater bodies; the communities mostly occur in the northern sector of the park along channels or around ponds and artificial lakes (Figure 26). Common species are: *Alisma plantago-aquatica* L., *Apium nodiflorum* (L.) Lag., *Arundo donax* L. (which often forms monophytic communities; Figure 27), *Equisetum telmateja* Ehrh., *Lymniris pseudacorus* (L.) Fuss., *Nasturtium officinalis* R.Br., *Phragmites australis* (Cav.) Tin. ex Steud., *Symphytum officinale* L., *Typha latifolia* L. (which sometimes forms monophytic communities; Figure 28), *Veronica anagallis-aquatica* L.
  - Anthropogenic vegetation: secondary vegetation communities that derive from direct or indirect results of human action; they are represented by several types occurring throughout the study area; most of these types are meadow for-mations. The main types observed are listed as follows:
      11. Papaveretea rhoeadis S. Brullo et al. 2001 (= Secalinetea Br.-Bl. In Br.-Bl. et al. 1952 = Stellarietea mediae Tx. et al. in Tx. 1950): annual weed segetal vegetation of arable crops on base-rich soils; it is widely distributed in Appia Antica Regional Park since crops represent the landscape matrix (Figure 29).
      12. Sisymbrietea Gutte et Hilbig 1975: anthropogenic vegetation of animal shelters and disturbed ruderal sites (Figure 30).
      13. Polygono-Poetea annuae Rivas-Mart. 1975: nitrophilous pioneer vegetation of trampled habitats. This type of vegetation is common, especially on roadsides and crevices of paved roads (Figure 31).
      14. Artemisietea vulgaris Lohmeyer et al. in Tx. ex von Rochow 1951: perennial meso-xerophilous ruderal vegetation. The most common communities found in the park are dominated by *Silybum marianum* (L.) Gaertn. (margins of fields and uncultivated lands; Figure 32), *Conium maculatum* L. (banks of water courses; Figure 32), and *Sambucus ebulus* L. (humid and disturbed soils near rivers and channels; Figure 32).
      15. Chenopodietea Br.-Bl. in Br.-Bl. et al., 1952: weed segetal and ruderal vegetation of man-made habitats. This group includes many types in the park, the most common are the Mediterranean annual grasslands of Hordeion murini Br.-Bl. in Br.-Bl. et al. 1936 and Securigero securidacae-Dasypyrion villosi Cano-Ortiz, Biondi et Cano in Cano-Ortiz et al. ex Di Pietro in Di Pietro et al., 2015. The former is represented by ruderal grasslands occurring in nutrient-rich soils characterized by a high presence of humans (Figure 33). The second group (Securigero securidacae-Dasypyrion villosi) includes the therophytic anthropogenic grasslands in fallow-land habitats of the central regions of the Apennine Peninsula; this type of vegetation is dominated by *Dasypyrum villosum* (L.) P.Candargy (Figure 33), representing one of the most widespread anthropogenic steppe formations in the Roman countryside [54].
      16. Epilobietea angustifolii Tx. et Preising ex von Rochow 1951 (=Galio-Urticetea Passarge 1967): tall-herb semi-natural perennial vegetation on disturbed forest edges and nutrient-rich riparian fringes (Figure 34).

## 4. Results

### 4.1. General Statistics of the Flora

The flora of Appia Antica Regional Park is composed by 714 taxa (including species and subspecies; see Appendix A), belonging to 104 families and 403 genera. These include 11 Lycopodiophyta and ferns and allies (six families and seven genera), three gymnosperms (*Pinus pinea* L., *P. halepensis* Mill. and *Cupressus sempervirens* L., as aliens), one Magnoliidae (*Laurus nobilis* L.), 557 angiosperm eudicots (81 families, 308 genera), and 142 angiosperm monocots (13 families, 85 genera).

**Figure 35:**
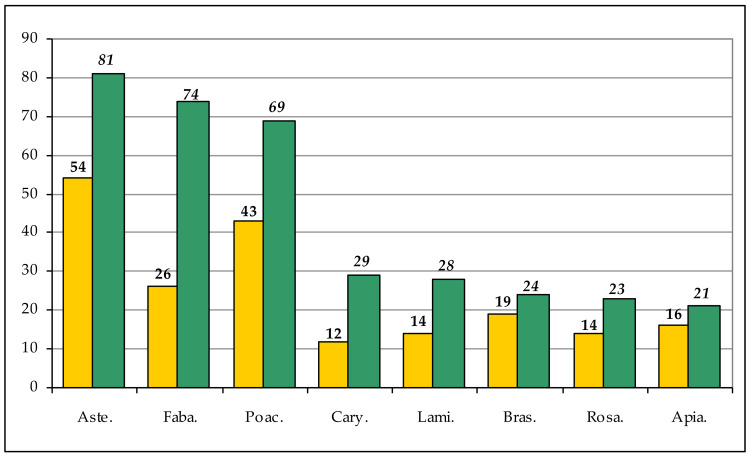
Number of genera (orange columns) and taxa (dark-green columns) per family; only the richest families (more than 20 taxa (species and subspecies)) are displayed. Abbreviations: Aste. = Asteraceae; Faba. = Fabaceae; Poac. = Poaceaeae; Cary. = Caryophyllaceae; Lami. = Lamiaceae; Bras. = Brassicaceae; Rosa. = Rosaceae; Apia. = Apiaceae.

The richest families are (Figure 35): Asteraceae Bercht. & J.Presl (81 taxa; 54 genera), Fabaceae Lindl. (74 taxa; 26 genera), and Poaceae Barnhart (69 taxa; 43 genera), followed by Caryophyllaceae Juss. (29 taxa; 12 genera), Lamiaceae Martinov (28 taxa; 14 genera), Brassicaceae Burnett (24 taxa; 19 genera), Rosaceae Juss. (23 taxa; 14 genera), and Apiaceae Lindl. (21 taxa; 16 genera). Eleven families include 10 (Chenopodiaceae Vent. and Euphorbiaceae Juss.) to 17 (Plantaginaceae Juss.) taxa. Fifty families comprise two to nine taxa. Finally, 34 families are monospecific for the flora.

**Figure 36:**
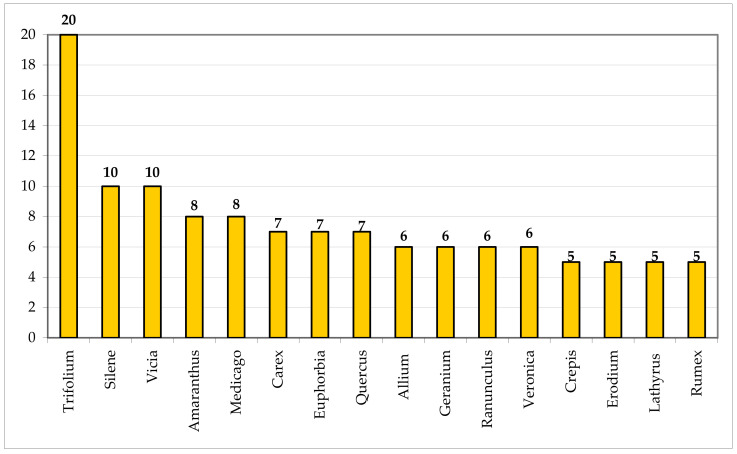
Number of taxa per richest genera [more than four taxa (species and subspecies) per genus).

The richest genera are (Figure 36): *Trifolium* (20 taxa); *Silene* and *Vicia* (10); *Amaranthus* and *Medicago* (8); *Carex*, *Euphorbia*, and *Quercus* (7); *Allium*, *Geranium*, *Ranunculus*, and *Veronica* (6); *Crepis*, *Erodium*, *Lathyrus*, and *Rumex* (5).

According to definition given by Fanfarillo and collaborators [54], the segetal flora of Appia Antica Regional Park includes 348 taxa (corresponding to 49.01% of the total flora), of which 14 (1.97% of the total flora) are strictly segetal (species that only occur in segetal habitats), whereas 25 (3.52% of the total flora) are characteristic segetal (species characteristic of segetal habitats that also commonly colonize other habitats) (Table 1). Three species (*Chenopodium vulvaria* L., *Tribulus terrestris* L., and *Xanthium spinosum* L.) are new additions to the Italian segetal flora, being not listed by [55]. The richness (percentage) of the segetal flora of Appia Antica Park is much higher than that of the entire segetal flora of Italy, i.e., 10.10% (percentage of Italian segetal flora over the Italian vascular flora), 1.61% (percentage of characteristic segetal), and 0.59% (percentage of strictly segetal). These data reveal a high rate of agricultural areas in the park, which, in fact, occupy more than 50% of the territory (see above under the Section “3.5. Landscape Remarks and Actual Vegetation Physiognomies”).

### 4.2. Life Form Analysis

**Figure 37:**
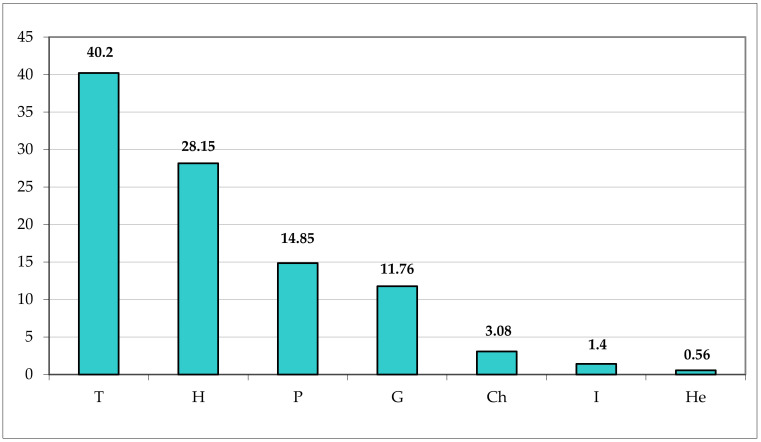
Percentage (axis x) of plant life form spectra of the vascular flora of Appia Antica Regional Park. T: therophytes; H: hemicryptophytes; P: phanerophytes; G: geophytes; Ch: chamaephytes; I: idrophytes; He: helophytes.

**Figure 38:**
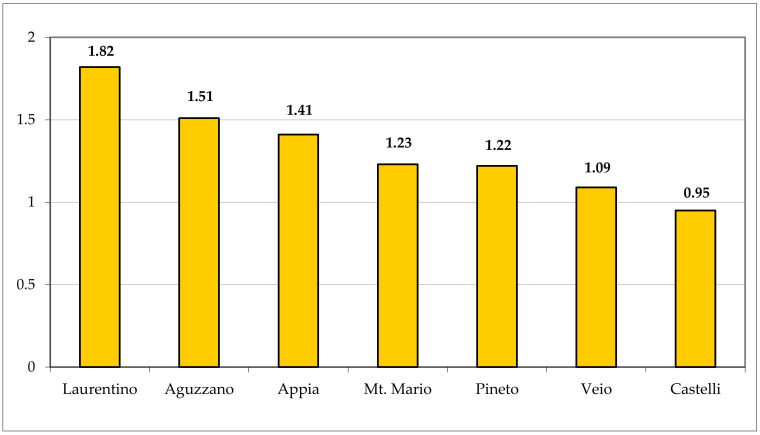
T/H ratio (y axis) of some parks (x axis) in Rome Province.

The vascular flora of Appia Antica Regional Park is characterized by a prevalence of therophytes (287 taxa, corresponding to 40.20% of the total flora), followed by the hemicryptophytes (201 taxa, 28.15%) (Figure 37). The ratio T/H is 1.41, being quite high in comparison with other areas of Rome municipality (Figure 38). These data highlight the xeric conditions of Appia Antica Regional Park in pastures, uncultivated lands, and synanthropic environments that represent the most common habitats. A similar environmental situation occurs in Laurentino and Aguzzano Parks [high therophyte/hemicryptophyte (T/H) ratios], whereas in Monte Mario, Pineto, and Veio Parks (which occur on the north of the Rome area), mesophilous habitats are well represented and the T/H ratio is, therefore, lower due to the higher percentage of hemicryptophytes in comparison with that of the floras of Appia Antica, Laurentino, and Aguzzano Parks. To note, the T/H ratio for Appia Antica Park is also higher than that of the Caffarella valley (1.32), which is included in the studied area, representing about 4% (ca. 190 ha) of the total area.

The phanerophytes in Appia Antica Regional Park, reaching 14.85% (106 taxa) of the total flora, together with a fair percentage of rhizomatous geophytes (42 taxa, 5.88%), typical of forest habitats, highlight the rather rich flora of woody habitats. However, about 1/3 of the phanerophytes (5.04%) are aliens (Table 2), and most of them do not occur in wooded areas [e.g., *Agave americana* L., *Campsis radicans* (L.) Bureau, *Eucalyptus camaldulensis* Dehnh. subsp. *camaldulensis*, *Lantana camara* L, *Maclura pomifera* (Raf.) C.K. Schneid., *Malus domestica* L., *Passiflora caerulea* L., *Ziziphus jujuba* Mill.]. In fact, as a whole, forests represent only residual areas in the territory of the park, and several patches are actually shrubs (see Section “3.5. Landscape Remarks and Actual Vegetation Physiognomies”).

Helophytes and hydrophytes represent 1.96% of the total flora (4 and 10 taxa, respectively) highlighting the presence of aquatic habitats (especially in the northern sector of the park). Of note, among rhizomatous geophytes, several grow in these habitat [e.g., *Adiantum capillus-veneris* L., *Eleocharis palustris* (L.) Roem. et Schult., *Limniris pseudacorus* (L.) Fuss, *Symphytum tuberosum* L. subsp. *angustifolium* (A.Kerner) Nyman, etc.], thus revealing the relevant occurrence of aquatic and strictly related habitats.

Finally, the low percentage of chamaephytes (3.08%, 22 taxa) can be explained by the scarcity of rocky habitats (more widespread in the mountain belt and on windy peaks in Latium region) and the absence of garrigues (occurring especially along coasts).

### 4.3. Geographical Analysis

**Figure 39:**
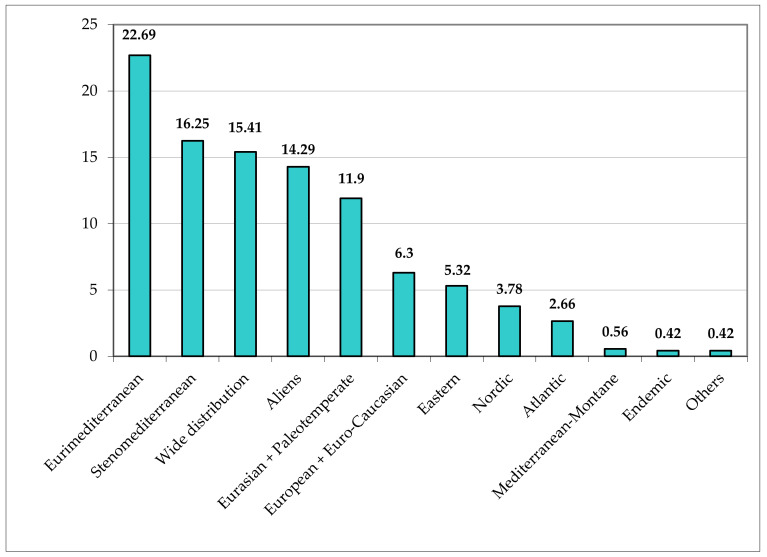
Chorological spectrum of the vascular flora of Appia Antica Regional Park.

**Figure 40:**
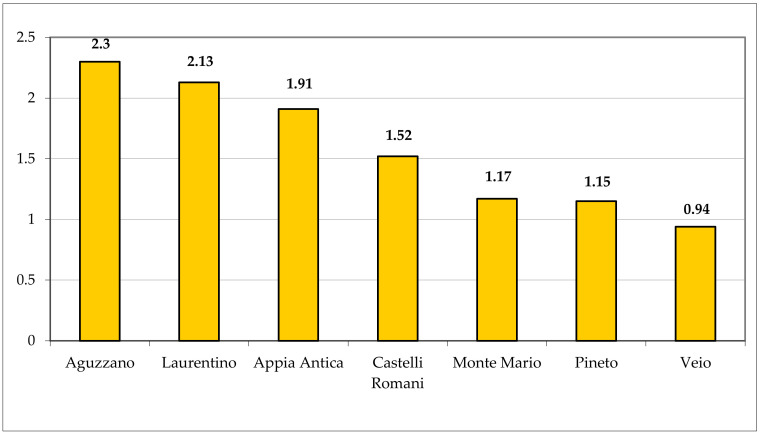
Eurimediterranean/Eurasian ratio (y axis) of some parks (x axis) in Rome Province.

The chorological spectrum (Figure 39) shows a prevalence of Eurimediterranean species (162 taxa, corresponding to 22.69% of the total flora). By considering the Mediterranean component *sensu lato* (Euri- plus Stenomediterranean), the percentage reaches 38.94% [288 taxa (162 euri-, 22.69%; 116 steno-, 16.25%)], which is congruent with the phytoclimatic background of the studied area (Meso-Thermomediterranean climate type and oak series as potential vegetation). This fact is also confirmed by both the high value of the Eurimediterranean/Eurasian species ratio (1.91, which is high in comparison with the ratios of other Parks included in Rome Province; Figure 40), and the low percentage of Nordic species (27 taxa, 3.78%); of note, the Eurimediterranean/Eurasian ratio for Appia Antica Park is also higher than that of the Caffarella valley (1.35), which is included in the studied area, representing about 4% (ca. 190 ha) of the total area. The percentage of the wide distribution species (Cosmopolitan, Subcosmopolitan, and Tropical; 110 taxa) plus the aliens (102 taxa) is high, namely 29.70% (15.41% and 14.29%, respectively); this value is mainly due to human impact, which causes the occurrence and spreading of r-selected species. Finally, the eastern component (SE-European, Pontic, and Turanian s.lat.), typical of the flora of Central Italy and particularly of Lazio, totals 5.32% (38 taxa); similar floristic backgrounds were observed in the whole region (see [26]). Finally, the endemic taxa are very few (only 3 taxa, for 0.42%), probably due to the lack of habitats characterized by biogeographical insularity.

Concerning the alien taxa, they total 102, corresponding to 14.29% of the total flora (native taxa account for 85.71%). Of 52 families, each one contains at least one non-native taxon; 13 of these 46 families are aliens, including one species only (Basellaceae Raf., Bignoniaceae Juss., Cactaceae Juss., Cannaceae Juss., Cupressaceae Gray, Cleomaceae Bercht. & J. Presl, Ebenaceae Gürke, Meliaceae Juss., Nyctaginaceae Juss., Passifloraceae Juss. ex Roussel, Phytolaccaceae R.Br., Pittosporaceae R.Br., Platanaceae T.Lestib.). The families rich in alien species include Asteraceae (10 taxa), followed by Poaceae and Amaranthaceae Juss. (7), Fabaceae (5), Rosaceae and Solanaceae Juss. (4), Araceae Juss., Asparagaceae Juss., Convolvulaceae Juss., Moraceae Gausich., and Oxalidaceae (3), and Apocynaceae Juss., Arecaceae Bercht. & J.Presl, Brassicaceae, Chenopodiaceae Vent., Euphorbiaceae Juss., Pinaceae Spreng. ex F.Rudolphi, and Vitaceae (2); the other families include one alien taxon.

A total of 84 genera include at least one alien taxon. *Amaranthus* is the richest one, with 6 alien species, followed by *Erigeron* L., *Oxalis* L., and *Solanum* L. (3), and *Cuscuta* L., *Dysphania* R.Br., *Euphorbia* L., *Galinsoga* Riuz. & Pav., and *Pinus* L. (2); the other 75 genera each include one alien taxon.

**Figure 41:**
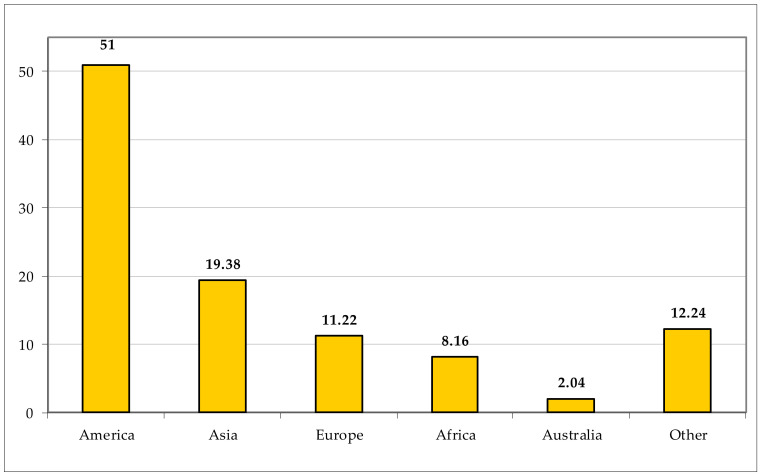
Share of aliens plant species and subspecies (percentages in axis y) by thier per origin.

**Figure 42:**
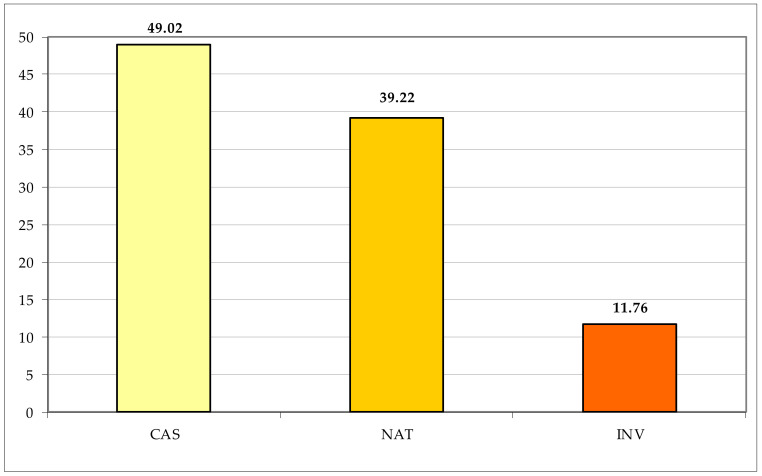
Share of casual (CAS), naturalized (NAT), and invasive (INV) alien plant species and subspecies (percentages in axis y) occurring in Appia Antica Regional Park.

Most of the exotic taxa are native to America (51 taxa, representing 51.00% of the total aliens), followed by the Asia (19 taxa, 19.38%); Europe and Africa, including, respectively, 11 (11.22%) and 8 (8.16%) taxa, and two (2.04%) taxa native to Australia (Figure 41). Concerning the alien status, most of the non-native taxa are casual (50, corresponding to 49.02% of the total aliens) and naturalized (40, 39.22%), whereas only 12 species (11.76%) can be considered as invasive (Figure 42).

### 4.4. Floristic Notes

More than 10 years of field surveys allowed the discovery of several floristic novelties or confirmations at the European (1 species), national (3 species), regional (10 species), and local (7 species) levels; all of these novelties refer to alien species (Table 3; see Appendix B).

#### 4.4.1. New Record for Europe and First One Out of the Native Range

**Figure 43:**
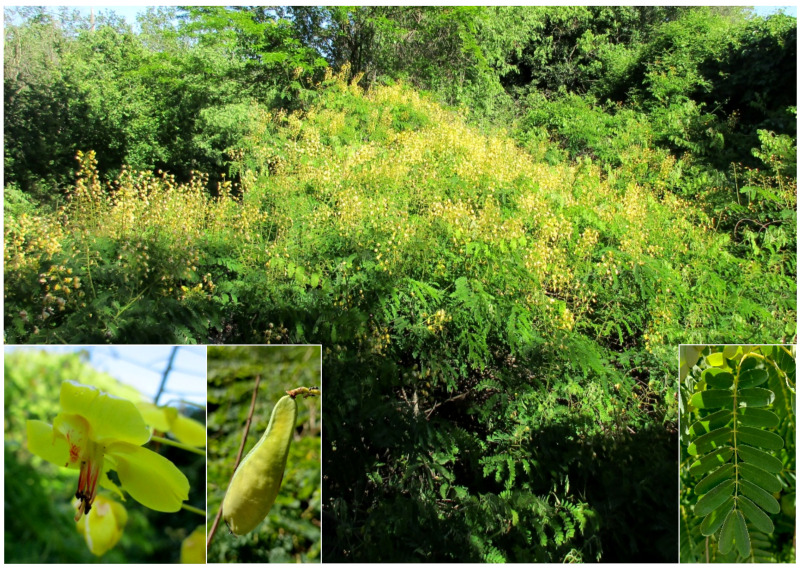
*Denisophytum bessac* on the western side of Caffarella valley; flower and fruit (bottom-left insets), pinna (bottom-right inset).

***Denisophytum bessac* (Choiv.) E.Gagnon & G.P.Lewis** (≡ *Caesalpinia bessac* Chiov.): a population of this species (monophytic shrub community; Figure 43) was first observed during the spring of 2016 on the west side of Caffarella valley. I initially identified this population as belonging to the genus *Caesalpinia* L., mainly based on the flower and leaf morphologies [56]. However, on the basis of a recent taxonomic work [57], a new classification of the *Caesalpinia* group was proposed, recognizing 26 genera (some newly described, other ones resurrected or redelimitated). By using the diagnostic key (genus rank) provided by Gagnon and collaborators [57], the identification of the Roman population (erect shrubs armed, with leaves bipinnate terminating with a pair of pinnae, flowers yellow, and fruits dehiscent and unarmed) was restricted to *Caesalpinia* or *Denisophytum* R. Vig. Note that these two genera are clearly separated from the molecular point of view (see Figure 3A in [57]) and the resurrection of *Denisophytum* is well supported. On the other hand, these two genera are morphologically similar, as highlighted by the authors (“no reliable diagnostic characters have been found to differentiate these two genera” [57] pag. 45). The only character considered to distinguish *Denisophytum* and *Caesalpinia* is the color of the flowers, which are yellow (sometimes with red markings on the standard, i.e., the median petal) in *Denisophytum* and orange, red, green, or white (rarely yellow or pink) in *Caesalpinia* ([57] pag. 27). Furthermore, on the basis of the detailed emended descriptions given, the two genera differ by the length of the fruits (pods), which are 18–49 mm long in *Denisophytum* ([57] pag. 45) and 34–120 mm long in *Cesalpinia* ([57] pag. 43). Flowers in the Roman population are all yellow, with minute red spots on the standard (Figure 43); pods (pers. obs.) are up to 50 mm long (never less than 40 mm). All things considered, I here identify the Roman population as a *Denisophytum* species.

The identification at species rank was quite difficult. Currently, *Denisophytum* comprises eight species, but unfortunately, no diagnostic key at species rank was provided by Gagnon and collaborators [57]. Furthermore, no further comprehensive key of *Denisophytum* taxa was found in the literature, and the descriptions of the taxa, when existing, are not very detailed. Hence, I decided to check the protologues of all eight of these species and examine all of the original material that I was able to trace. On the basis of this research (the complete work is still ongoing), I assembled the following data:

⮚ *D. bessac* (Choiv.) E. Gagnon & G.P. Lewis (≡ *Caesalpinia bessac* Chiov.): Chiovenda described this species (sub *Caesalpinia bessac*) in his *Flora Somala* [58], providing a detailed description that matches the plants found in Caffarella valley. The same author described also *C. eriantherum* Chiov. (see below) as morphologically similar to *C. bessac*. These two species differ from each other by the size of their leaflets, which are longer in *C. bessac* (basal leaflets 9–12 mm long vs. 4–5 mm long in *C. eriantherum*; distal leaflets 10–20 mm long vs. 7–8 mm long in *C. eriantherum*).

**Syntype found**: FI001388.

⮚ *D. buchii* (Urb.) E. Gagnon & G.P. Lewis (≡ *Denisophytum buchii* Urb.): this species displays the calyx as 7–8 mm long according to the protologue [59], whereas plants found in Caffarella have a calyx 3–5 mm long; furthermore, the leaflets are emarginate at the apex in *D. buchii* (“foliolis... apice emarginatis”), while leaflets in my specimens are obtuse to rounded.
⮚ *D. eriantherum* (Chiov.) E. Gagnon & G.P. Lewis (≡ *Caesalpinia eriantherum* Chiov.): on the basis of the original description by Chiovenda ([58] sub *Caesalpinia eriantherum*), plants found in Caffarella valley differ by both the size of leaflets [those basal 4–5 mm long (protologue) vs. 8–12 mm long (Caffarella plants); those distal 7–8 mm long (protologue) vs. 15–20 mm long (Caffarella plants) and the number of flowers per inflorescence, i.e., 10 (protologue) vs. up to 30 (Caffarella plants)]. Further, a var. *pubescens* (Brenan) E. Gagnon & G.P. Lewis (≡ *Caesalpinia erianthera* var. *pubescens* Brenan) is currently accepted, but it is characterized in having leaflets that are densely pubescent [60], whereas Roman plants have leaves that are glabrous.

**Syntypes found**: FI001390 (var. *eriantherum*), FI001392, and K000232357 (var. *pubescens*).

⮚ *D. madagascariense* R. Vig.: it is the only unarmed species in *Denisophytum* [57] pag. 45, while Caffarella plants have curved thorns along the shoot.

**Syntypes found**: P00131739.

⮚ *D. pauciflorum* (Griseb.) E. Gagnon & G.P. Lewis (≡ *Libidibia pauciflora* Griseb.): this species is different from all other species in having few flowers per raceme (“recemis simplicibus laxis paucifloris (v. pedunculis 1floris)" in the protologue [61]) and corolla slightly longer than the calyx (“calycis... corolla paullo superantibus”), whereas my specimens have many flowers per raceme (up to 30, never less than 20), and corollas are 5–7 times longer than the calyx.

**Syntypes found**: GH00065814, P02142660, P02142661, UC936921, US00382832, and YU001398;

⮚ *D. rosei* (Urb.) E. Gagnon & G.P. Lewis (≡ *Caesalpinia rosei* Urb.): it has leaflets glaucous in the abaxial surface (“foliole... in siccu supra obscure viridia, subtus valde pallida, glaucescentia” in the protologue [62]). Plants found in Rome show leaves green on both surfaces.

**Syntypes found**: NY00022764 and US00479309;

⮚ *D. sessilifolium* (S.Watson) E. Gagnon & G.P. Lewis (≡ *Caesalpinia sessilifolia* S.Watson): it is characterized in having sessile leaves, each one with two or three pairs of pinnae according to the protologue [63]. Caffarella plants have leaves that are petiolate with mostly five pairs of pinnae (rarely four).

**Syntypes found**: A00061947, AC00319854, BR0000005110933, CAS0001542, COL000092321, F0057403F, GH00059873, GOET004917, JE00004880, MICH1107159, MO125071, NA0026234, P02940720, PH00010119, RSA0003187, SI001822, US00344744, and US00345006.

⮚ *D. stuckertii* (Hassl.) E. Gagnon & G.P. Lewis (≡ *Caesalpinia stuckertii* Hassl.): the diagnosis and description given in the protologue [64] are congruent with the morphology of the plants found in Caffarella valley, except for the fruit, which was described as “oblongum basi et apice acutum”, whereas fruits in Caffarella’s plants have apexes that are obtuse-rounded (Figure 41); also, the illustration given by Gagnon and collaborators [57] pag. 44, Figure 12 displays a typical fruit of *D. stuckertii*, confirming that Roman plants cannot be assigned to this *Denisophytum* species. Further, Gagnon and collaborators’ illustration shows stipules that are clearly different from those of the Roman plants (foliaceous with two lobes vs. narrow, not lobed).

**Syntype found**: G00364837.

All things considered, the population found in Appia Antica Regional Park is identifiable as *Denisophytum bessac*, a species native to central Somalia and currently unknown elsewhere [65]. This record represents, therefore, not only the first one for Europe, but also the first discovery outside the native range of this Somalian species.

#### 4.4.2. New Records or Changes in Alien Status for Italy

**Figure 44:**
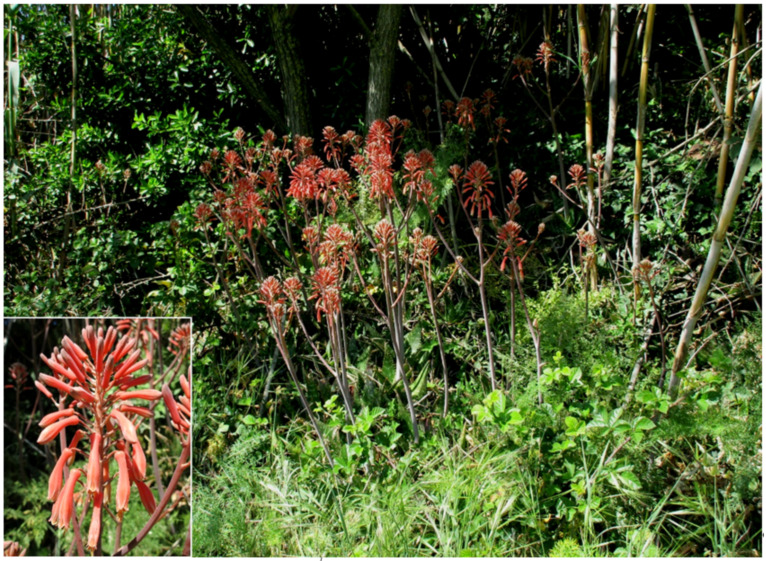
*Aloe maculata* All. subsp. *maculata* (Caffarella valley).

***Aloe maculata* All. subsp. *maculata***: this species, currently considered as casual in Italy [33], was recorded as casual in Latium for the first time in 2012 on the basis of a population found in Caffarella valley [66]. It is currently still considered as casual alien for the region [31,67]. After continuous monitoring over the years, it was observed that this population blooms regularly, is able to maintain itself by both vegetative and sexual reproduction (Figure 44), and has spread. I here consider *Aloe maculata* as naturalized in the Latium region, and this status is reported here for the first time at the national level.

 

**Figure 45:**
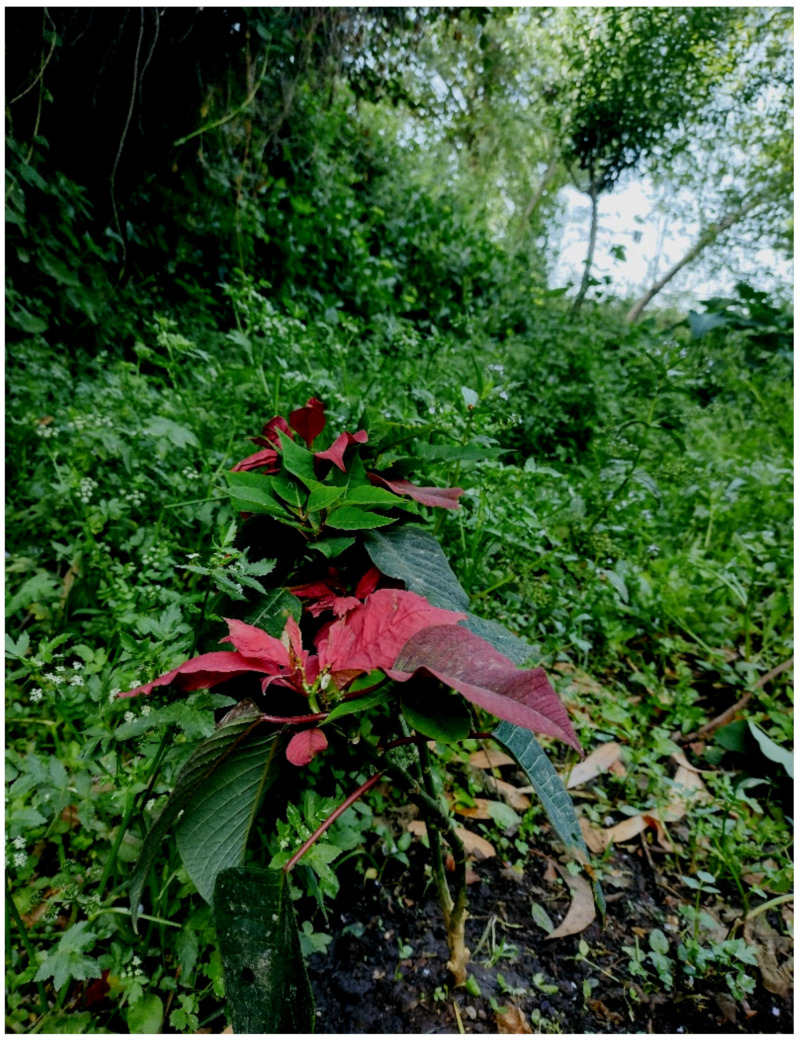
*Euphorbia pulcherrima* on riverbed of channel Acqua Mariana (Acquedotti locality).

***Euphorbia pulcherrima* Willd. ex Klotzsch**: this species was recorded for Italy only in the Campania region based on a single population found in Naples city [16,68]. Recently, *Euphoribia pulcherrima* was excluded from the region (and Italy) since the site in Naples was destroyed (a new garage was built) [69]. My discovery on a riverbed of channel Acqua Mariana (Acquedotti locality) represents the only Italian site in which the species (casual) certainty occurs (Figure 45).

 

**Figure 46:**
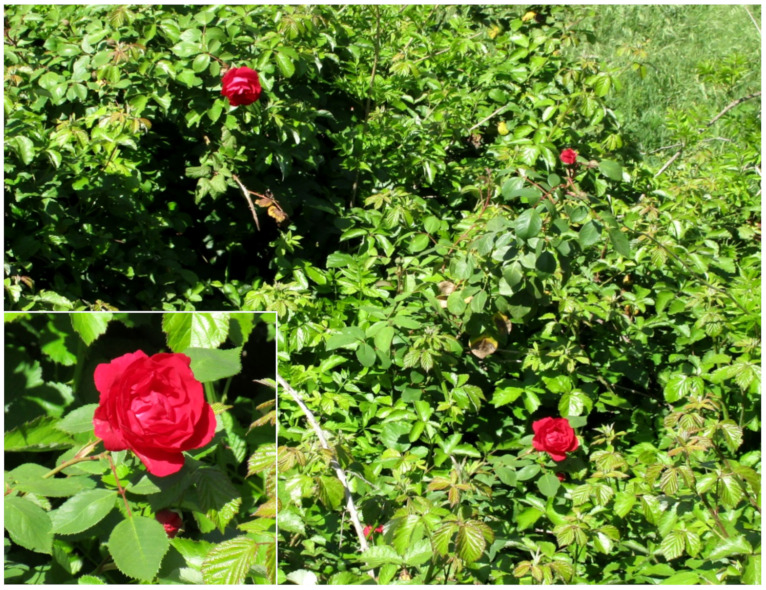
*Rosa chinensis* var. *semperflorens* in Caffarella valley.

***Rosa chinensis* Jacq. var. *semperflorens* (Curtis) Koehne**: I first observed, in spring 2015 in Caffarella valley, a small population of a rose cultivar growing in a shrub community dominated by *Rubus ulmifolius*. During the subsequent years, I again observed this population and found another one not far from the former (Figure 46). As a whole, these two populations seem to flourish occasionally in the park, and, therefore, their presence can be considered as casual.

Concerning the identification of this rose, I note first that it is a so-called modern rose having flowers with 17–20 petals [70]. On the basis of the treatment of the genus in *Flora of China* [71], as well as the *European Garden Flora* [72], plants found in Caffarella are identifiable as *Rosa chinensis* s.l. showing the following morphology: shrubs, leaves evergreen, each one with 3–5 leaflets, stipules adnate to petiole, hypanthium globose, flowers double, red-scarlet, usually solitary, up to 10 cm in diameter, not fragrant. Three varieties were recognized by Ku and Robertson [71], i.e., var. *chinensis*, var. *spontanea* (Rehder & E. H. Wilson) T. T. Yu & T. C. Ku, and var. *semprerflorens* (Curtis) Koehne. Var. *spontanea* differs from the other two by the flowers, which are single, whereas var. *chinensis* and var. *semperflorens* have flowers double or semi-double. Morphological differences between these two latter varieties refer to branches (robust in var. *chinensis* vs. slender in var. *semperflorens*), flowers (several and rarely solitary vs. solitary, rarely in fascicles of two or three), and petals (red, pink, or white vs. deep red or deep purple). The plants I found display slender branches and flowers solitary with petals deep red. Therefore, they are identifiable as var. *semperflorens* according to *Flora of China*.

According to Ku and Robertson [71], *Rosa chinensis* var. *semperflorens* has a cultivated origin and it is widely used in China. POWO [73] does not recognize infraspecific taxa of *Rosa chinensis*, recording it as native in South-Central China (it corresponds to var. *spontanea* in *Flora of China*, which is the only native variety from Guizhou, Hubei, and Sichuan) and as alien in other parts of Asia (India, Korea, Kazakhstan, Laos, Nepal, Pakistan, Uzbekistan, and Vietnam), North America (Alabama), Central America (Guatemala), Australia (Queensland and Western Australia), Europe (Belgium, Bulgaria, and Greece) and Oceania (Cook Islands and Guinea Islands); further (not reported in POWO), it was recorded as casual in Slovakia [74]. The occurrences in Europe are based on [75] for Belgium, [76] for Greece, and [77] for Bulgaria. Concerning Italy [33], *R. chinensis* is doubtfully recorded in Elba island (Tuscany, Central Italy) where the species “was possibly cultivated” [78]. So, my discovery in Rome represents the first certain record of *R. chinensis* s.l. in Italy.

#### 4.4.3. New Records or Confirmations for Latium

**Figure 47:**
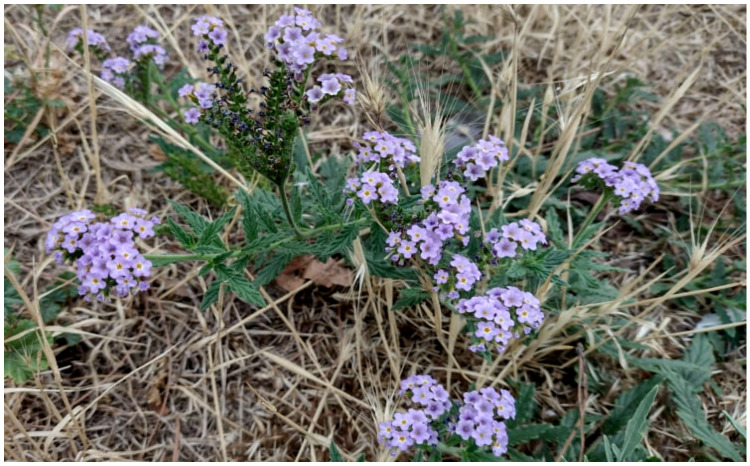
*Heliotropium amplexicaule* in the central reservation of Appia Nuova street.

***Heliotropium amplexicaule* Vahl.**: it is recorded in northern and peninsular Italy and Sicily [33]. Four sites are on the Italian peninsula, one in Tuscany (Pisa Province [79]) and three in Campania (Naples and Salerno Provinces [80]). For Latium, it was indicated as no longer recorded in Rome based on an old collection (April 1928, herbarium RO; [67,81]). I found in 2017 a population in the central reservation of Appia Nuova street (Figure 47), and observed it again in 2018 and 2022. The species is casual for Latium, representing the fifth record for the Italian peninsula.

**Figure 48:**
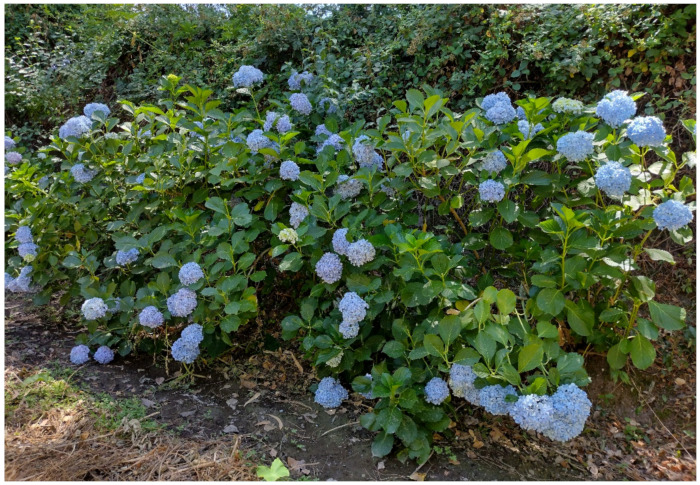
*Hydrangea macrophylla* along Acqua Mariana channel (Acquedotti locality).

***Hydrangea macrophylla* (Thunb.) Ser.**: this species was recorded in Italy [33] in the north (Lombardy, Trentino-Alto Adige, and Veneto regions, as casual), center (Tuscany region, as naturalized), and South (Campania region, as casual). The population found (first observation in 2015) grows along the channel Acqua Mariana (Acquedotti locality), blooms regularly, and sustains itself especially by vegetative reproduction; of note, the plants are regularly pruned (one or two times per year), but they rapidly re-grow and flowers appear after about 2 months (Figure 48). It can be considered a naturalized species and represents the first record for the Latium region.

*Hydrangea macrophylla* is a species native to Japan, and it is characterized in having high morphological variability [82,83], which led to the publication of several infraspecific names [84]; in fact, various authors accepted the recognition of infraspecific taxa (subspecies, varieties, and forms; see e.g., [82,85,86]), but the infraspecific variability is still incompletely known. Furthermore, a species related to *H. macrophylla*, i.e., *H. serrata* (Thunb.) Ser. ex DC., shows, in turn, an high phenotypic variability [87] and the relationship between these two species and their infraspecific taxa would need further study [82,83,84,85,86,87]. Lacking final conclusions about this group, I here prefer to avoid the use of infraspecific taxa and consider *H. macrophylla* as separate from *H. serrata*, according to POWO [84].

**Figure 49:**
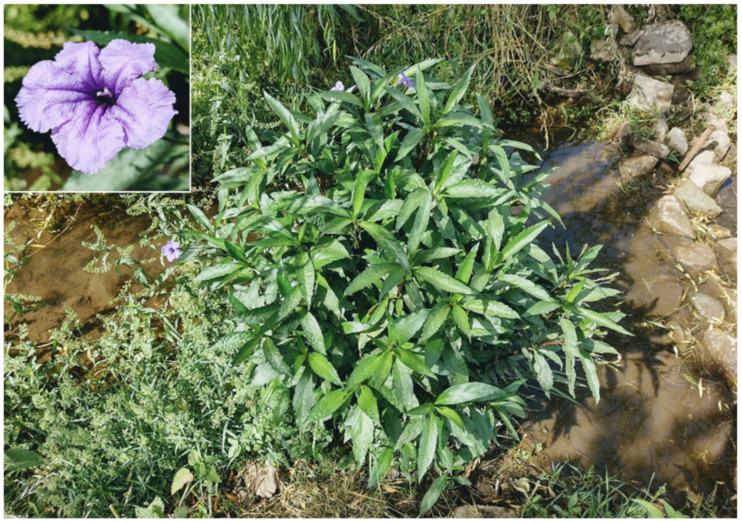
*Ruellia simplex* along channel Acqua Mariana (Acquedotti locality).

***Ruellia simplex* C. Wright**: this species is currently known in Italy as casual and recorded only in two regions, i.e., Apulia (southern italian peninsula) and on the island of Sardinia [33]. These findings refer to single localities, one per region, i.e., Otranto (Lecce Province) for Apulia [88] and Serramanna (Medio Campidano Province) for Sardinia [89]. The population found in Appia Antica Regional Park (Acquedotti locality, along the channel Acqua Mariana; Figure 49) represents the first record for the Latium region and the third one at the national level. The few individuals found were first observed in 2020, and they do not seem to be able to spread. So, *Ruellia simplex* is here considered as casual for Latium.

**Figure 50:**
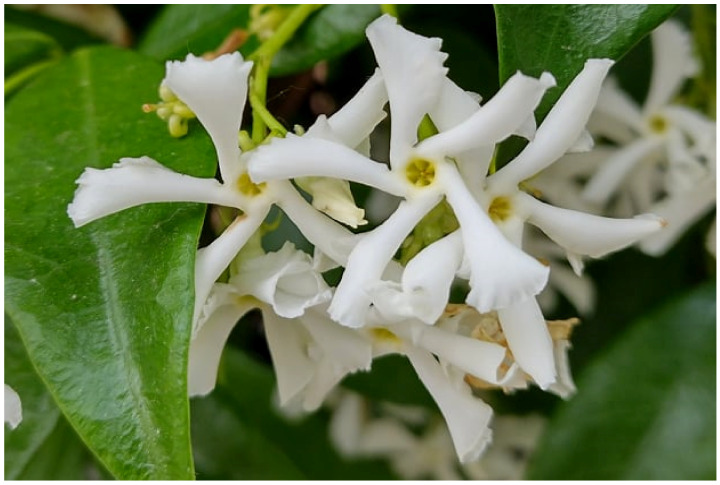
*Trachelospermum jasminoides* on cliff along Acqua Mariana channel (Acquedotti locality).

***Trachelospermum jasminoides*(Lindl.) Lem.** (≡ *Rhynchospermum jasminoides* Lindl.): this species is recorded in Italy as casual and only in two regions [33], i.e., Lombardy (just one site in Mantova Province; [90]) and Sardinia [just one site in Cagliari Province [91]). A small population was found in the Acquedotty locality on the cliff of channel *Acqua Mariana* (Figure 50), and it represents the first record for the Latium region and peninsular Italy (casual species) and the third one for Italy.

#### 4.4.4. Changes or Confirmation of Alien Status for Latium

**Figure 51:**
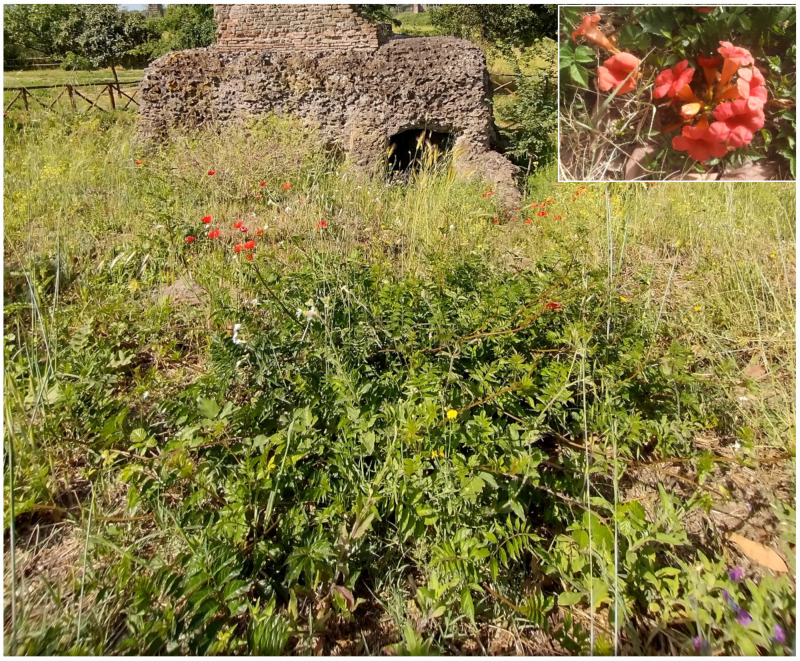
*Campsis radicans* in Acquedotti locality.

***Campsis radicans* (L.) Bureau**: this species was reported as casual alien for Latium in *Flora of Italy* [33], whereas in volume no. 1 of *Atlante della flora vascolare del Lazio* [67], it was considered as naturalized. Actually, the first indication of naturalization of *Campsis radicans* in Latium was in 2014 [66] on the basis of a population found in Caffarella valley. Of note, the *Flora of Rome* [9] does not list this species. I here confirm the occurrence and naturalization of *Campsis radicans* in Appia Antica Regional Park in Caffarella locality. Furthermore, a new population was recently found in the Acquedotti locality of the park (Figure 51).

**Figure 52:**
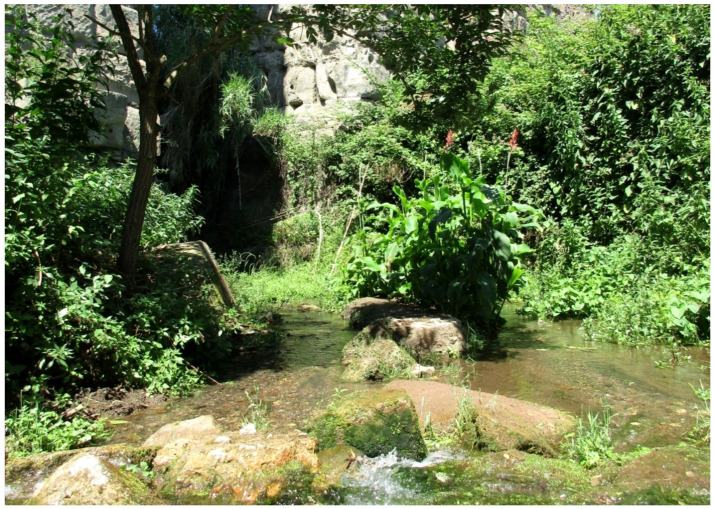
*Canna indica* along channel Acqua Mariana, near Claudio’s aqueduct (Acquedotti locality).

***Canna indica* L.**: this species is currently considered as casual alien in the Latium region [33,67]. I found many populations of *Canna indica*, especially in the northern sector of the park (Caffarella valley and Acquedotti locality; Figure 52) where, during the years, the number of individuals increased, thus showing that the populations are able to spread. All of the plants found bloom regularly. *C. indica* is here considered as naturalized in Latium.

 

**Figure 53:**
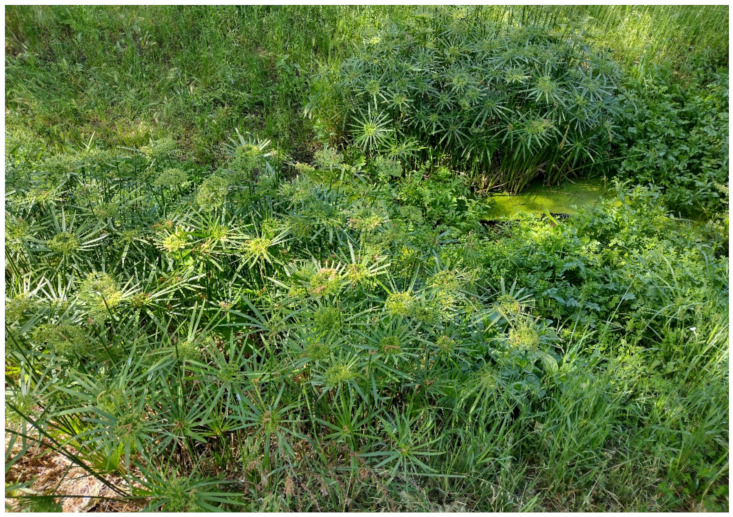
*Cyperus alternifolius* subsp. *flabelliforme* along channel Acqua Mariana as part of holophyte community of Phragmito-Magnocaricetea (Acquedotti locality).

***Cyperus alternifolius* L. subsp. *flabelliforme* Kük.**: this taxon was considered as a casual alien in Latium [33,67], and also indicated for Rome (sub *Cyperus involucratus* Rottb.). In Appia Antica Regional Park there are many individuals occurring especially in the northern sector (Tor Marancia, Caffarella, and Acquedotti localities), which have highly increased in number over the years. All of the plants found bloom regularly and the populations have spread (Figure 53). *C. alternifolius* subsp. *flabelliforme* is a naturalized species throughout the whole study area.

**Figure 54:**
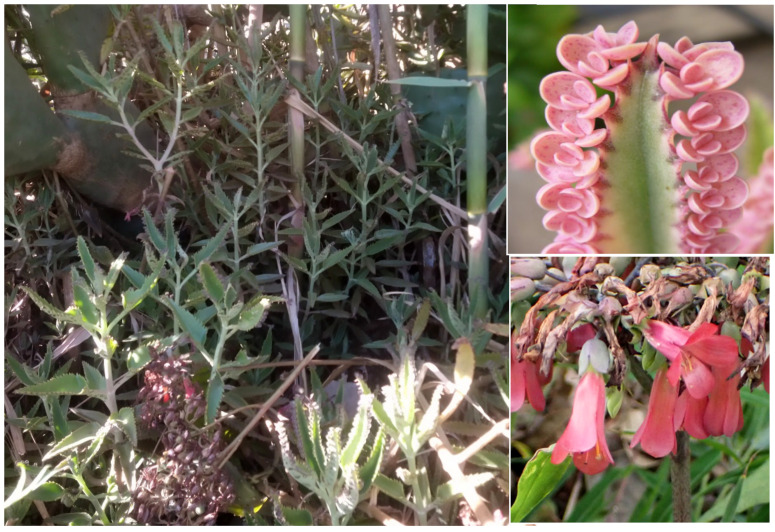
*Kalanchoe daigremontiana* in *Arundo donax* community (Caffarella valley).

***Kalanchoe daigremontiana* Raym.**: species reported as casual for Latium [33,67], but not cited in *Flora of Rome* [9]. The populations found in Appia Antica Regional Park are not only able to sustain themselves, but they have spread (Figure 54). *Kalanchoe daigremontiana* is a naturalized non-native species in Latium.

**Figure 55:**
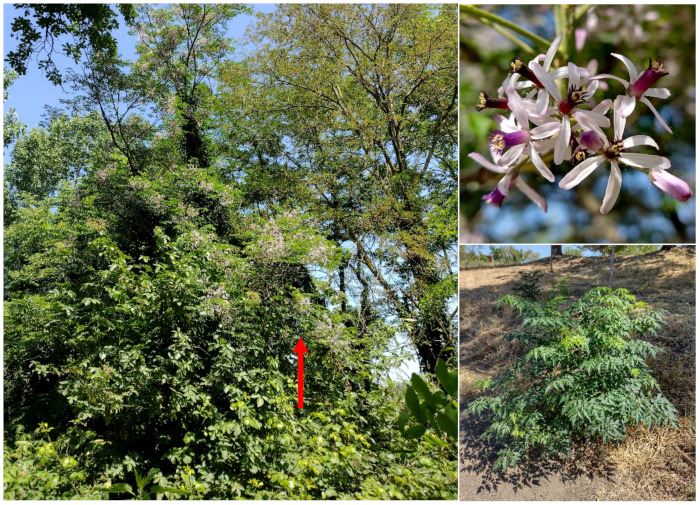
*Melia azedarach* (red arrow) in Caffarella valley (left and top-right photos); young individual in Acquedotti locality (bottom-right photo).

***Melia azedarach* L.**: this species was indicated as doubtfully spontaneous in *Atlante della flora vascolare del Lazio* [67], whereas it is casual for the region in Italian and Roman floras [9,33]. I here confirm the occurrence of *Melia azedarach* as casual in Latium, having found individuals in shrubs and forest communities and along paths (Figure 55).

**Figure 56:**
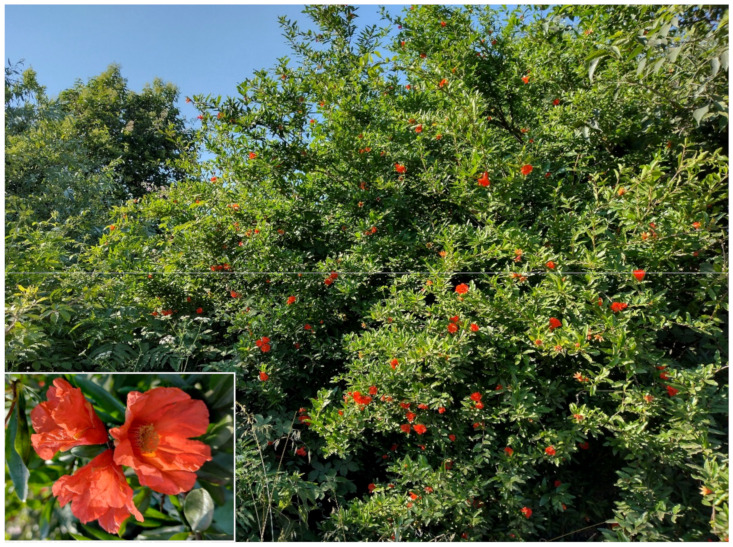
*Punica granatum* in Caffarella valley.

***Punica granatum* L.**: it is currently considered as casual in Latium [33,67] and Rome [9], whereas in *Flora vascolare del Lazio* by Anzalone and collaborators [92], *Punica granatum* is reported as naturalized, with various localities listed (including Rome). In Caffarella valley, I traced a population along a channel consisting of well-developed individuals (2–5 m tall, with truck of 15–20 cm in diameter) that have bloomed and fruited regularly for more than 10 years (Figure 56). Further scattered individuals were observed in Caffarella and Acquedotti localities. The species is naturalized in the park.

#### 4.4.5. New Records or Confirmations for Rome

**Figure 57:**
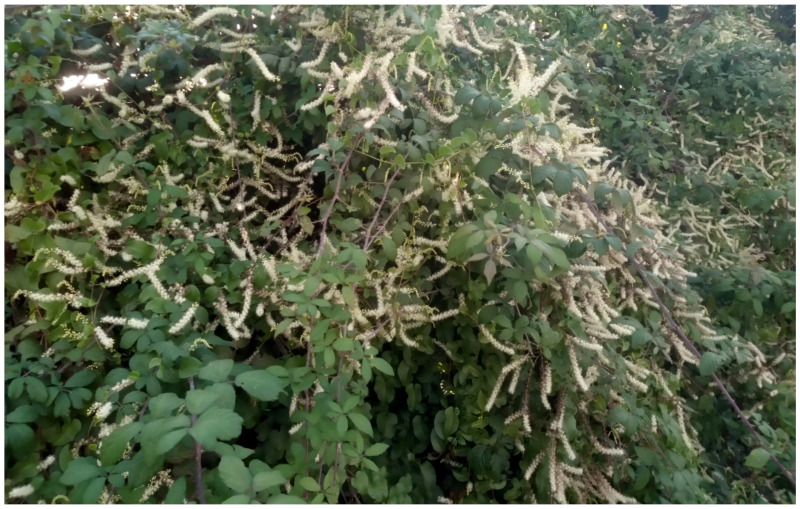
*Anreedera cordifolia* on *Rubus ulmifolium* dominated community (Acquedotti locality).

***Anreedera cordifolia* (Ten.) Steenis**: this species was not listed in the *Flora of Rome* [9], whereas in *Atlante della flora vascolare del Lazio* [67] it was indicated generically for “Roma città” (= Rome city). Two populations growing in a *Rubus ulmifolium* dominated community (Caffarella valley and Acquedotti locality; Figure 57) were found some years ago, and they appear to be well-established; the species is naturalized. This discovery confirms the occurrence of *Anreedera cordifolia* in Rome.

**Figure 58:**
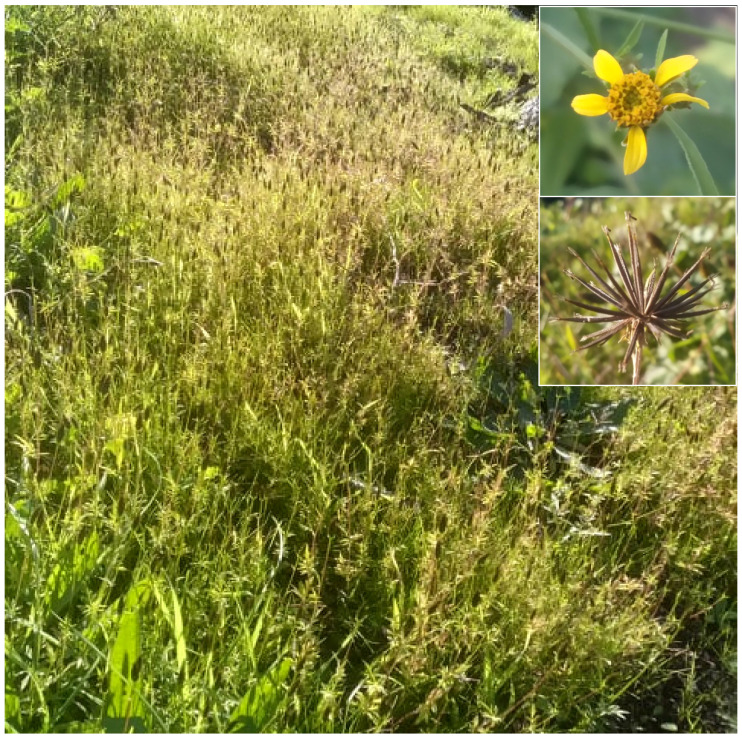
*Bidenssubalternans* in Acquedotti locality.

***Bidens subalternans*DC.**: this species was not listed in either the *Flora of Lazio* by Anzalone and collaborators [92] or the *Flora of Rome* [9], whereas the more recent *Atlante della flora vascolare del Lazio* [67] indicates *Bidens subalternans* DC. as casual in Tivoli and Genzano cities and the Aurunci Mountains. Recently, the species was considered as naturalized in Latium in Frosinone and Rome Provinces [93]. In any case, the species was not reported for Rome city. My discovery (a dense population growing on a little bridge of channel *Acqua Mariana*) represents, therefore, the first one for the city, and it can be considered as casual for the moment (Figure 58).

**Figure 59:**
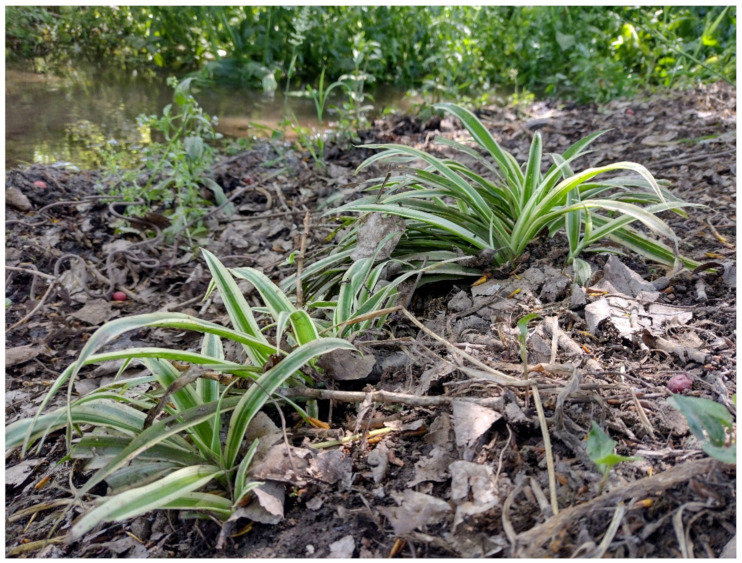
*Chlorophytum comosum* along channel Acqua Mariana (Acquedotti locality).

***Chlorophytum comosum* (Thumbs.) Jacques**: this species was recently recorded for the first time in Latium (Viterbo Province, Orte Scalo locality) as casual [91]. My discovery on the banks of channel *Acqua Mariana* (Acquedotti locality; Figure 59) represents the first one for Rome Province and the second at the regional level. I first observed the population in 2017, and it persisted during the years (last observation in June 2022), but the population does not spread and is, therefore, considered as casual.

**Figure 60:**
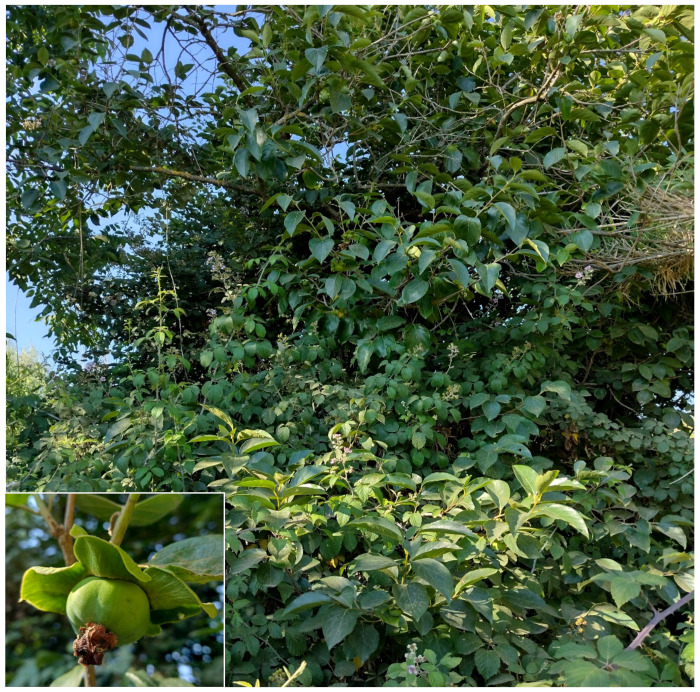
*Diospyrus kaki* along a channel in Caffarella valley.

***Diospyrus kaki* L.**: this species was reported for Latium in both *Flora of Italy* [33] and, along the river Tevere, in *Flora of Lazio* [92]; contrarily, it was not listed either in *Flora of Rome* [9] or in *Atlante della flora vascolare del Lazio* [67]. Some individuals, which bloom and fruit regularly, were traced in Caffarella valley (Figure 60), thus confirming the occurrence of *Diospyrus kaki* in the city.

 

**Figure 61:**
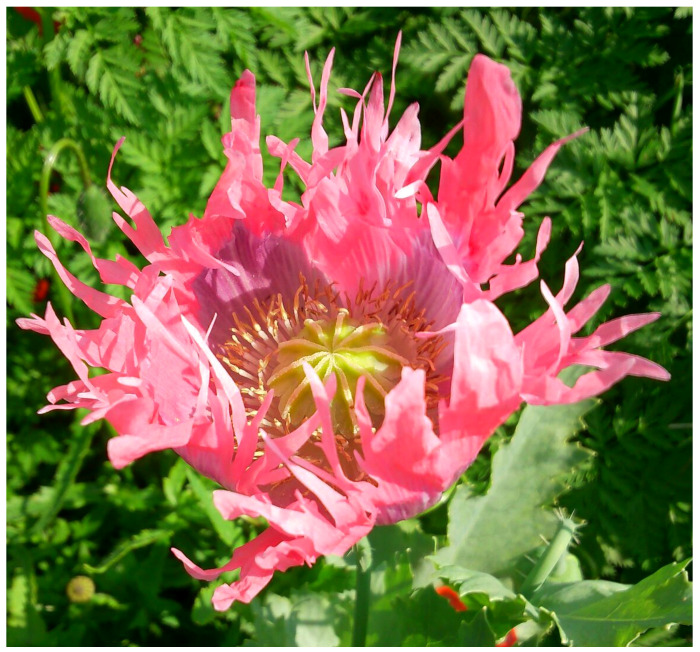
*Papaver somniferum* in Caffarella valley.

***Papaver somniferum* L.**: this species was listed in the *Flora of Rome* as “doubtfully alien” [9], but not later reported for the Italian capital in *Atlante della flora vascolare del Lazio* [67]. Scattered individulas were found in Caffarella valley, thus confirming the occurrence of *P. somnifermum* in Rome (Figure 61).

 

**Figure 62:**
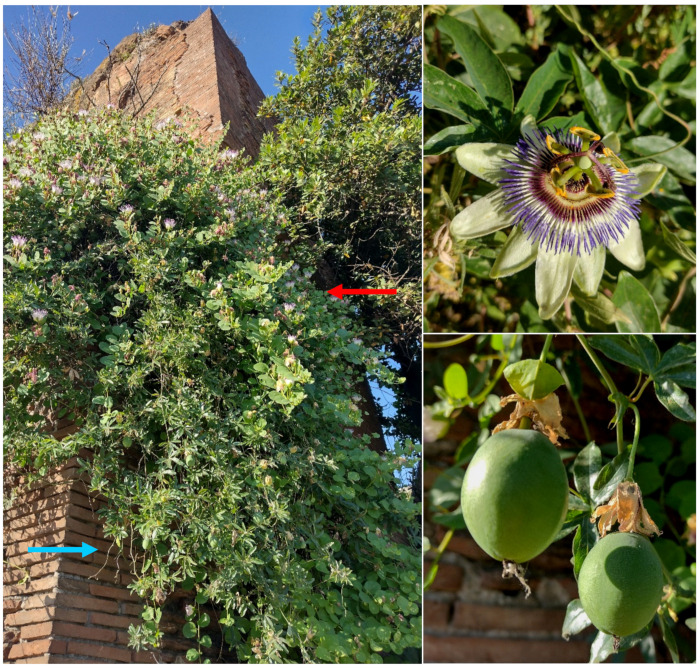
*Passiflora caerulea* (blue arrow) associated with *Capparis orientalis* (red arrow) on the ancient Roman Claudio’s aqueduct.

***Passiflora caerulea* L.**: this species was reported in the *Flora of Rome* as casual [9], but not later indicated for the city in *Atlante della flora vascolare del Lazio* [67]. I found various individuals growing on the Claudio’s aqueduct (Figure 62), thus confirming the occurrence (as casual) of *Passiflora caerulea* in Rome.

 

**Figure 63:**
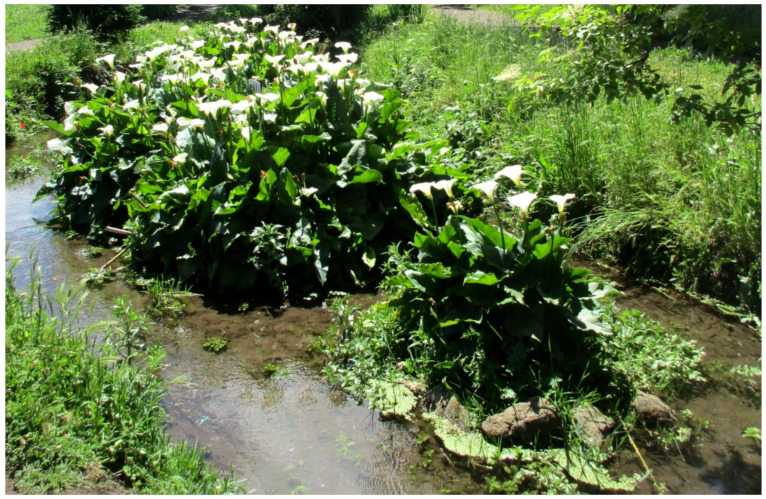
*Zantedeschia aetiopica* along channel Acqua Mariana (Acquedotti locality).

***Zantedeschia aetiopica* (L.) Spreng.**: this African species is not currently reported for Rome [9,67]. In Appia Antica Regional Park, individuals were found along channels in Caffarella valley and, especially, in the Acquedotty locality, where the species is able to spread along the channel Acqua Mariana (Figure 63). I hereby consider this alien as naturalized.

#### 4.4.6. Species No Longer Recorded

Literature analysis and herbaria investigations allow the verification of nine species collected in the past in Appia Antica Regional Park, but no longer recorded (see Appendix B). I never found any population of these species during the field surveys. Seven species are native, and two (*Ehrhata erecta* Lam. and *Tarenaya spinosa* (Jacq.) Raf.) are allochthonous (native, respectively, to South America and Africa).

 

***Astragalus glycyphyllos* L.**: this species was recorded in Caffarella valley in the *Centuriae XII* of *Florae Romanae Prodromus* [19] pag. 240 and not listed among the flora of Caffarella valley [25]. No specimen collected in the territory of Appia Antica Regional Park was traced. *A. glycyphyllos* is not a common species in the territory of Rome Municipality, although it is very common in the Latium region as a whole [92]. Although the study area was investigated in depth for more than 10 years through field surveys, further field investigations are needed to verify its occurrence in the park.

 

***Catabrosa aquatica* (L.) P.Beauv.**: this species was recorded in Caffarella valley in *Egeria nymphaeum* (“nella Grotta della Ninfa Egeria” = in the cave of Nymph Egeria) in the *Centuriae XII* of *Florae Romanae Prodromus* sub *Aira aquatica* L., [19] pag. 38. *C. aquatica* was not listed in the flora of either Caffarella valley [25] or Rome city, in the *Flora of Lazio* [92]. No specimen collected in Caffarella valley was traced. However, I traced one specimen (included in Montelucci’s Herbarium at RO; collection number 5621) bearing a plant collected by G. Montelucci in April, 20 (year 1944) at “Via Appia Nuova, lungo fossetto tra Ciampino e S. Maria delle Mole. Luoghi aquitrinosi” (= Appia Nuova street, along channel between Ciampino and S. Maria delle Mole. swampy sites). The area around these two localities (Ciampino and S. Maria delle Mole) would represent the southernmost part of Appia Antica Regional Park, at least partially (the part on the west side of Appia Nuova street). However, the landscape configuration has deeply changed in this sector of the park over time, especially with new buildings. Therefore, the “swampy sites” cited by G. Montelucci in the label, which could be at that time, were probably destroyed; I will verify the absence of this type of habitat during field trips. The species is potentially extinct in the territory of the park.

 

**Figure 64:**
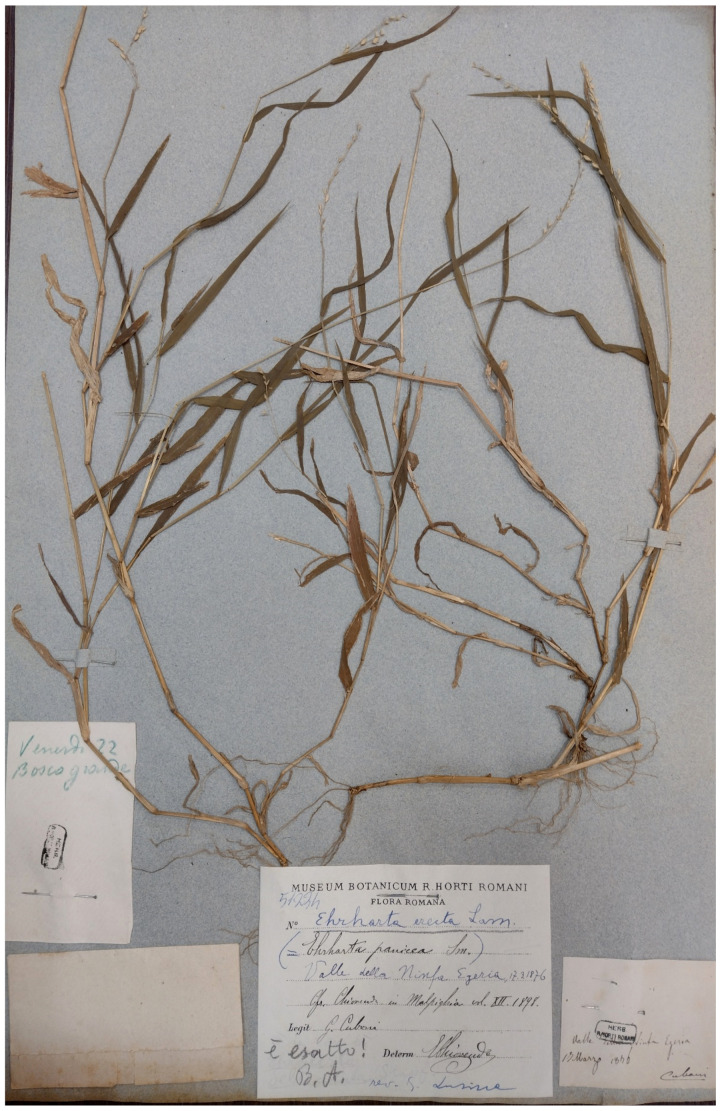
Specimen of *Ehrharta erecta* collected in Caffarella valley on 17 March 1876 (RO-Herbarium Romano no. 51234).

***Ehrharta erecta* Lam.**: this species is listed in the flora of Caffarella valley [25] as no longer recorded. This indication was based on a specimen (deposited at RO; Figure 64) collected by G. G. B. Cuboni on 17 March 1876 at “Valle della Ninfa Egeria” (= Valley of Ninfa Egeria). *E. erecta* is an alien species for Italy [31], and its ability to reproduce is probably very low in Latium; this could be the reason why this species was no longer recorded.

 

**Figure 65:**
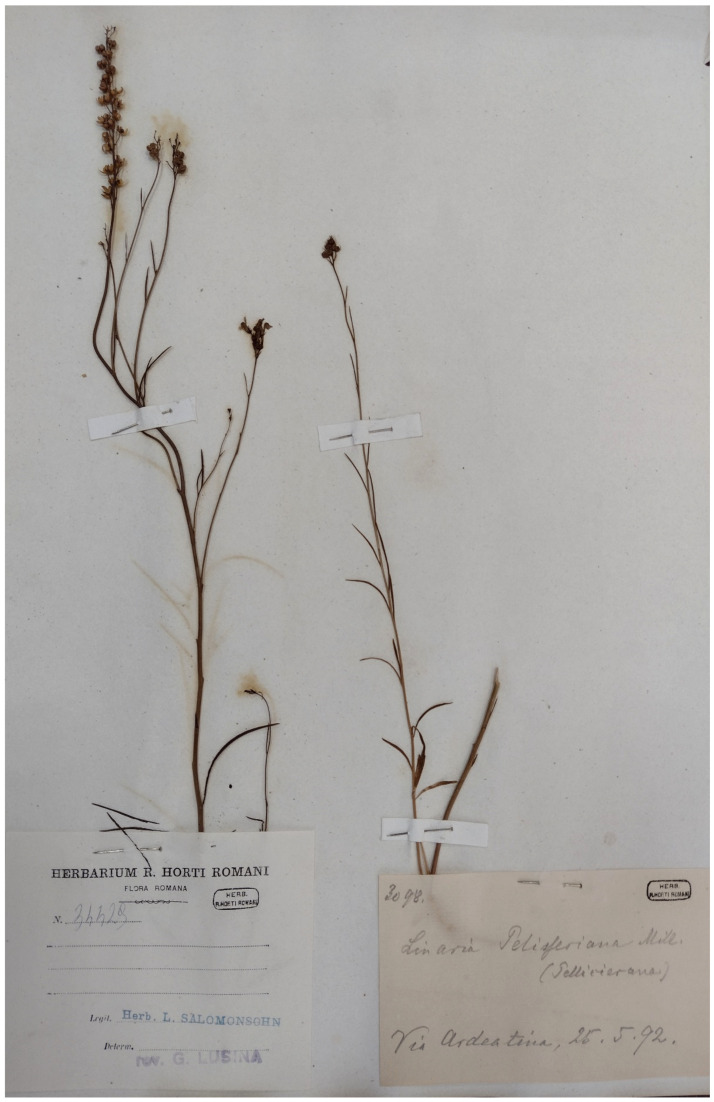
Specimen of *Linaria pelisseriana* collected along Ardeatina street on 15 May 1892 (RO-Herbarium Romano no. 34429).

***Linaria pelisseriana* (L.) Mill.**: this species was recorded in Caffarella valley in the *Centuriae XII* of *Florae Romanae Prodromus* [19] pag. 203; it was not cited in the flora of Caffarella valley [25]. Only one specimen (deposited at RO) collected in the territory of Appia Antica Regional Park (Ardeatina street on 15 May 1892) was traced (Figure 65). *L. pelisseriana* is a therophyte with a very short life cycle (flowering time March–April [28]), being also very rare in the territory of Rome Municipality. However, it is common in Latium [92]. Although the study area was investigated in depth for more than 10 years through field surveys, I cannot consider this species as extinct (or potentially so), and I think that further field trips are necessary to verify its presence in the park.

 

**Figure 66:**
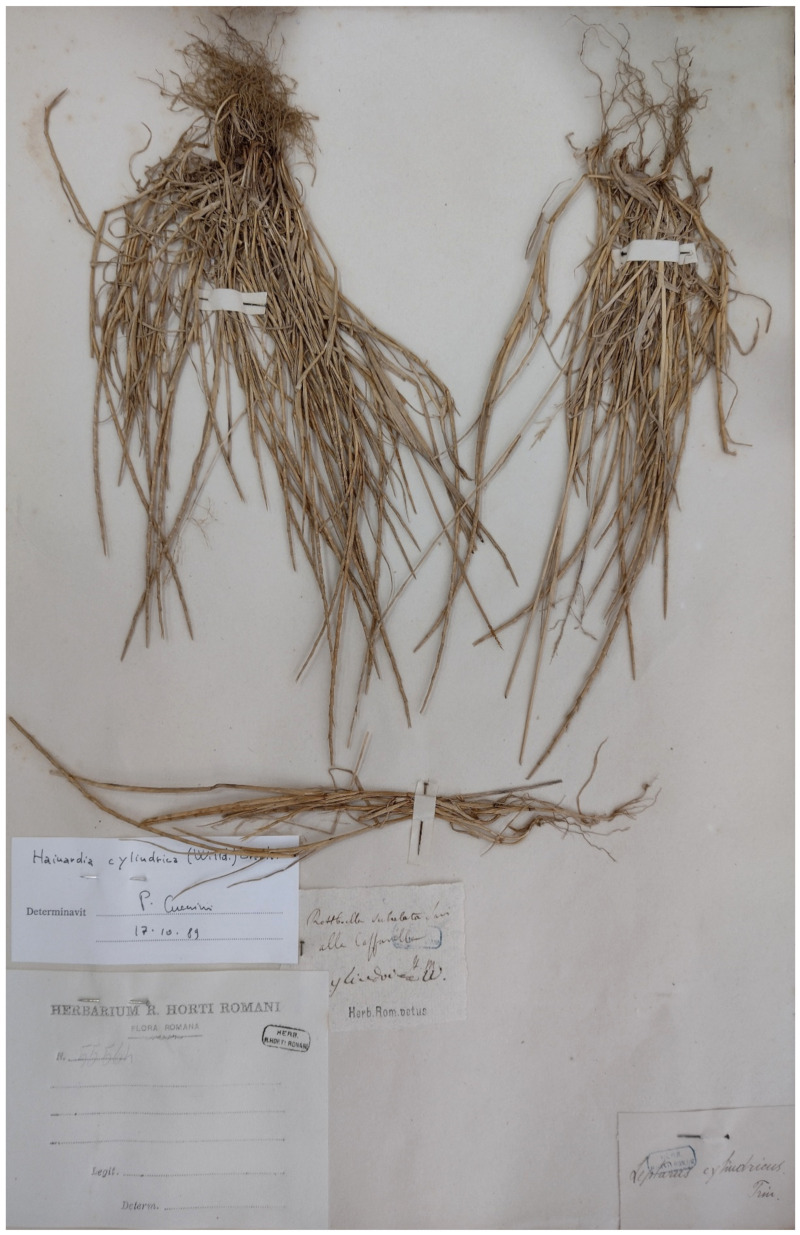
Specimen of *Parapholis cylindrica* collected in Caffarella valley in the 19th century (RO-Herbarium Romano no. 55564).

***Parapholis cylindrica* (Willd.) Romero Zarco**: this species was recorded in Caffarella valley (sub *Rottboellia subulata* Savi) in the *Centuriae XII* of *Florae Romanae Prodromus* ([19], pag. 62]), but not reported in the flora of Caffarella valley [25] and Rome [9], or Rome city in the *Flora of Lazio* [92]. Only one specimen (deposited at RO) collected in the territory of Appia Antica Regional Park (Caffarella on XIX century) was traced (Figure 66). *P. cylindrica* is a species growing mainly along Mediterranean coasts (rarely on inlands) on subsaline and clay soils [28]. Subsaline soils are not present in the territory of the park to date, and it is possible that the habitat was lost or destroyed. The species, which is considered rare in Italy as a whole [28], is potentially extinct in Appia Antica Regional Park.

 

**Figure 67:**
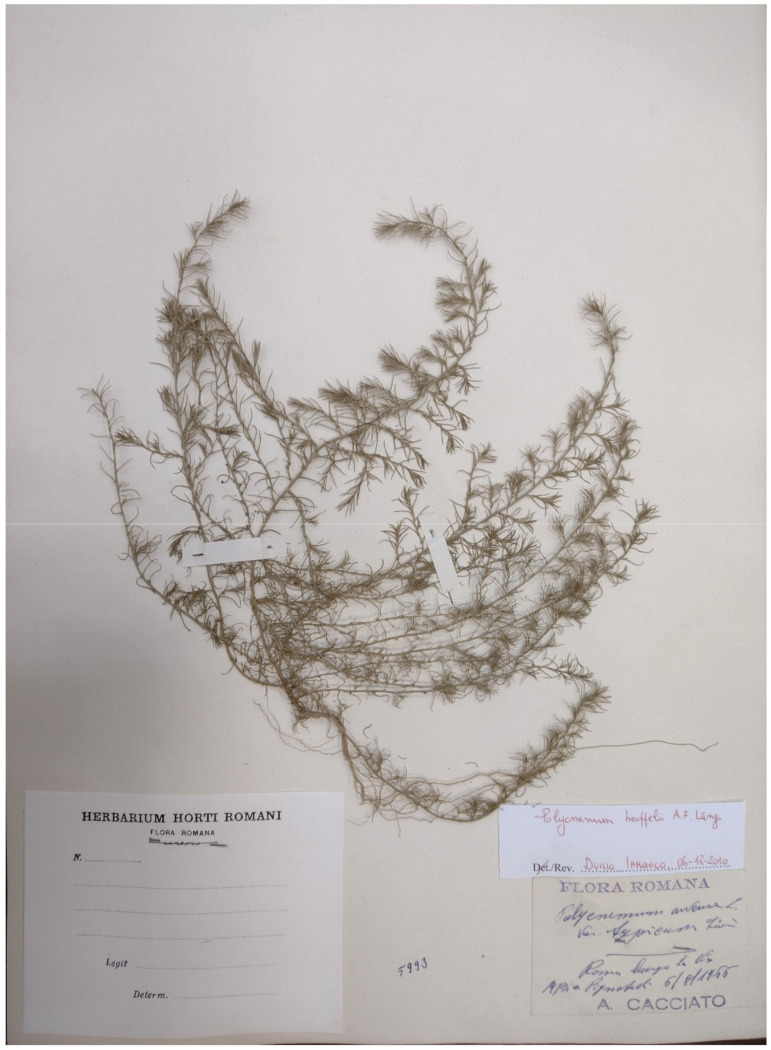
Specimen of *Polycnemum heuffelii* collected along Appia Pignatelli street in august 1966 (RO-Herbarium Romano no. 5993).

***Polycnemum heuffelii* Láng**: the occurrence in Italy of this European species (distributed from Poland to Greece) is based on just one specimen (deposited at RO; Figure 67), collected by A. Cacciato along Appia Pignatelli street on 6 August 1966 [94]. The Flora of Italy [33] report this species as cryptogenic. The habitat in which this species was discovered (roadsides) was exposed to various types of human activities during the time; this factor could have caused the extinction of the Roman population.

 

**Figure 68:**
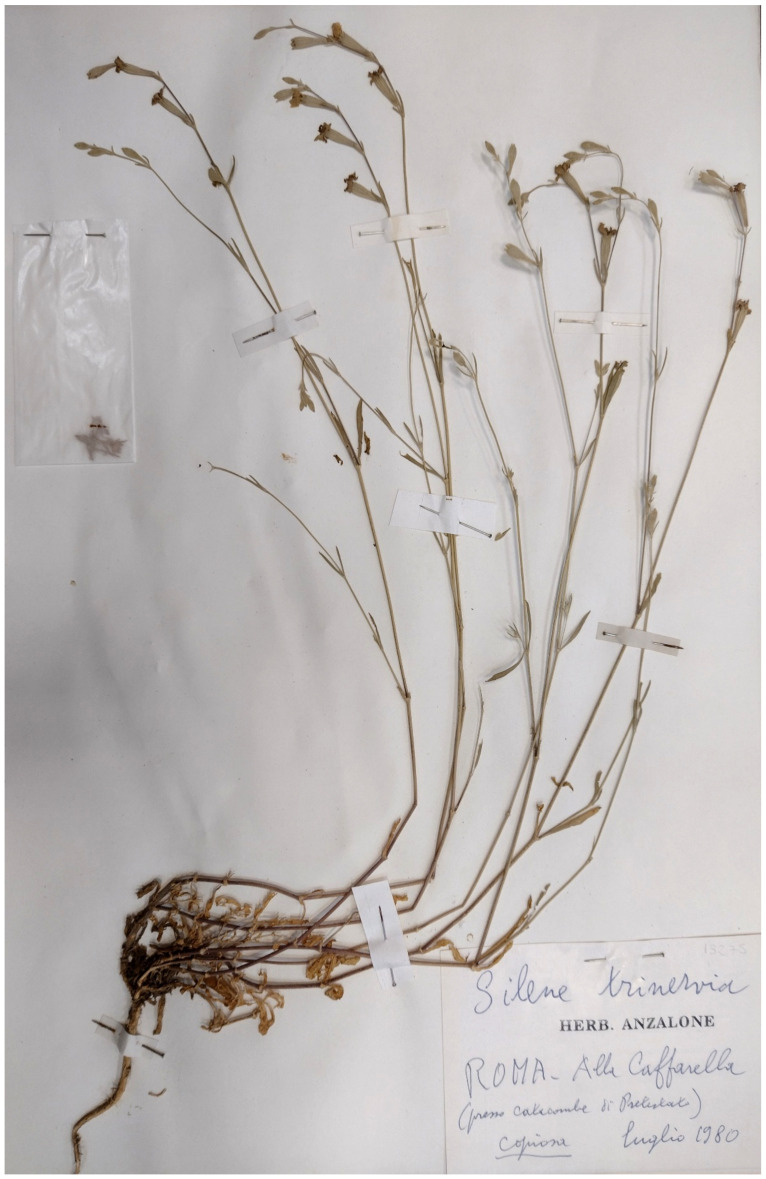
Specimen of *Silene gallinyi* (sub *S. trinervia*) collected in Caffarella valley in July 1980 (RO-Herbarium Anzalone no. 13276).

***Silene gallinyi* Rchb.**: this species was recorded in Caffarella valley (sub *Silene trinervia* Sebast. & Mauri) in *Florae Romanae Prodromus* [19]. *S. trinervia* was there firstly described with *Locus classicus* including the locality Caffarella (“Alla Caffarella presso il Fonte di Egeria...” = At Caffarella near Egeria’s spring). This *Silene* species was not listed in the flora of Caffarella valley [25]. After Herbaria checking (including RO, where original collections of A. Sebastiani and E. Mauri are currently deposited [95,96]), I verified that the last collections of this species were dated June–July 1980 (three specimens in Anzolone’s Herbarium at RO; Figure 68). *S. gallinyi* is a species that can be easily distinguished from the other *Silene* taxa occurring in the territory of the park [*S. bellidifolia* Jacq., *S. conica* L., *S. gallica* L., *S. italica* (L.) Pers. subsp. *italica*, *S. latifolia* Poir., *S. nocturna* L., *S. pendula* L., *S. vulgaris* (Moench) Garcke subsp. *tenoreana* (Colla) Soldano & F.Conti, *S. vulgaris* (Moench.) Garcke subsp. *vulgaris*]. Despite this, anf the more than 10 years of field surveys, some carried out specifically to search for *S. gallinyi*, I did not yield positive results. My opinion is that this species is extinct throughout the park.

 

**Figure 69:**
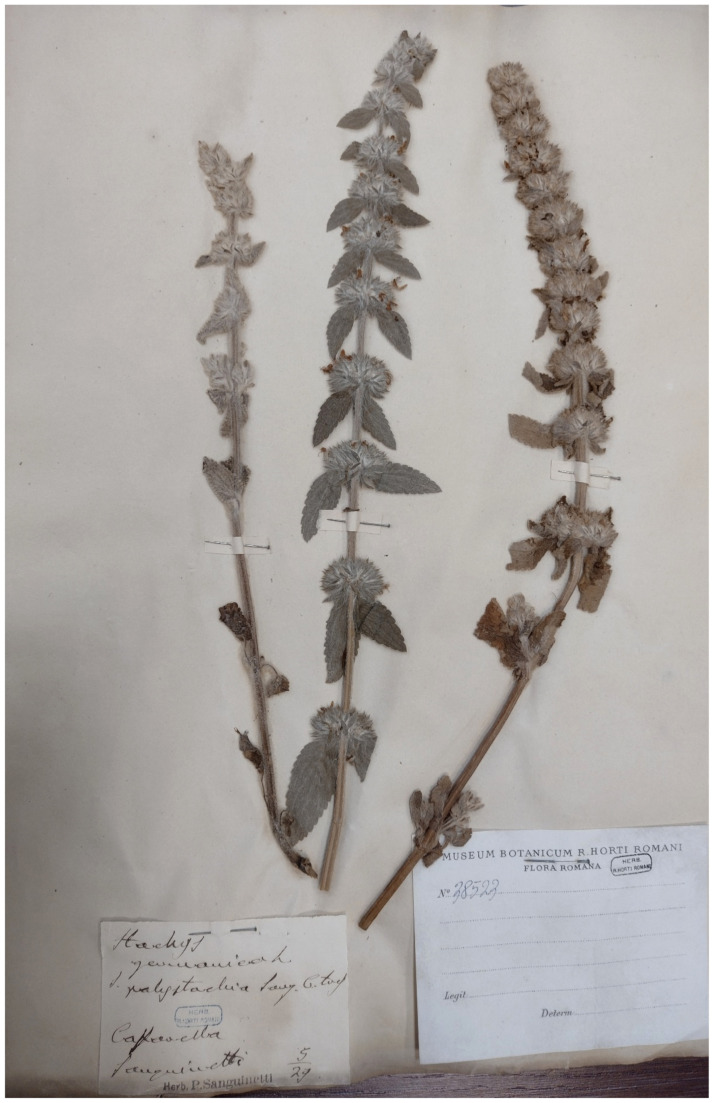
Specimen of *Stachys germanica* L. subsp. *germanica* collected in Caffarella valley in May 1829 (RO-Herbarium Romano no. 38523).

***Stachys germanica* L. subsp. *germanica***: this species was recorded in Caffarella valley in *Egeria nymphaeum* (“presso la Grotta di Egeria” = near the cave of Egeria) in the *Centuriae XII* of *Florae Romanae Prodromus* ([19] pag. 194). *S. germanica* s.str. was not listed in the flora of Caffarella valley [25]. Five specimens (at RO) collected in the territory of Appia Antica Regional Park [Caffarella valley (May 1829) and in uncultivated lands near Cecilia Metella sepulcher (3 June 1922)] were traced (Figure 69). *S. germanica* subsp. *germanica* is not a common species in the territory of Rome Municipality, although it is very common in the Latium region as a whole [92]. Plants of this species are easy to see in field due both to their size (30 to 60 cm on average) and their hairiness (densely white-woolly). Despite these facts, I never found it during >10 years of field surveys. The species is potentially extinct in the territory of the park.

 

**Figure 70:**
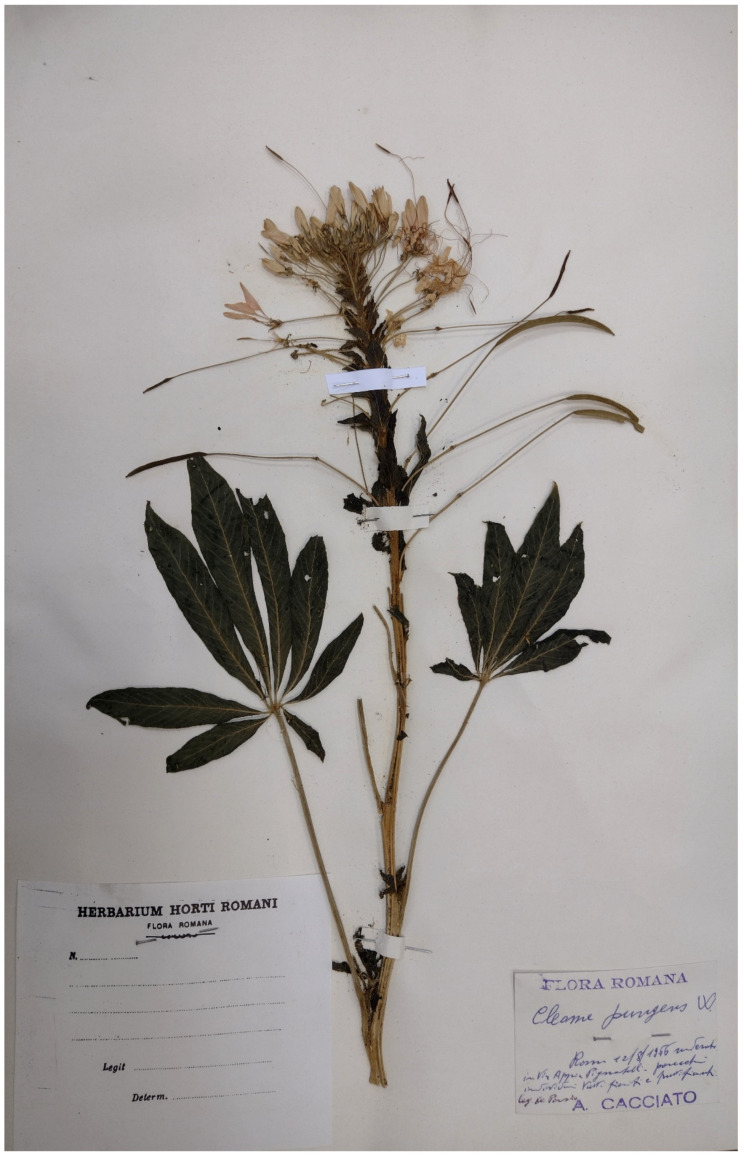
Specimen of *Tarenaya spinosa* collected along Appia Pignatelli street in august 1965 (RO-Herbarium Romano s.n.).

***Tarenaya spinosa* (Jacq.) Raf.**: this species is recorded in the Latium region based on a specimen (deposited at RO; Figure 70) collected by A. Cacciato along Appia Pignatelli street in 1966 [78,92]. It was not reported in the *Flora fo Rome* [9]. I never found any individual of *Tarenaya spinosa* during field surveys. *T. spinosa* is no longer found for two possible reasons: a low ability to reproduce and modification of its habitat (roadsides) by humans.

#### 4.4.7. Species Having Loci Classici and/or Nomenclatural Types Collected in Appia Antica Regional Park

**Figure 71:**
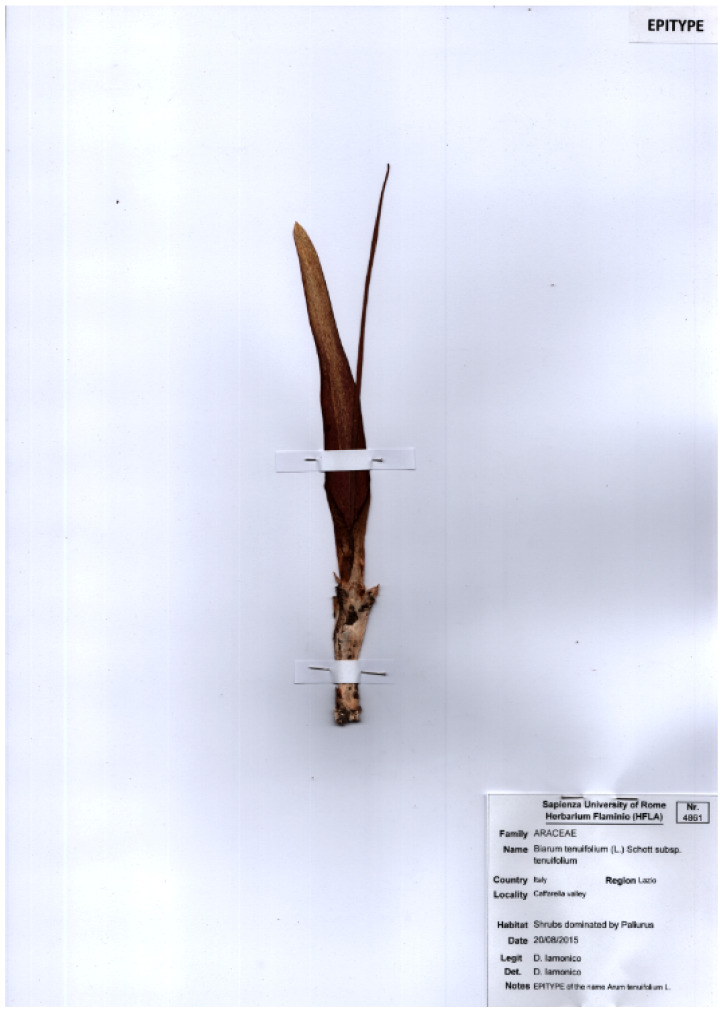
Epitype of *Biarum tenuifolium* subsp. *tenuifolium* (HFLA no. 4861).

***Biarum tenuifolium* (L.) Schott subsp. *tenuifolium***: the basionym *Arum tenuifolium* L. was described by Linnaeus in the first edition of his *Species Plantarum* [97], where the provenance “*Habitat in* Romae” was reported. The Linnaean name was recently lectotypified by Iamonico [98] on a specimen preserved at BM (barcode BM000647349); further, an epitype collected in the Caffarella valley (20 August 2015) was designated (the specimen kept at HFLA; Figure 71) to serve as an interpretative type according to the current concept in *Arum* (see e.g., [99]).

**Figure 72:**
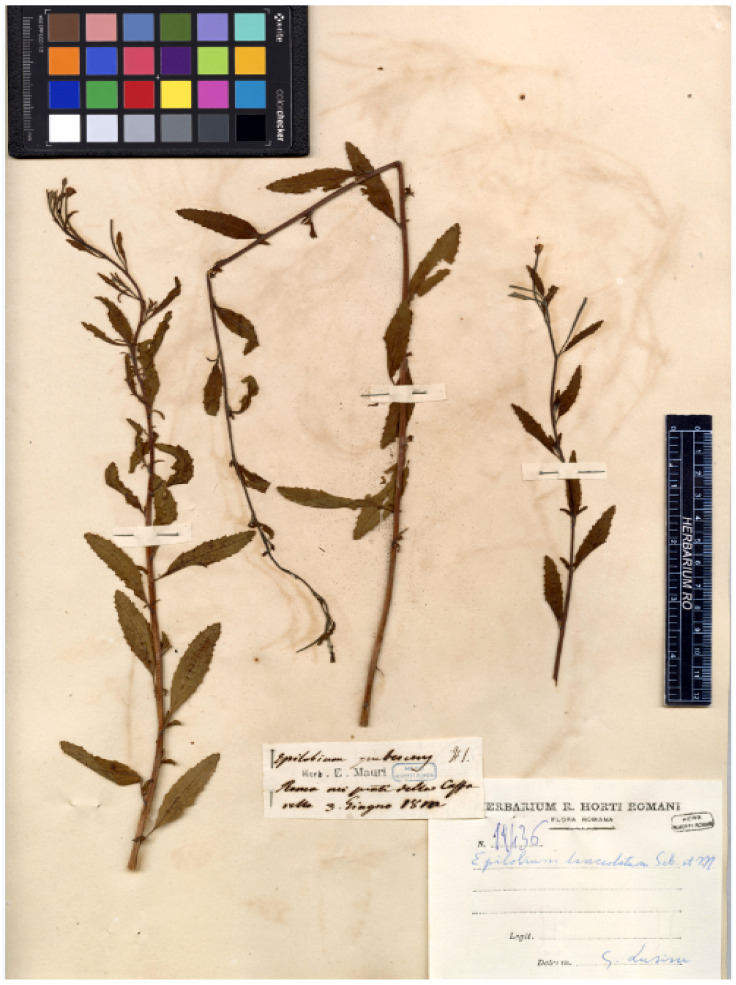
Lectotype of *Epilobium lanceolatum* (RO no. 19436).

***Epilobium lanceolatum* Sebast. & Mauri**: this species was described in 1818 in *Florae Romanae Prodromus* [19] pag. 138. *Locus classicus* includes the locality Caffarella (“In umbrosis, ad oras nemorum, sepes circa Romam frequens. Copiosamente intorno ai boschetti della Caffarella presso la Grotta di Egeria...” = In the shades, at the edge of the woods, hedges about Rome. Copiously around small forest of Caffarella near Egeria’s cave). Iamonico and collaborators [100] designated as lectotype a specimen (deposited at RO; Figure 72) collected by E. Mauri and A. Sebastiani on 3 June 1812.

**Figure 73:**
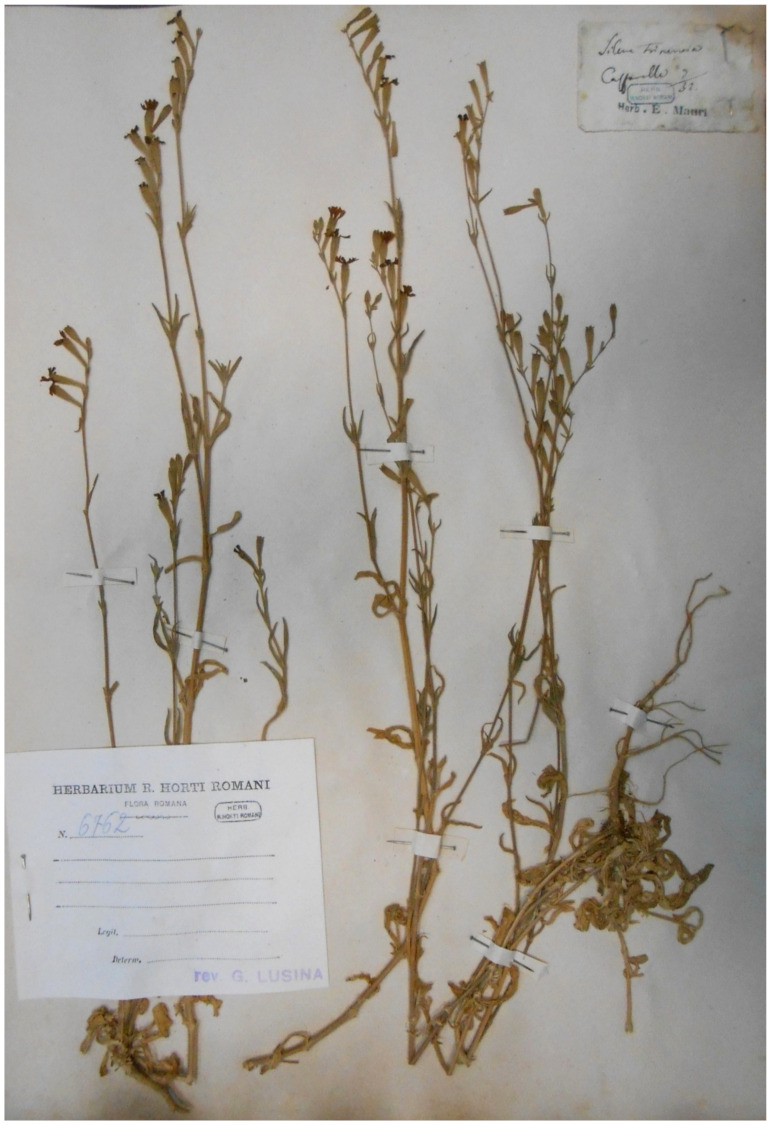
Epitype of *Silene trinervia* (RO-Herbarium Romano no. 6762).

***Silene trinervia* Sebast. & Mauri**: this species was described in 1818 in *Florae Romanae Prodromus* ([19] pag. 152), and *locus classicus* includes the locality Caffarella (“Alla Caffarella presso il Fonte di Egeria...” = At Caffarella near Egeria’s spring). Lacking specimens of original material, Iamonico [101] designated an iconography (Table II in [19]) as lectotype; further, a specimen (deposited at RO; Figure 73) collected by E. Mauri in Caffarella locality in July 1832 was designated as epitype. *Silene trinervia* is currently a heterotypic synonym of *S. gallinyi* Rchb.

**Figure 74:**
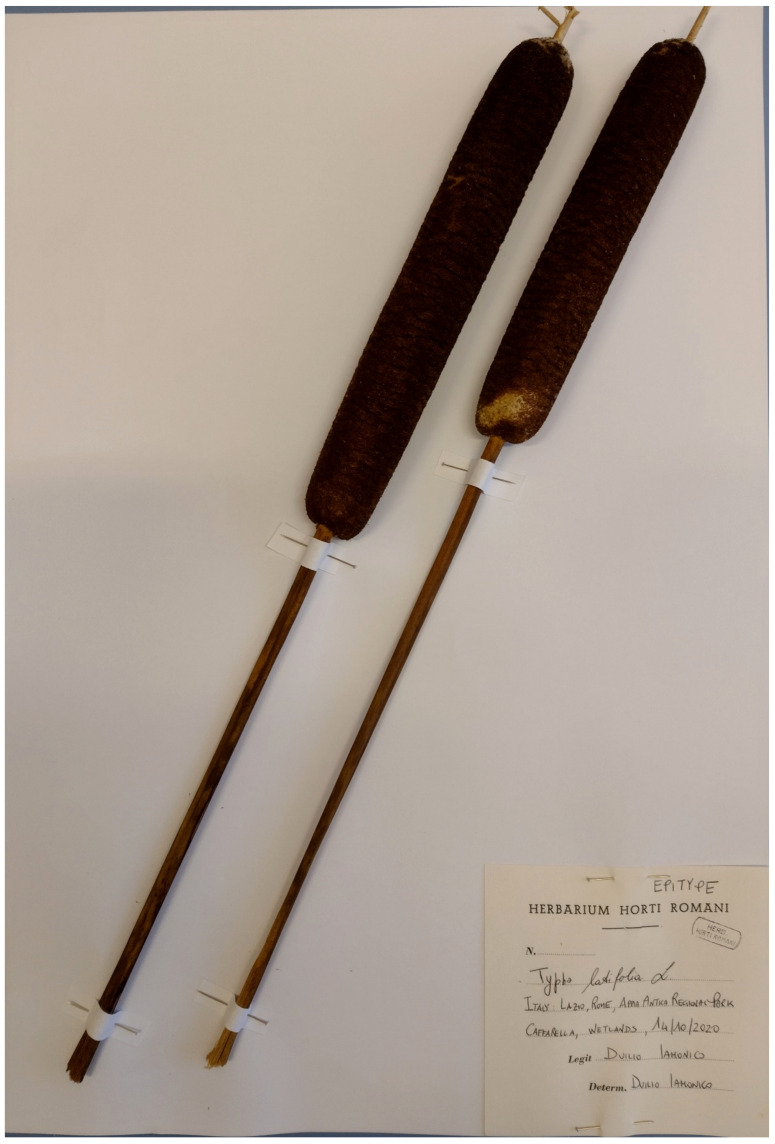
Epitype of *Typha latifolia* (RO s.n.).

***Typha latifolia* L.**: on the basis of a recent study on some Linnaean names of aquatic plants by Iamonico and Iberite [102], an epitype was designated for this Linnaean name. The exsiccatum, deposited at RO (Figure 74), was collected in Caffarella valley by D. Iamonico on 14 October 2020. The population from which the epitype was collected is that shown in Figure 28 of the present paper.

#### 4.4.8. Other Notable Species

**Figure 75:**
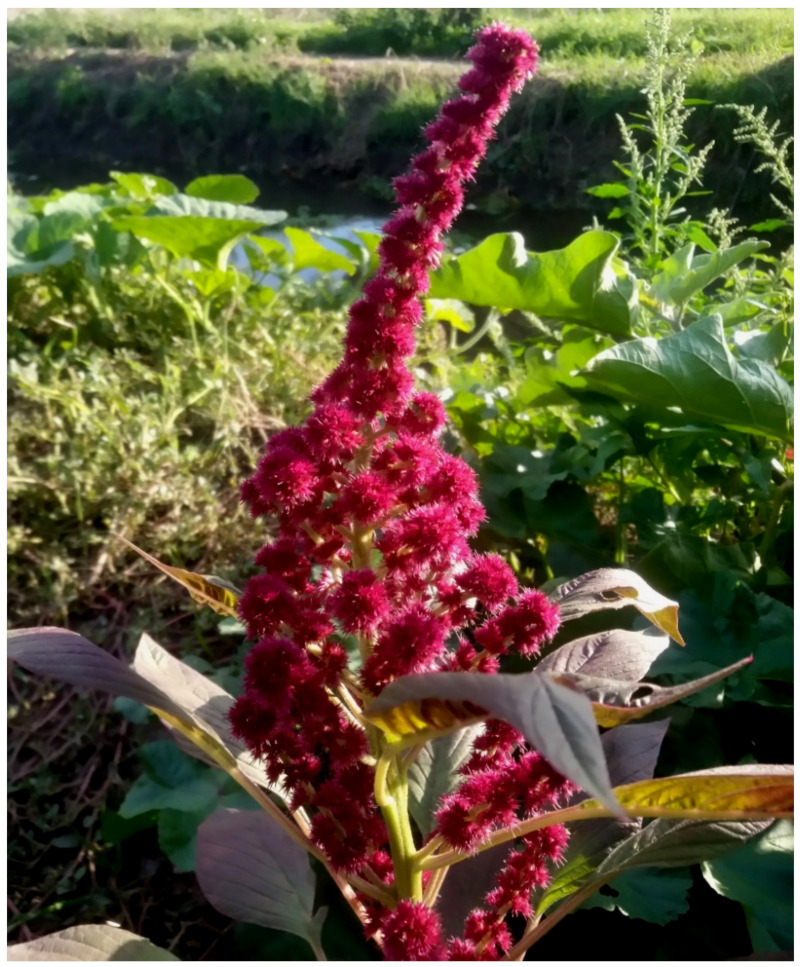
*Amaranthus hypochondricus* in Acquedotti locality.

***Amaranthus hypochondriacus* L.**: this species, indicated as casual alien for Italy [33], was recently reported as naturalized in the Latium region [103] based on two populations occurring in Roma and Frosinone Provinces. The population in Rome refers to 10–15 individuals growing in the territory of Appia Antica Regional Park, i.e., in the Acquedotti locality along the channel Acqua Mariana (Figure 75). The presence of *A. hypochondriacus* has been documented for 6 years.

***Colocasia esculenta* (L.) Schott**: this tropical/subtropical Asian species was recently recorded in Latium for the first time as naturalized (first alien status for Italy). The population was found in the Acquedotti locality along channel *Acqua Mariana* (first observation in 2015, last one in 2019 according to Iamonico [104]). The author, by comparing the climate features of Rome and the native distribution range of *C. esculenta*, showed that the occurrence of this species in Rome is probably linked to micro-climatic factors, i.e., (1) soil (sandy sediments submerged during autumn and winter seasons, well-drained, and partially soaked during spring and summer); (2) brightness (low light intensity, which characterizes the site over almost the entire day); (3) air (high humidity related to both the morphology of the site (a gorge) and the close occurrence of a small waterfall (height: 2.5–3.0 m).

**Figure 76:**
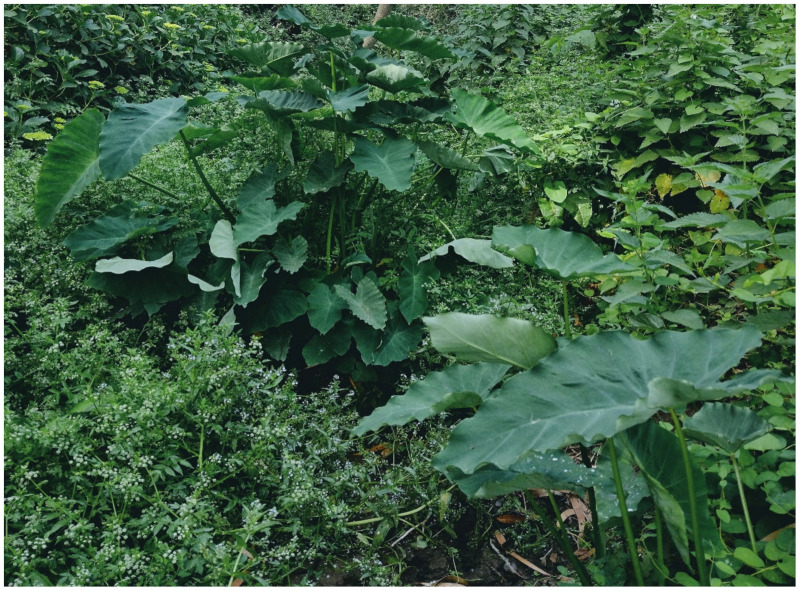
*Colocasia esculenta* along channel Acqua Mariana (Acquedotti locality).

I continue to control the population and found it also during the next years (2021 and 2022; Figure 76).

**Figure 77:**
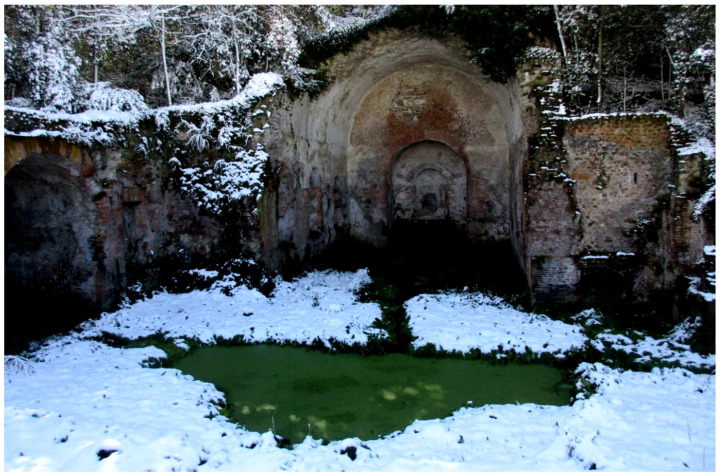
*Lemna minuta* in aquatic habitat of *Egeria nymphaeum* during snowfall in February 2018 (Caffarella valley).

***Lemna minuta* Kunth**: this South American species was first discovered in the Latium region based on collections made in 2007, of which one refers to the Caffarella valley [105]. I am continuing to control the various populations, founding many others both in Caffarella valley and in other parts (mostly in Acquedotti and Tor Marancia localities) where the abundance of the autochthonous *L. minor* decreased over time. Note that the populations of *L. minuta* even survived well through snowfall (Figure 77).

**Figure 78:**
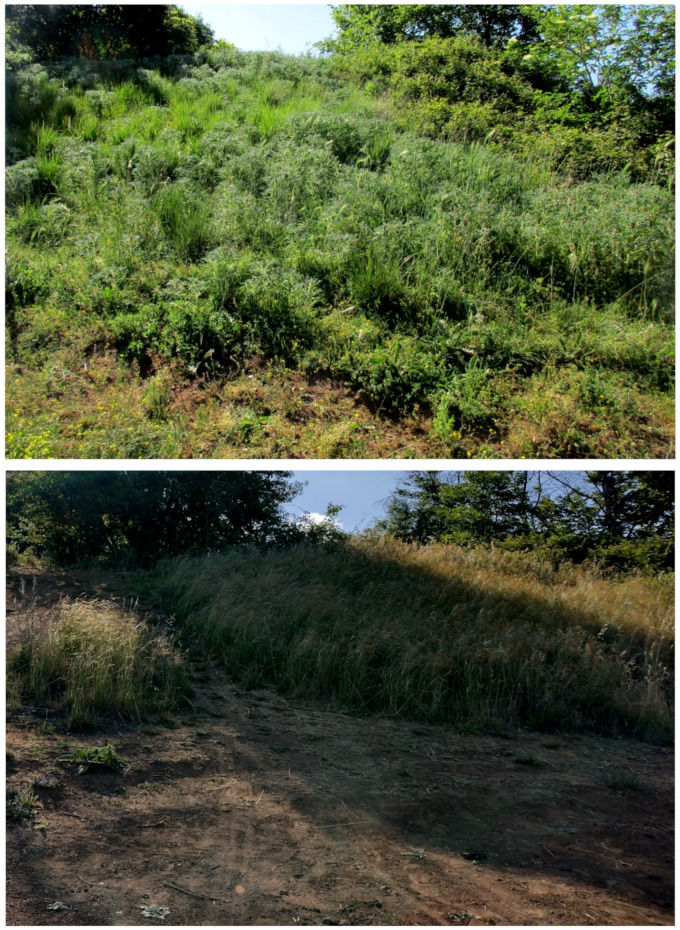
*Lupinus albus* subsp. *graecus* in Caffarella valley: population lost (**top** photo, dated 2017); *Nassella neesiana* dominated community (**bottom** photo, dated 2022).

**Figure 79:**
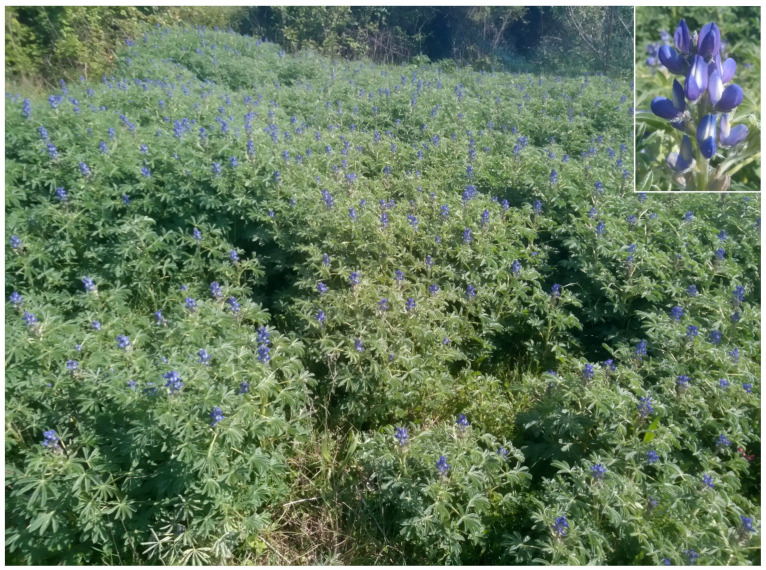
*Lupinus albus* subsp. *graecus* in Caffarella valley: new population found.

***Lupinus albus* L. subsp. *graecus* (Boiss. et Spruner) Franco & Pinto da Silva**: 11 localities of this taxon currently occur in the Latium region, one referring to Caffarella valley [25,106]. I monitored this population over the years, but in 2018 the volcanic slope on which it occurred was destroyed to build paths (Figure 78). During the next 2 years, a part of the area previously occupied by *L. albus* subsp. *graecus* was recolonized by the South American alien *Nassella neesiana* (Trin. et Rupr.) Barkworth (Figure 78); the population of *L. albus* subsp. *graecus* recorded by [25,106] is extinct. Fortunately, I found another population not far from the lost one (never seen before 2018) composed of tens of individuals blooming and fruiting regularly (Figure 79).

**Figure 80:**
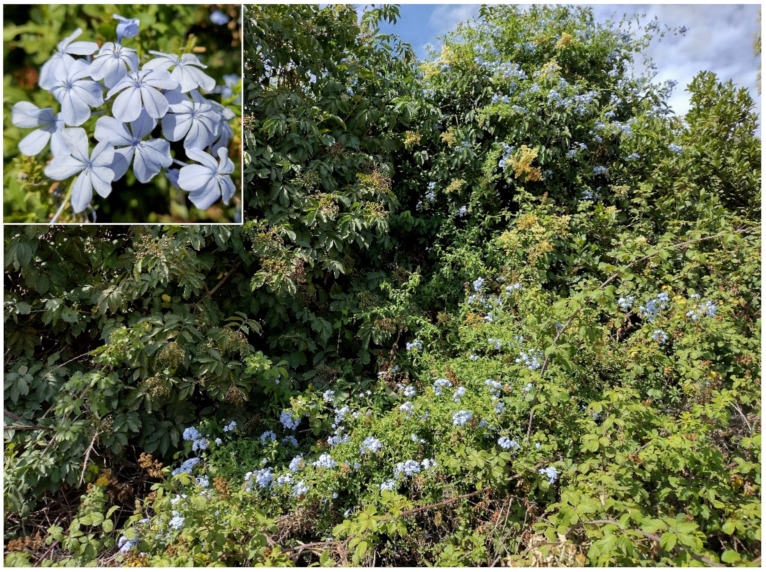
*Plumbago auriculata* in shrubby vegetation (Caffarella locality).

***Plumbago auriculata* Lam.**: this species was recently recorded in Latium (year 2016) as casual [107]. My discovery in the northeastern sector of Caffarella valley represents the second record for Latium. The population found occurs in shrubby vegetation dominated by *Rubus ulmifolium* and *Sambucus nigra* (Figure 80).

## 5. Discussion and Conclusions

The present study provides the first comprehensive inventory of the flora of Appia Antica Regional Park, one of the largest protected urban areas in Europe.

**Figure 81:**
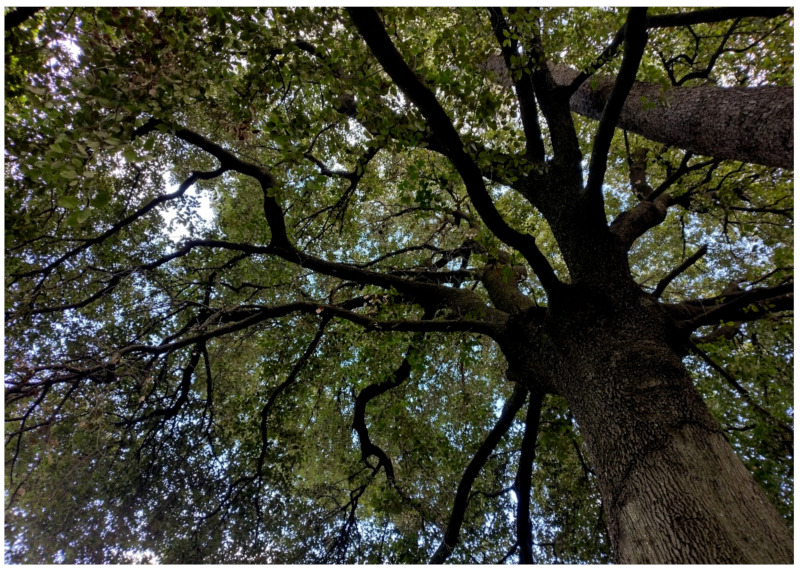
Large individual of *Quercus ilex* subsp. *ilex* in Caffarella valley.

**Figure 82:**
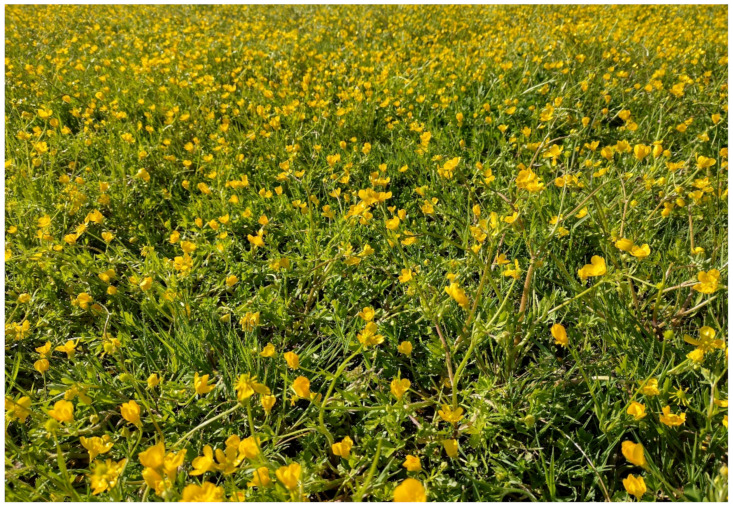
Humid meadow dominated by *Ranunculus repens* L. (Caffarella valley).

The data presented revealed an extraordinary species diversity of the flora, which comprises 714 taxa representing about 43% of the flora of Rome (1649 taxa according to [9]) and about 20% of the flora of the Latium region (3593 taxa according to [108]). This notable datum is mainly linked to the high landscape heterogeneity of the park, which comprises several types of habitats, from those strictly natural (e.g., broad-leaved forests, Mediterranean macchia, Mediterranean riparian woods, humid meadows, helophytic vegetation, etc.) to those strictly anthropogenic (i.e., the segetal and ruderal communities and floras of, respectively, arable crops and disturbed/human-made sites). The main landscape environmental characteristics of the studied area can be, therefore, summarized as:

(1) the persistence of residual patches of natural vegetation (especially in the northern sector of the park), notably concerning those forests (Figure 15) that reveal a rich woody flora (106 taxa of Phanerophytes, corresponding to 14.85% of the total flora). Some patches also have high cultural-historical value, e.g., the so-called “Bosco Sacro” (= Sacread Wood) with various centenarian individuals of *Quercus ilex* subsp. *ilex* (see also [25]); other trees, found throughout the park, are very large and have, therefore, high nature conservation value (Figure 81);

(2) the occurrence of quite well preserved aquatic habitats and humid meadows (mostly in Caffarella valley and Tor Marancia locality; Figure 82) that include taxa and vegetation communities not so common in urban areas where their general decline is a widely acknowledged trend across not only Rome [9], but also worldwide [109,110]. Of note, some of these habitats are included in the Annex I of the Habitat Directive of 21 May 1992 (formally known as Council Directive 92/43/EEC on the Conservation of natural habitats and of wild fauna and flora), e.g., that named “*Salix alba* and *Populus alba* galleries” (code 92A0; see Figure 22).

(3) a rich anthropogenic flora occuring in cultivated lands (segetal taxa; Figure 29) and human-made habitats (ruderal taxa), e.g., those trampled (Figure 31) and/or grasslands and pastures occurring on nutrient-rich soils (e.g., Figure 33 and Figure 34). Notably, this type of flora plays an important role as a source of floristic richness in metropolitan areas [9,111,112,113]. It is also noteworthy that some species of conservation interest grow in anthropogenic environments, as crop wild relatives [114,115].

**Figure 83:**
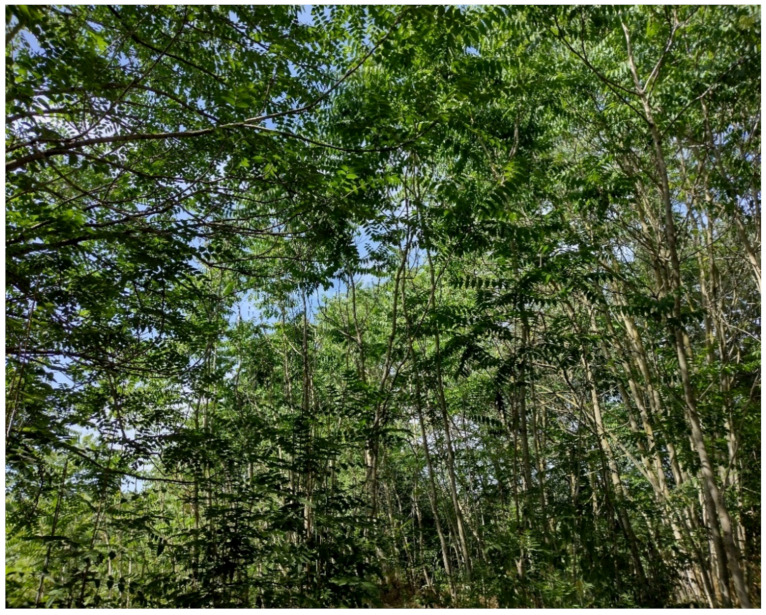
Anthropogenic woody vegetation dominated by *Ailanthus altissima* (Caffarella valley).

**Figure 84:**
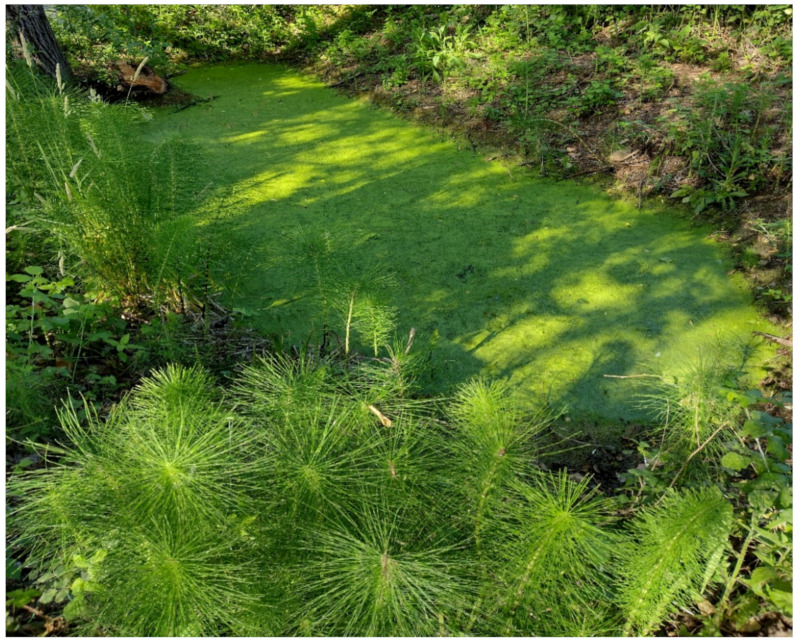
*Lemna minuta* monophytic community on a pond in Caffarella valley.

**Figure 85:**
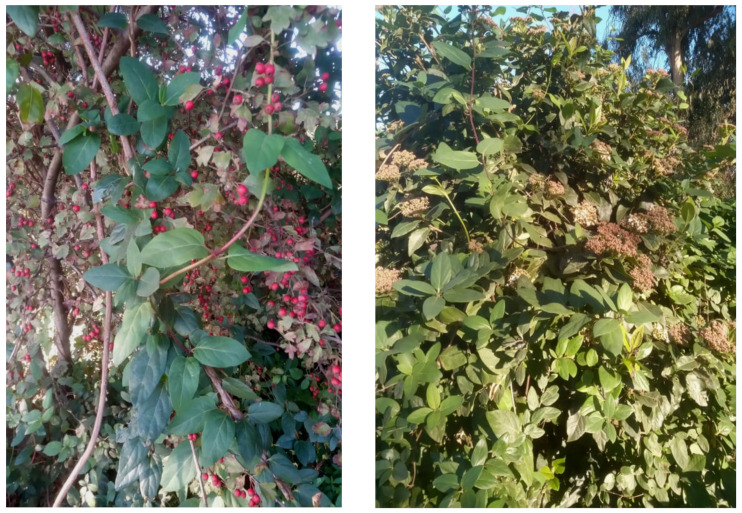
*Lonicera japonica* on *Crataegus monogyna* (**left** photo) and *Viburnum tinus* (**right** photo) (Acquedotti locality).

**Figure 86:**
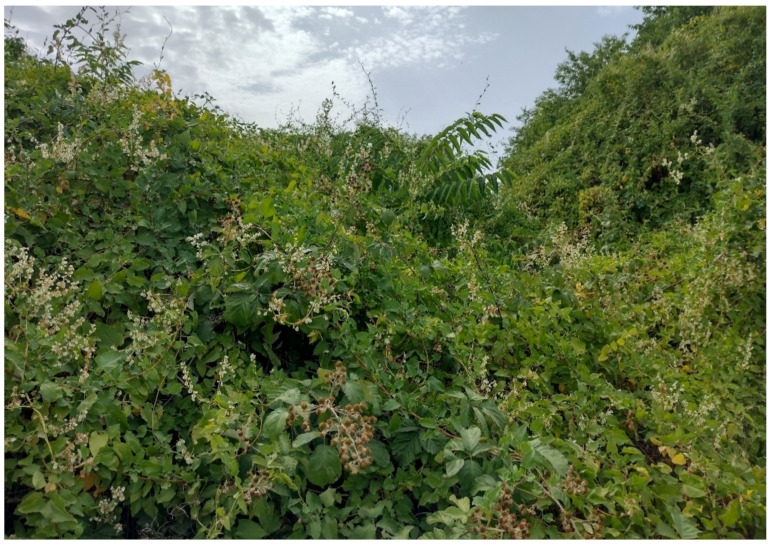
*Fallopia baldschuanica* on shrubby vegetation along a path running near Appia Nuova street.

The anthropogenic flora includes, in addition to native species well-adapted to human disturbance, exotic taxa that decrease the quality of the biodiversity and can negatively impact the natural vegetation. Some naturalized and invasive species are particularly dangerous in the territory of the park and threaten native ones; examples are:

> *Robinia psudoacacia* and *Ailathuts altissima*: these two trees (both invasive in the park) are common along edges of forests and into shrubby mantels, as well as in human-made habitats and on ruins. In various cases, *R. psudoacacia* and *A. altissima* even form more or less dense woody communities (Figure 17 and Figure 83);
> *Lemna minuta*: a North American natant hydrophyte, invasive in the park (Figure 84), which caused the decrease (or even the local extinction) of several populations of the autochthonous *L. minor* (see also [116]);
> *Lonicera japonica* Thumb.: this species is considered to be a naturalized alien in the park, occurring in several sites. In some cases (e.g., along banks of channel *Acqua Mariana*, in the Acquedotti locality), it forms dense populations that cover shrubby native vegetation communities of *Rubus ulmifolius* Schott, *Cornus sanguinea* L., *Crataegus monogyna* Jacq., *Phillyrea latifolia* L. and *Viburnum tinus* L. (Figure 85), or climbs on young trees of *Populus nigra* L.;
> *Fallopia baldschuanica* (Regel) Holub: this climbing species is naturalized in the park, where it forms often dense populations growing on shrubs (Figure 86) and competing with autochthonous liana taxa, e.g., *Clematis vitalba* L., *Humulus lupulus* L., or *Lonicera etrusca* Santi.

**Figure 87:**
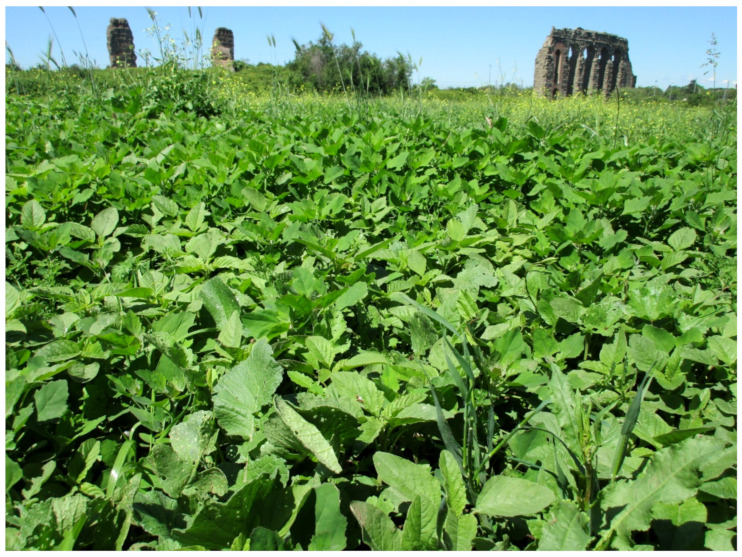
Dense population of young individuals of *Amaranthus sp. pl.* on a crop (Acquedotti locality).

Concerning the segetal flora, some allochthonous species impact the wheat crops from an economic point of view, since populations densely grow on fields, imposing significantly increased costs for their management and reducing wheat yields [29,117]. *Amaranthus retroflexus* L. and *A. hybridus* L. are the main dangerous species observed in the park (Figure 87).

(4) the flora of archeological sites: these areas have a significant role in enhancing plant diversity in cities, being refuges of natural flora and vegetation in the urban ecosystems [9,118,119,120].

**Figure 88:**
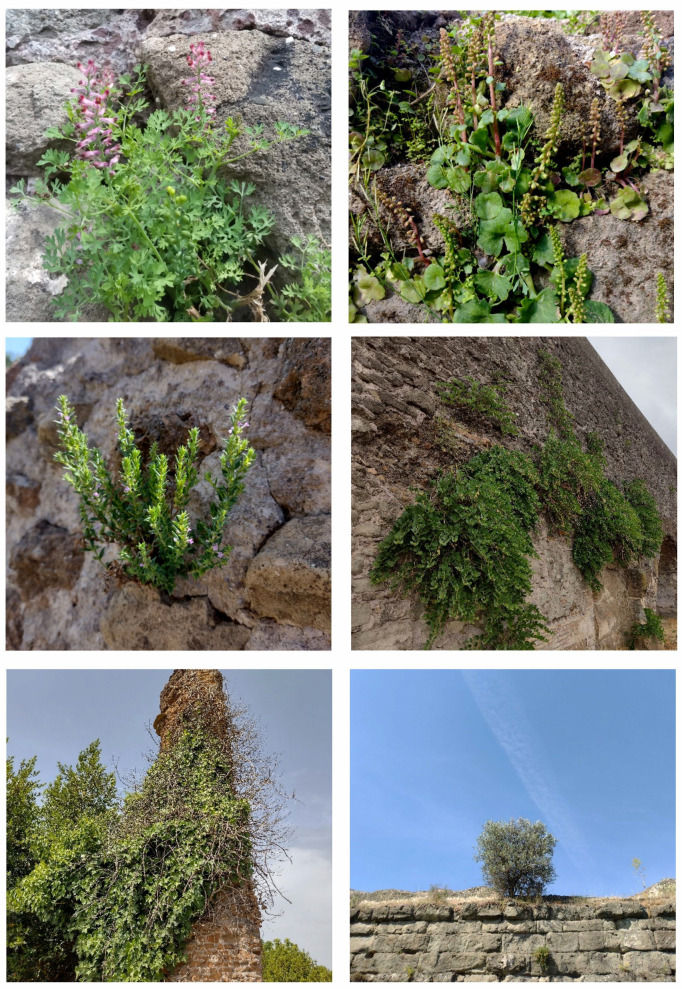
*Fumaria officinalis* subsp. *officinalis* on Felix’s Aqueduct (top-left photo); *Umbilicus rupestris* on the vertical wall of the aqueduct located in front of the *Egeria* nymphaeum, in Caffarella valley (top-right photo); *Micromeria graeca* on Claudio’s Aqueduct (central-left photo); *Capparis orientalis* on Felix’s Aqueduct (central-right photo); *Hedera helix* subsp. *helix* on Felix’s Aqueduct (bottom-left photo); *Olea europaea* on Claudio’s Aqueduct (bottom-right photo).

Appia Antica Regional Park is rich in archeological elements (e.g., aqueducts, catacombs, churches, tombs, sepulchers, temples, etc.) that also significatively contribute to the landscape structure (see Section “3.5. Landscape remarks and actual vegetation physiognomies”). Several taxa occur on both the top and vertical walls of the ruins (see also [120]), and they consist of herbs (therophytes, hemicryptophytes, and geophytes), subshrubs (chamaephytes), shrubs (caespitose phanerophytes), or even trees (scapose phanerophytes). Various species have cliffs or rocky places as their natural habitat and found the same dry environmental conditions by growing on archeological remains, which, therefore, can be considered as secondary habitats for them; examples of species with different biological forms that occur in the park are (Figure 88): *Fumaria officinalis* L. subsp. *officinalis* (therophyte), *Umbilicus rupestris* (Salisb.) Dandy (bulbose geophyte), *Micromeria graeca* (L.) Benth. ex Rchb. (suffruticose chamaephyte), *Capparis orientalis* (nano-phanerophyte), *Hedera helix* L. subsp. *helix* (liana), and *Olea europea* L (scapose phanerophyte).

In some cases, the archeological sites allow the presence of species not common in the whole region of Latium. An example is *Parietaria lusitanica* L. subsp. *lusitanica*, which occurs in Rome territory mostly in the northern sector, whereas only three scattered populations are currently recorded toward the south, and one of them is located in Appia Antica Regional Park on walls along Appia Antica street near Cecilia Metella sepulcher [121].

**Figure 89:**
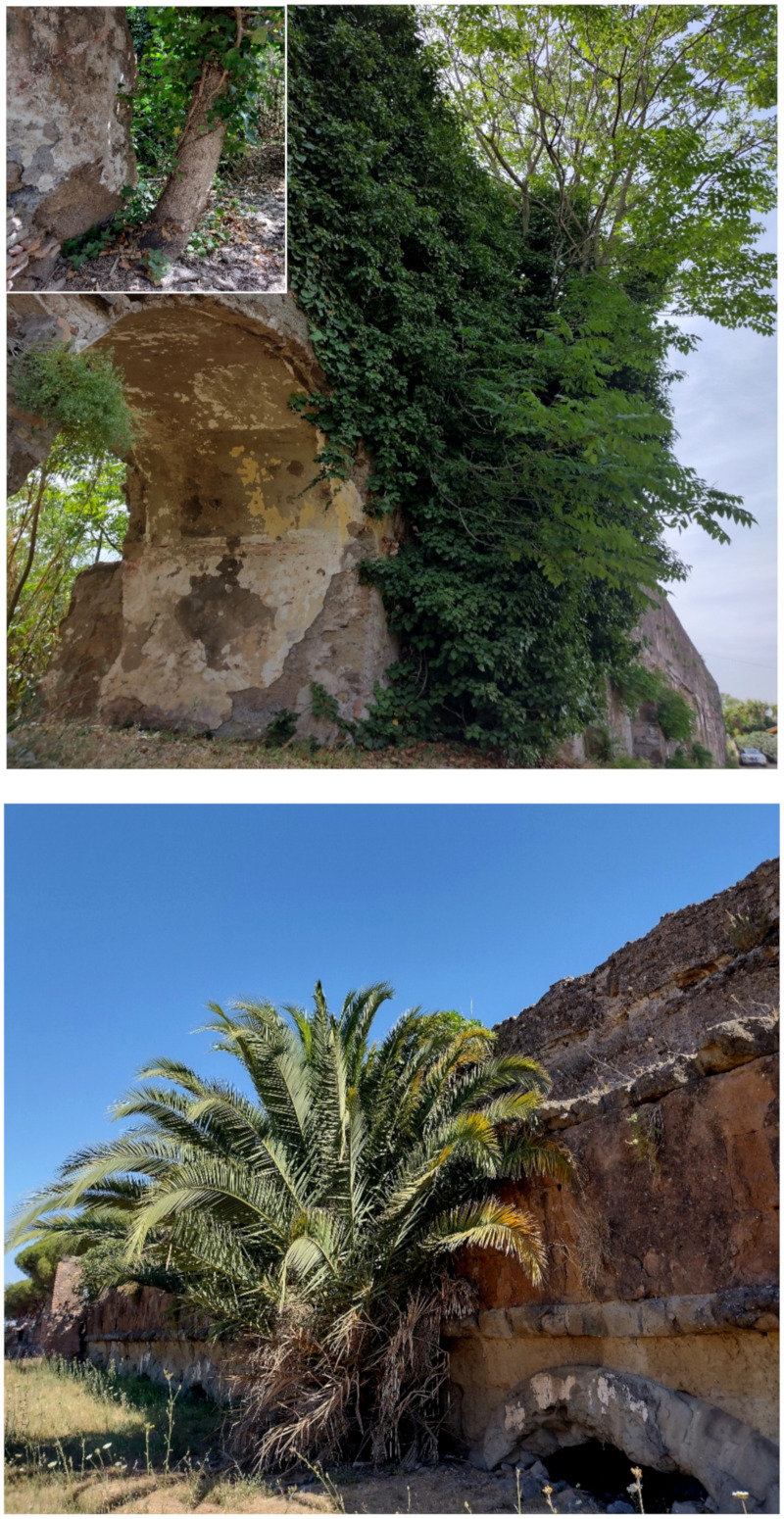
*Ailanthus altissima* (**top** photo) and *Phoenix canariensis* (**bottom** photo) at the base of Felix’s Aqueduct (Tor Fiscale and Acquedotti localities, respectively).

Finally, alien species also occur on these sites and they can inflict damage on their structures, mainly due to their roots, which induce both chemical and mechanical forms of deterioration [122]. Examples for Appia Antica Park are *Ailanthus altissima*, *Phoenix canariensis* H.Wildpret, and *Robinia pseudoacacia*, which can be observed at the base or on the vertical walls of ancient Roman aqueducts (Figure 89).

In conclusion this study, by providing (1) an extensive set of floristic data on the species diversity of Appia Antica Regional Park, including its structure in biological, ecological and biogeographical terms and floristic novelties at the regional, national and European levels, (2) an overview of the landscape structure and vegetation physiognomies, and (3) an emphasis on the non-native flora and its ecological, social and economic impacts on autochthonous flora, archeological ruins and crops, gives a general base framework for guiding future scientific and applied researches and landscape action plans. Concerning basic scientific studies, the first one should investigate the vegetation communities in detail (based on the physiognomies listed in the present paper and the Land Use map published by Iamonico [40]) carried out on phytosociological plots. With regard to applied research and landscape action plans, there are many possibilities, e.g., eradication of dangerous alien species (see e.g., [123]), planning of education paths for natural flora and vegetation (see e.g., [124]), urban forestry (see e.g., [125]), etc. All of these research efforts and actions have, as their final aim, the conservation of biodiversity in terms of environmental sustainability [126], providing, in turn and by green infrastructure and ecosystem services implementation, benefits for urban residents in the form of improved human health and well-being (see e.g., [127,128]).

## Figures and Tables

**Table 1:** Strictly (marked with an asterisk) and characteristic segetal species occurring in the territory of Appia Antica Regional Park (names in alphabetical order).

* *Alopecurus myosuroides* Huds, subsp. *myosuroides*
*Anisantha diandra* (Roth) Tutin ex Tzvelev
*Anthemis arvensis* L. subsp. *arvensis*
*Anthemis cotula* L.
* *Aphanes arvensis* L.
*Avena sterilis* L. subsp. *sterilis*
* *Ballota nigra* L. subsp. *meridionalis* (Bég.) Bég.
*Buglossoides arvensis* (L.) I.M.Johnst.
* *Cyanus segetum* L.
* *Delphinium consolida* L. subsp. *consolida*
* *Ervilia hirsuta* (L.) Opiz
* *Ervum tetraspermum* L.
* *Euphorbia exigua* L. subsp. *exigua*
* *Euphorbia falcata* L. subsp. *falcata*
*Filago germanica* (L.) Huds.
*Gladiolus italicus* Mill.
*Herniaria glabra* L.
*Herniaria hirsuta* L. subsp. *hirsuta*
*Lamium purpureum* L.
*Lathyrus annuus* L.
*Lathyrus aphaca* L. subsp. *aphaca*
* *Legousia speculum-veneris* (L.) Chaix subsp. *speculum-veneris*
*Lysimachia arvensis* (L.) U.Manns & Anderb. subsp. *arvensis*
*Matricaria chamomilla* L.
*Muscari comosum* (L.) Mill.
*Myosotis arvensis* (L.) Hill subsp. *arvensis*
*Neslia paniculata* (L.) Desv. subsp. *thracica* (Velen.) Bornm.
*Orobanche crenata* Forssk.
* *Papaver dubium* L.
* *Papaver hybridum* L.
* *Papaver rhoeas* L.
*Phalaris paradoxa* L.
*Rapistrum rugosum* (L.) All.
*Sinapis alba* L. subsp. *alba*
*Sinapis arvensis* L. subsp. *arvensis*
* *Spergula arvensis* L.
*Valerianella eriocarpa* Desv.
*Veronica arvensis* L.
*Veronica polita* Fr.
*Vicia bithynica* (L.) L.

**Table 2:** Phanerophytes (P) occurring in the territory of Appia Antica Regional Park.

	Native Taxa		Alien Taxa	
Biological Form	N° of Taxa	Percentage	N° of Taxa	Percentage
P scapose	30	4.20	11	1.54
P caespitose	22	3.08	10	1.40
P lianose	7	0.98	6	0.84
P succulent	0	0	1	0.14
Nano-phanerophytes	11	1.54	6	0.84
Total	70	9.80	36	5.04

**Table 3:** Floristic novelties discovered during field surveys. Names are ordered alphabetically per type of novelty.

Type of Novelty	Scientific Name
First record for **Europe** and first one out of the native range	*Denisophytum bessac* (Choiv.) E.Gagnon & G.P.Lewis
First confirmed records for **Italy**	*Euphorbia pulcherima* Willd. ex Klotzsch *Rosa chinensis* Jacq. var. *semperflorens* (Curtis) Koehne
First naturalized occurrence for **Italy**	*Aloe maculata* All. subsp. *maculata*
First records for **Latium region**	*Heliotropium amplexicaule* Vahl. *Hydrangea macrophylla* (Thunb.) Ser. *Ruellia simplex* C.Wright *Trachelospermum jasminoides* (Lindl.) Lem.
Change in alien status for **Latium region**, from casual to naturalized	*Campsis radicans* (L.) Bureau *Canna indica* L. *Cyperus alternifolius* L. subsp. *flabelliforme* Kük. *Kalanchoe daigremontiana* Raym.
Confirmation as alien in **Latium region**	*Melia azedarach* L. *Punica granatum* L.
New records for the flora of **Rome**	*Bidens subalternans* DC. *Chlorophytum comosum* (Thumbs.) Jacques *Zantedeschia aetiopica* (L.) Spreng.
Confirmations for the flora of **Rome**	*Anreedera cordifolia* (Ten.) Steenis *Diospyrus kaki* L. *Papaver somniferum* L. *Passiflora caerulea* L.

## Data Availability

Not applicable.

## Appendix A

Inventory of the taxa occurring in Appia Antica Regional Park. Names of families in nonbold and uppercase; groups higher than families in bold and uppercase. Abbreviations: A: alien (with CAS: casual taxon; NAT: naturalized taxon; INV: invasive taxon); E: endemic; C: cryptogenic; NC: no longer recorded (not confirmed, but documented by the literature and/or herbaria specimens) S: segetal taxon (with, if occurring, sS: stritcly segetal taxon; cS: characteristic segetal taxon).

**FERNS And ALLIES**

EQUISETACEAE Michx. ex DC.

***Equisetum arvense*** L., S

***Equisetum ramosissimum*** Desf., S

***Equisetum telmateia*** Ehrh., S

DENNSTAEDTIACEAE Lotsy

***Pteridium aquilinum*** (L.) Kuhn subsp. ***aquilinum***

NEPHROLEPIDACEAE Pic. Serm.

***Nerpholepis cordifolia*** (L.) C.Persl, A NAT

ASPLENIACEAE Newman

***Asplenium onopteris*** L.

***Asplenium scolopendrium*** L. subsp. ***scolopendrium***

***Asplenium trichomanes*** L. subsp. ***quadrivalens*** D.E. Meyer

**GYMNOSPERMS**

PINACEAE Spreng. ex F.Rudolphi

***Pinus halepensis*** Mill., A CAS

***Pinus pinea*** L., A CAS

CUPRESSACEAE Gray

***Cupressus sempervirens*** L., A CAS

**ANGIOSPERMS**

LAURACEAE Juss.

***Laurus nobilis*** L.

ARACEAE Juss.

***Arum italicum*** Mill. subsp. ***italicum***, S

***Biarum tenuifolium*** (L.) Schott subsp. ***tenuifolium***

***Colocasia esculenta*** (L.) Schott, A NAT

***Lemna gibba*** L.

***Lemna minor*** L.

***Lemna minuta*** Kunth, A INV

***Zantedeschia aethiopica*** (L.) Spreng., A NAT

ALISMATACEAE Vent.

***Alisma lanceolatum*** With.

***Alisma plantago-aquatica*** L.

DIOSCOREACEAE R.Br.

***Dioscorea communis*** (L.) Caddick & Wilkin

SMILACACEAE Vent.

***Smilax aspera*** L.

ORCHIDACEAE Juss.

***Anacamptis coriophora*** (L.) R.M.Bateman, Pridgeon & M.W.Chase

***Anacamptis morio*** (L.) R.M.Bateman, Pridgeon & M.W.Chase

***Anacamptis papilionacea*** (L.) R.M.Bateman, Pridgeon & M.W.Chase

***Ophrys apifera*** Huds.

***Ophrys incubacea*** Bianca

***Ophrys sphegodes*** Mill. subsp. ***sphegodes***

***Ophrys tenthredinifera*** Willd.

***Orchis laxiflora*** Lam.

***Serapias lingua*** L.

***Serapias parviflora*** Parl.

***Serapias vomeracea*** (Burm. fil.) Briq.

***Spiranthes spiralis*** (L.) Chevall.

IRIDACEAE Juss.

***Crocus biflorus*** Mill.

***Gladiolus italicus*** Mill., cS

***Iris germanica*** L., A NAT

***Limniris pseudacorus*** (L.) Fuss

***Romulea bulbocodium*** (L.) Sebast. et Mauri

***Romulea columnae*** Sebast. et Mauri

ASPHODELACEAE Dumort.

***Aloe maculata*** All., A NAT

***Asphodelus ramosus*** L. subsp. ***ramosus***

AMARYLLIDACEAE J.St.-Hil.

***Allium ampeloprasum*** L.

***Allium chamaemoly*** L. subsp. ***chamaemoly***

***Allium neapolitanum*** Cirillo

***Allium roseum*** L., S

***Allium triquetrum*** L.

***Allium vineale*** L., S

***Ipheion uniflorum*** (Lindl.) Raf., A CAS

***Leucojum aestivum*** L., A CAS

***Narcissus pseudonarcissus*** L., A CAS

***Narcissus tazetta*** L. subsp. ***tazetta***, S

***Sternbergia lutea*** (L.) Ker-Gawler

ASPARAGACEAE Juss.

***Agave americana*** L., A CAS

***Asparagus acutifolius*** L.

***Asparagus offininalis*** L.subsp. ***officinalis***

***Bellevalia romana*** (L.) Reichenb., S

***Chlorophytum comosum*** (Thunb.) Jacques, A CAS

***Muscari comosum*** (L.) Mill., cS

***Muscari neglectum*** Guss. ex Ten.

***Loncomelos narbonense*** (L.) Raf., S

***Ornithogalum divergens*** Boreau, S

***Ruscus aculeatus*** L.

***Yucca gloriosa*** L., A CAS

ARECACEAE Bercht. & J.Presl

***Chamaerops humilis*** L., A CAS

***Phoenix canariensis*** H. Wildpret, A CAS

TYPHACEAE Juss.

***Sparganium erectum*** L.

***Typha latifolia*** L.

CANNACEAE Juss.

***Canna indica*** L., A NAT

JUNCACEAE Juss.

***Juncus articulatus*** L.

***Juncus inflexus*** L. subsp. ***inflexus***

***Luzula campestris*** (L.) DC. subsp. ***campestris***

***Luzula forsteri*** (Sm.) DC.

CYPERACEAE Juss.

***Carex distachya*** Desf.

***Carex distans*** L.

***Carex divisa*** Hudson

***Carex flacca*** Schreb. subsp. ***erythrostachys*** (Hoppe) Holub

***Carex hirta*** L.

***Carex otrubae*** Podp.

***Carex pendula*** Hudson

***Cyperus alternifolius*** L. subsp. ***flabelliformis*** Kük., A NAT

***Cyperus badius*** Desf.

***Cyperus longus*** L.

***Cyperus rotundus*** L., S

***Eleocharis palustris*** (L.) Roem. et Schult., S

***Scirpoides holoschoenus*** (L.) Soják

POACEAE Barnhart

***Agrostis stolonifera*** L. subsp. ***stolonifera***, S

***Aira cupaniana*** Guss., S

***Aira elegans*** Willd. subsp. ***elegans***

***Alopecurus myosuroides*** Hudson subsp. ***myosuroides***, sS

***Anisantha diandra*** (Roth) Tutin ex Tzvelev, cS

***Anisantha madritensis*** (L.) Nevski subsp. ***madritensis***, S

***Anisantha rigida*** (Roth) Hyl., S

***Anisantha sterilis*** (L.) Nevski, S

***Arundo donax*** L., A INV

***Arundo plinii*** Turra, S

***Avena barbata*** Pott. ex Link subsp. ***barbata***

***Avena sativa*** L. subsp. ***sativa***, A NAT

***Avena sterilis*** L. subsp. ***sterilis***, cS

***Brachypodium distachyon*** (L.) P. Beauv., S

***Brachypodium rupestre*** (Host) Roemer et Schultes, S

***Brachypodium sylvaticum*** (Huds.) Beauv. subsp. ***sylvaticum***

***Bromopsis erecta*** (Huds.) Fourr., S

***Bromus commutatus*** Schrad., S

***Bromus hordeaceus*** L. subsp. ***hordeaceus***, S

***Catabrosa aquatica*** (L.) P. Beauv., NC

***Catapodium rigidum*** (L.) C.E. Hubb., S

***Cynodon dactylon*** (L.) Pers., S

***Dactylis glomerata*** L. subsp. ***glomerata***, S

***Dactylis glomerata*** L. subsp. ***hispanica*** (Roth) Nyman

***Dasypyrum villosum*** (L.) P. Candargy, S

***Digitaria sanguinalis*** (L.) Scop., S

***Echinochloa crus-galli*** (L.) Beauv., S

***Ehrharta erecta*** Lam., A CAS

***Eleusine indica*** (L.) Gaertn.

***Elymus repens*** (L.) Gould subsp. ***repens***, S

***Festuca danthonii*** Asch. & Graebn. subsp. ***danthonii***, S

***Festuca ligustica*** (All.) Bertol., S

***Festuca myuros*** L. subsp. **myuros**, S

***Gaudinia fragilis*** (L.) Beauv., S

***Holcus lanatus*** L., S

***Hordeum bulbosum*** L.

***Hordeum murinum*** L. subsp. ***leporinum*** (Link) Arcang., S

***Hordeum murinum*** L. subsp. ***murinum***, S

***Hordeum secalinum*** Schreb.

***Hyparrhenia hirta*** (L.) Stapf subsp. ***hirta***

***Imperata cylindrica*** (L.) Raeusch.

***Lagurus ovatus*** L.

***Lolium arundinaceum*** (Schreb.) Darbysh. subsp. ***arundinaceum***

***Lolium multiflorum*** Lam. subsp. ***multiflorum***, S

***Lolium perenne*** L., S

***Lolium rigidum*** Gaudin subsp. ***rigidum***, S

***Macrobriza maxima*** (L.) Tzvelev, S

***Nassella neesiana*** (Trin. et Rupr.) Barkworth, A NAT

***Oloptum miliaceum*** (L.) Röser & H.R.Hamasha

***Parapholis cylindrica*** (Willd.) Romero Zarco, NC

***Paspalum distichum*** L.

***Phalaris aquatica*** L., S

***Phalaris paradoxa*** L., cS

***Phleum pratense*** L. subsp. ***pratense***, S

***Phragmites australis*** (Cav.)Trin. ex Steud. subsp. ***australis***, S

***Phyllostachys reticulata*** (Rupr.) K.Koch, A NAT

***Poa annua*** L., S

***Poa bulbosa*** L., S

***Poa pratensis*** L., S

***Poa trivialis*** L., S

***Polypogon monspeliensis*** (L.) Desf., S

***Rostraria cristata*** (L.) Tzvelev

***Setaria italica*** (L.) P.Beauv. subsp. ***viridis*** (L.) Thell., A NAT, S

***Setaria verticillata*** (L.) P.Beauv.

***Sorghum halepense*** (L.) Pers., S

***Trisetaria panicea*** (Lam.) Paunero, S

***Triticum aestivum*** L., A CAS

***Triticum negletum*** (Req. ex Bertol.) Greuter, S

***Triticum vagans*** (Jord. & Fourr.) Greuter, S

RANUNCULACEAE Juss.

***Anemone hortensis*** L., S

***Clematis vitalba*** L.

***Delphinium consolida*** L. subsp. ***consolida***, sS

***Delphinium halteratum*** Sm. subsp. ***halteratum***

***Ficaria verna*** Huds. subsp. ***ficariiformis*** (F.W.Schultz) B.Walln., S

***Nigella damascena*** L., S

***Ranunculus bulbosus*** L., S

***Ranunculus lanuginosus*** L., S

***Ranunculus parviflorus*** L., S

***Ranunculus peltatus*** Schrank

***Ranunculus repens*** L., S

***Ranunculus sardous*** Crantz, S

***Thalictrum aquilegiifolium*** L.

PLATANACEAE T.Lestib.

***Platanus hispanica*** Mill. ex Münchh.

PAPAVERACEAE Juss.

***Chelidonium majus*** L.

***Fumaria capreolata*** L. subsp. ***capreolata***, S

***Fumaria officinalis*** L. subsp. ***officinalis***, S

***Papaver dubium*** L., C, sS

***Papaver hybridum*** L., sS

***Papaver rhoeas*** L., C, sS

***Papaver somniferum*** L.

CRASSULACEAE J.St.-Hil.

***Kalanchoe daigremontiana*** Raym., A NAT

***Petrosedum sediforme*** (Jacq.) Grulich subsp. ***sediforme***

***Phedimus stellatus*** (L.) Raf.

***Sedum caespitosum*** (Cav.) DC.

***Sedum cepaea*** L.

***Umbilicus horizontalis*** (Guss.) DC.

***Umbilicus rupestris*** (Salisb.) Dandy

SAXIFRAGACEAE Juss.

***Saxifraga trydactilites*** L., S

VITACEAE Juss.

***Parthenocissus quinquefolia*** (L.) Planch., A NAT

***Vitis vinifera*** L., A NAT

ZYGOPHYLLACEAE R.Br.

***Tribulus terrestris*** L.

FABACEAE Lindl.

***Acacia dealbata*** Link, A NAT

***Astragalus glycyphyllos*** L., S, NC

***Astragalus hamosus*** L., S

***Astragalus pelecinus*** (L.) Barneby

***Bituminaria bituminosa*** (L.) C.H.Stirt.

***Cercis siliquastrum*** L. subsp. ***siliquastrum***

***Cytisus villosus*** Pourr.

***Denisophytum******bessac*** (Choiv.) E.Gagnon & G.P.Lewis, A NAT

***Emerus major*** Mill. subsp. ***major***

***Ervilia hirsuta*** (L.) Opiz, sS

***Ervum tetraspermum*** L., sS

***Galega officinalis*** L., S

***Gleditsia triacanthos*** L., A CAS

***Hymenocarpos circinnatos*** (L.) Savi

***Lathyrus annuus*** L., cS

***Lathyrus aphaca*** L. subsp. ***aphaca***, cS

***Lathyrus cicera*** L., S

***Lathyrus clymenum*** L., S

***Lathyrus latifolius*** L., S

***Lathyrus oleraceus*** Lam. subsp. ***biflorus*** (Raf.) H.Schaef., Coulot & Rabaute

***Lotus corniculatus*** L., S

***Lotus ornithopodioides*** L.

***Lotus tenuis*** Waldst. & Kit. ex Willd., S

***Lupinus angustifolius*** L.

***Lupinus albus*** L. subsp. ***graecus*** (Boiss. & Spruner) Franco & P.Silva

***Medicago arabica*** (L.) Huds., S

***Medicago falcata*** L. subsp. ***falcata***, S

***Medicago lupulina*** L., S

***Medicago minima*** (L.) L., S

***Medicago orbicularis*** (L.) Bartal., S

***Medicago polymorpha*** L.

***Medicago rigidula*** (L.) All., S

***Medicago sativa*** L.

***Onobrychis viciifolia*** Scop.

***Ononis spinosa*** L. subsp. ***antiquorum*** (L.) Arcangeli

***Ornithopus compressus*** L., S

***Robinia pseudoacacia*** L., A INV

***Scorpiurus muricatus*** L., S

***Securigera cretica*** (L.) Lassen, S

***Securigera securidaca*** (L.) Degen et Dörfler, S

***Spartium junceum*** L.

***Trifolium angustifolium*** L. subsp. ***angustifolium***, S

***Trifolium arvense*** L., S

***Trifolium campestre*** Schreb., S

***Trifolium cherleri*** L., S

***Trifolium echinatum*** M. Bieb., S

***Trifolium incarnatum*** L. subsp. ***incarnatum***

***Trifolium incarnatum*** L. subsp. ***molinerii*** (Balb. ex Hornem.) Ces.

***Trifolium ligusticum*** Balb. ex Loisel.

***Trifolium micranthum*** Viv.

***Trifolium nigrescens*** Viv. subsp. ***nigrescens***, S

***Trifolium pratense*** L. subsp. ***pratense***, S

***Trifolium repens*** L., S

***Trifolium resupinatum*** L., S

***Trifolium scabrum*** L., S

***Trifolium squarrosum*** L.

***Trifolium stellatum*** L., S

***Trifolium subterraneum*** L., S

***Trifolium suffocatum*** L.

***Trifolium tomentosum*** L., S

***Trifolium vesiculosum*** Savi

***Trigonella alba*** (Medik.) Coulot & Rabaute

***Trigonella altissima*** (Thuill.) Coulot & Rabaute

***Trigonella smalii*** Coulot & Rabaute

***Vicia angustifolia*** L., S

***Vicia bithynica*** (L.) L., cS

***Vicia dasycarpa*** Ten., S

***Vicia faba*** L., A CAS

***Vicia hybrida*** L., S

***Vicia incana*** Gouan

***Vicia melanops*** Sm.

***Vicia narbonensis*** L., S

***Vicia sativa*** L.

***Vicia serratifolia*** Jacq.

ROSACEAE Juss.

***Aphanes arvensis*** L., sS

***Agrimonia eupatoria*** L. subsp. ***eupatoria***, S

***Crataegus monogyna*** Jacq.

***Geum urbanum*** L.

***Malus domestica*** (Suckow) Borkh., A CAS

***Malus sylvestris*** Mill.

***Potentilla recta*** L., S

***Potentilla reptans*** L., S

***Poterium sanguisorba*** L. subsp. ***balearicum*** (Bourg. ex Nyman) Stace, S

***Prunus cerasifera*** Ehrh. var. pissardii (Carrière) C.K.Schneid

***Prunus domestica*** L., A CAS

***Prunus spinosa*** L.

***Pyracantha coccinea*** M.Y.Roemer

***Pyrus communis*** L. subsp. ***pyraster*** (L.) Ehrh.

***Pyrus spinosa*** Forssk.

***Rhaphiolepis bibas*** (Lour.) Galasso & Banfi, A CAS

***Rosa canina*** L.

***Rosa chinensis*** Jacq. var. ***semperflorens*** (Curtis) Koehne

***Rosa gallica*** L.

***Rosa sempervirens*** L.

***Rubus caesius*** L.

***Rubus ulmifolius*** Schott

***Sorbus domestica*** L.

RHAMNACEAE Juss.

***Paliurus spina-christi*** Mill.

***Rhamnus alaternus*** L.

***Ziziphus jujuba*** Mill., A CAS

ULMACEAE Mirb.

***Ulmus minor*** Mill.

CANNABACEAE Martinov

***Celtis australis*** L. subsp. ***australis***

***Humulus lupulus*** L.

MORACEAE Gaudich.

***Broussonetia papyrifera*** (L.) Vent., A NAT

***Ficus carica*** L.

***Maclura pomifera*** (Raf.) C.K. Schneid., A CAS

***Morus alba*** L., A CAS

URTICACEAE Juss.

***Parietaria judaica*** L.

***Parietaria lusitanica*** L. subsp. ***susitanica***

***Urtica dioica*** L., S

***Urtica membranacea*** Poir.

***Urtica pilulifera*** L.

***Urtica urens*** L., S

FAGACEAE Dumort.

***Castanea sativa*** Mill.

***Quercus cerris*** L.

***Quercus dalechampii*** Ten.

***Quercus frainetto*** Ten.

***Quercus ilex*** L. subsp. ***ilex***

***Quercus pubescens*** Willd. subsp. ***pubescens***

***Quercus robur*** L. subsp. ***robur***

***Quercus suber*** L.

JUGLANDACEAE DC. ex Perleb

***Juglans regia*** L., A CAS

CUCURBITACEAE Juss.

***Bryonia dioica*** Jacq.

***Cucurbita maxima*** Duchense subsp. ***maxima***, A CAS

***Ecballium elaterium*** (L.) A. Rich., S

CELASTRACEAE R.Br.

***Euonymus europaeus*** L.

OXALIDACEAE R.Br.

***Oxalis articulata*** Savigny, A NAT, S

***Oxalis corniculata*** L., S

***Oxalis dillenii*** Jacq., A NAT, S

***Oxalis pes-caprae*** L., A NAT, S

VIOLACEAE Batsch

***Viola alba*** Besser subsp. ***denhardtii*** (Ten.) W.Becker, S

***Viola arvensis*** Murray, S

***Viola reichenbachiana*** Jordan ex Boreau

SALICACEAE Mirb.

***Populus alba*** L. subsp. ***alba***

***Populus nigra*** L.

***Salix alba*** L. subsp. ***alba***

PASSIFLORACEAE Juss. ex Roussel

***Passiflora caerulea*** L., A CAS

LINACEAE DC. ex Perleb

***Linum usitatissimum*** L. subsp. ***angustifolium*** (Huds.) Thell., S

***Linum strictum*** L.

HYPERICACEAE Juss.

***Hypericum perforatum*** L., S

EUPHORBIACEAE Juss.

***Euphorbia characias*** L.

***Euphorbia exigua*** L. subsp. ***exigua***, sS

***Euphorbia falcata*** L. subsp. ***falcata***, sS

***Euphorbia helioscopia*** L. subsp. ***helioscopia***, S

***Euphorbia peplus*** L., S

***Euphorbia platyphyllos*** L.

***Euphorbia prostrata*** Aiton, A NAT, S

***Euphorbia pulcherrima*** Willd. ex Klotzsch, A CAS

***Euphorbia terracina*** L., S

***Mercurialis annua*** L., S

GERANIACEAE Juss.

***Erodium acaule*** (L.) Bech. & Thell.

***Erodium ciconium*** (L.) L’Hér., S

***Erodium cicutarium*** (L.) L’Hér., S

***Erodium malacoides*** (L.) L’Hér. subsp. ***malacoides***, S

***Erodium moschatum*** (L.) L’Hér.

***Geranium columbinum*** L., S

***Geranium dissectum*** L., S

***Geranium molle*** L., S

***Geranium robertianum*** L., S

***Geranium rotundifolium*** L., S

***Geranium sanguineum*** L., S

ONAGRACEAE Juss.

***Epilobium hirsutum*** L.

***Epilobium lanceolatum*** Sebast. & Mauri

***Epilobium parviflorum*** Schreber, S

***Epilobium tetragonum*** L. subsp. ***tournefortii*** (Michalet) H.Lév., S

LYTHRACEAE J.St.-Hil.

***Lythrum hyssopifolia*** L., S

***Lythrum salicaria*** L., S

***Punica granatum*** L., A CAS

MYRTACEAE Juss.

***Eucalyptus camaldulensis*** Dehnh. subsp. ***camaldulensis***, A CAS

ANACARDIACEAE R.Br.

***Pistacia lentiscus*** L.

***Pistacia terebinthus*** L. subsp. ***terebinthus***

SAPINDACEAE Juss.

***Acer campestre*** L.

***Acer monspessulanum*** L. subsp. ***monspessulanum***

***Acer negundo*** L., A NAT

RUTACEAE Juss.

***Ruta chalepensis*** L.

SIMAROUBACEAE DC.

***Ailanthus altissima*** (Mill.) Swingle, A INV

MELIACEAE Juss.

***Melia azedarach*** L., A CAS

MALVACEAE Juss.

***Althaea cannabina*** L.

***Malva multiflora*** (Cav.) Soldano, Banfi & Galasso, S

***Malva nicaeensis*** All., S

***Malva punctata*** (All.) Alef.

***Malva sylvestris*** L., S

RESEDACEAE Martinov

***Reseda alba*** L., S

***Reseda lutea*** L. subsp. ***lutea***, S

***Reseda phyteuma*** L. subsp. ***phyteuma***, S

***Tarenaya spinosa*** (Jacq.) Raf., A CAS

CAPPARACEAE Juss.

***Capparis orientalis*** Veill.

BRASSICACEAE Burnett

***Alliaria petiolata*** (Bieb.) Cavara et Grande, S

***Arabis hirsuta*** (L.) Scop.

***Berteroa obliqua*** (Sm.) DC.

***Brassica oleracea*** L., A CAS

***Bunias erucago*** L., S

***Calepina irregularis*** (Asso) Thell., S

***Capsella bursa-pastoris*** (L.) Medik., S

***Capsella rubella*** Reuter, S

***Cardamine hirsuta*** L., S

***Diplotaxis erucoides*** (L.) DC subsp. **erucoides**, S

***Diplotaxis tenuifolia*** (L.) DC., S

***Draba verna*** L.

***Lepidium graminifolium*** L.

***Lepidium virginicum*** L., A CAS

***Lunaria annua*** L., A CAS

***Lunaria rediviva*** L.

***Microthlaspi perfoliatum*** (L.) F.K.Mey., S

***Nasturtium officinale*** W.T. Aiton

***Neslia paniculata*** (L.) Desv. subsp. ***thracica*** (Velen.) Bornm., cS

***Raphanus raphanistrum*** L. subsp. ***landra*** (Moretti ex DC.) Bonnier & Layens, S

***Rapistrum rugosum*** (L.) All., cS

***Sinapis alba*** subsp. ***alba***, cS

***Sinapis arvensis*** L. subsp. ***arvensis***, cS

***Sisymbrium officinale*** (L.) Scop.

SANTALACEAE R.Br.

***Osyris alba*** L.

PLUMBAGINACEAE Juss.

***Plumbago auriculata*** Lam., A CAS

***Plumbago europaea*** L.

POLYGONACEAE Juss.

***Fallopia baldschuanica*** (Regel) Holub, A NAT

***Fallopia dumetorum*** (L.) J. Holub, S

***Persicaria amphibia*** (L.) Delabre

***Persicaria lapathifolia*** (L.) Delarbre

***Persicaria maculosa*** Gray, S

***Polygonum aviculare*** L., S

***Polygonum rurivagum*** Jord. ex Boreau, S

***Rumex bucephalophorus*** L. subsp. ***bucephalophorus***, S

***Rumex conglomeratus*** Murray, S

***Rumex crispus*** L., S

***Rumex obtusifolius*** L. subsp. ***obtusifolius***, S

***Rumex pulcher*** L. subsp. ***pulcher***, S

CARYOPHYLLACEAE Juss.

***Arenaria leptoclados*** (Reichenb.) Guss., S

***Arenaria serpyllifolia*** L., S

***Cerastium brachypetalum*** Desp. ex Pers., S

***Cerastium glomeratum*** Thuill., S

***Cerastium ligusticum*** Viv., S

***Dianthus armeria*** L. subsp. ***armeria***

***Herniaria glabra*** L., cS

***Herniaria hirsuta*** L. subsp. ***hirsuta***, S

***Petrorhagia saxifraga*** (L.) Link subsp. ***saxifraga***, S

***Petrorhagia dubia*** (Raf.) G.López & Romo

***Polycarpon tetraphyllum*** (L.) subsp. ***diphyllum*** (Cav.) O. Bolòs et Font Quer

***Polycarpon tetraphyllum*** L. subsp. ***tetraphyllum***, S

***Sagina apetala*** Ard. subsp. ***apetala***

***Saponaria officinalis*** L.

***Silene bellidifolia*** Jacq.

***Silene conica*** L., S

***Silene gallica*** L., S

***Silene gallinyi*** Rchb., NC

***Silene italica*** (L.) Pers. subsp. ***italica***, S

***Silene latifolia*** Poir., S

***Silene nocturna*** L., S

***Silene pendula*** L.

***Silene vulgaris*** (Moench) Garcke subsp. ***tenoreana*** (Colla) Soldano & F.Conti, S

***Silene vulgaris*** (Moench.) Garcke subsp. ***vulgaris***, S

***Spergularia rubra*** (L.) J.Presl & C.Presl, S

***Stellaria media*** (L.) Vill. subsp. ***media***, S

***Stellaria neglecta*** Weihe, S

***Stellaria pallida*** (Dumort.) Crép.

***Spergula arvensis*** L., sS

CHENOPODIACEAE Vent.

***Atriplex patula*** L., S

***Beta vulgaris*** L. subsp. ***vulgaris***

***Chenopodiastrum murale*** (L.) S.Fuentes, Uotila & Borsch, S

***Chenopodium opulifolium*** Schrader ex Koch et Ziz, S

***Chenopodium album*** L., S

***Chenopodium vulvaria*** L.

***Dysphania ambrosioides*** (L.) Mosyakin & Clemants, A NAT

***Dysphania multifida*** (L.) Mosyakin & Clemants, A CAS

***Lipandra polysperma*** (L.) S.Fuentes, Uotila & Borsch, S

***Oxybasis urbica*** (L.) S.Fuentes, Uotila & Borsch

AMARANTHACEAE Juss.

***Amranthus albus*** L., A NAT

***Amaranthus blitoides*** S. Watson, A NAT

***Amaranthus blitum*** L. subsp. ***blitum***

***Amaranthus deflexus*** L., A INV, S

***Amaranthus graecizans*** subsp. ***silvestris*** (Vill.) Brenan, S

***Amaranthus hybridus*** L., A INV, S

***Amaranthus retroflexus*** L., A INV, S

***Amaranthus viridis*** L., A INV

***Polycnemum heuffelii*** Láng,, C, NC

PHYTOLACCACEAE R.Br.

***Phytolacca americana*** L., A INV

NYCTAGINACEAE Juss.

***Mirabilis jalapa*** L., A NAT

BASELLACEAE Raf.

***Anredera cordifolia*** (Ten.) Steenis, A NAT

PORTULACACEAE Juss.

***Portulaca oleracea*** L., S

CACTACEAE Juss.

***Opuntia ficus-indica*** (L.) Mill., A NAT

CORNACEAE Bercht. & J.Presl

***Cornus mas*** L.

***Cornus sanguinea*** L. subsp. ***sanguinea***

EBENACEAE Gürke

***Diospyros kaki*** Thunb., A CAS

HYDRANGEACEAE Dumort.

***Hydrangea macrophylla*** (Thunb.) Ser., A NAT

PRIMULACEAE Batsch ex Borkh.

***Cyclamen hederifolium*** Aiton

***Cyclamen repandum*** Sm.

***Lysimachia arvensis*** (L.) U.Manns & Anderb. subsp. ***arvensis***, cS

***Lysimachia foemina*** (Mill.) U.Manns & Anderb., S

ERICACEAE Juss.

***Arbutus unedo*** L.

RUBIACEAE Juss.

***Cruciata laevipes*** Opiz, S

***Galium album*** Mill. (*G. erectum* Syme) subsp. ***album***, S

***Galium aparine*** L., S

***Galium murale*** (L.) All.

***Galium parisiense*** L.

***Rubia peregrina*** L.

***Sherardia arvensis*** L., S

***Theligonum cynocrambe*** L.

***Valantia muralis*** L.

GENTIANACEAE Juss.

***Blackstonia perfoliata*** (L.) Huds. subsp. ***perfoliata***

***Centaurium erythraea*** Rafn subsp. ***erythraea***, S

***Centaurium maritimum*** (L.) Fritsch

***Centaurium tenuiflorum*** (Hoffmanns. et Link) Fritsch subsp. ***acutiflorum*** (Schott) Zeltner, S

APOCYNACEAE Juss.

***Nerium oleander*** L., A CAS

***Trachelospermum jasminoides*** (Lindl.) Lem., A CAS

***Vinca major*** L. subsp. ***major***

***Vinca minor*** L.

CONVOLVULACEAE Juss.

***Convolvulus arvensis*** L., S

***Convolvulus cantabrica*** L.

***Convolvulus sepium*** L.

***Convolvulus silvaticus*** Kit.

***Cuscuta campestris*** Yunck, A NAT

***Cuscuta cesattiana*** Bertol., A CAS

***Ipomoea indica*** (Burm.) Merr., A CAS

SOLANACEAE Juss.

***Datura stramonium*** L., S

***Lycium chinense*** Mill., A NAT

***Solanum chenopodioides*** Lam., A NAT

***Solanum dulcamara*** L.

***Solanum lycopersicum*** L., A NAT

***Solanum nigrum*** L., S

***Solanum pseudocapsicum*** L., A CAS

***Solanum villosum*** Mill., S

BORAGINACEAE Juss.

***Anchusa azurea*** Mill., S

***Anchusa undulata*** L. subsp. ***hybrida*** (Ten.) Bég., S

***Borago officinalis*** L., S

***Buglossoides arvensis*** (L.) I.M.Johnst., cS

***Cynoglossum creticum*** Mill., S

***Echium italicum*** L. subsp. ***italicum***

***Echium parviflorum*** M\ch, S

***Echium plantagineum*** L., S

***Echium vulgare*** L., S

***Myosotis arvensis*** (L.) Hill subsp. ***arvensis***, cS

***Myosotis ramosissima*** Rochel subsp. ***ramosissima***, S

***Symphytum officinale*** L., S

***Symphytum tuberosum*** L. subsp. ***angustifolium*** (A.Kerner) Nyman, S

HELIOTROPIACEAE Schrader

***Heliotropium amplexicaule*** Vahl., A CAS

***Heliotropium europaeum*** L., S

OLEACEAE Hoffmanns. & Link

***Fraxinus angustifolia*** Vahl subsp. ***oxycarpa*** (Willd.) Franco et Rocha Afonso

***Fraxinus ornus*** L. subsp. ***ornus***

***Ligustrum lucidum*** W.T. Aiton, A CAS

***Ligustrum vulgare*** L.

***Phillyrea latifolia*** L.

PLANTAGINACEAE Juss.

***Antirrhinum majus*** L.

***Antirrhinum tortosuom*** Bosc ex Lam, S

***Callitriche stagnalis*** Scop.

***Cymbalaria muralis*** G. Gaertn., B. Mey. et Scherb. subsp. ***muralis***

***Linaria pelisseriana*** (L.) Mill., NC

***Linaria purpurea*** (L.) Mill., S

***Linaria vulgaris*** Mill. subsp. ***vulgaris***, S

***Misopates calycinum*** Rothm.

***Plantago afra*** L. subsp. ***afra***, S

***Plantago lagopus*** L.

***Plantago lanceolata*** L., S

***Plantago major*** L., S

***Veronica anagallis-aquatica*** L., S

***Veronica arvensis*** L., cS

***Veronica cymbalaria*** Bodard, S

***Veronica hederifolia*** L., S

***Veronica persica*** Poir., S, A INV

***Veronica polita*** Fr., cS

SCROPHULARIACEAE Juss.

***Scrophularia auriculata*** L.

***Scrophularia peregrina*** L.

***Verbascum blattaria*** L.

***Verbascum sinuatum*** L., S

***Verbascum thapsus*** L. subsp. ***thapsus***

LAMIACEAE Martinov

***Ballota nigra*** L. subsp. ***meridionalis*** (Bég.) Bég., sS

***Clinopodium menthifolium*** (Host) Merino subsp. ***ascendens*** (Jord.) Govaerts, S

***Clinopodium vulgare*** L., S

***Lamium amplexicaule*** L., S

***Lamium bifidum*** Cirillo subsp. ***bifidum***, S

***Lamium maculatum*** L.

***Lamium purpureum*** L., cS

***Lycopus europaeus*** L.

***Marrubium vulgare*** L.

***Melissa officinalis*** L. subsp. ***altissima*** (Sm) Arcang., S

***Mentha aquatica*** L. subsp. ***aquatica***, S

***Mentha pulegium*** L. subsp. ***pulegium***, S

***Mentha suaveolens*** Ehrh. subsp. ***suaveolens***, S

***Micromeria graeca*** (L.) Benth. ex Rchb.

***Origanum vulgare*** L. subsp. ***vulgare***

***Prunella laciniata*** (L.) L.

***Salvia clandestina*** L.

***Salvia verbenaca*** L., S

***Thymbra capitata*** (L.) Cav.

***Micromeria graeca*** (L.) Benth. ex Rchb.

***Micromeria juliana*** (L.) Benth. ex Rchb.

***Stachys arvensis*** (L.) L., S

***Stachys germanica*** L. subsp. ***germanica***, S, NC

***Stachys ocymastrum*** (L.) Briq., S

***Stachys romana*** (L.) E.H.L.Krause, S

***Stachys sylvatica*** L.

***Teucrium chamaedrys*** L. subsp. ***chamaedrys***

***Teucrium flavum*** L. subsp. ***flavum***

OROBANCHACEAE Vent.

***Bellardia trixago*** (L.) All., S

***Bellardia viscosa*** (L.) Fisch. & C.A.Mey., S

***Odontites vernus*** (Bellardi) Dumort. subsp. ***serotinus*** Corb.

***Orobanche crenata*** Forssk., cS

***Orobanche hederae*** Vaucher ex Duby

***Parentucellia latifolia*** (L.) Caruel, S

***Phelipanche nana*** (Reut.) Soják

VERBENACEAE J.St.-Hil.

***Lantana camara*** L., A CAS

***Verbena officinalis*** L., S

ACANTHACEAE Juss.

***Acanthus mollis*** L. subsp. ***mollis***

***Ruellia simplex*** C. Wright, A CAS

BIGNONIACEAE Juss.

***Campsis radicans*** (L.) Bureau, A NAT

CAMPANULACEAE Juss.

***Campanula erinus*** L., S

***Campanula rapunculus*** L., S

***Jasione montana*** L. subsp. ***montana***

***Legousia speculum-veneris*** (L.) Chaix subsp. ***speculum-veneris***, sS

ASTERACEAE Bercht. & J.Presl

***Anacyclus radiatus*** Loisel. subsp. ***radiatus***, S

***Andryala integrifolia*** L., S

***Anthemis arvensis*** L. subsp. ***arvensis***, cS

***Anthemis cotula*** L., cS

***Arctium minus*** (Hill) Bernh.

***Artemisia arborescens*** (Vaill.) L.

***Artemisia verlotiorum*** Lamotte, S

***Artemisia vulgaris*** L., S

***Bellis annua*** L. subsp. ***annua***

***Bellis perennis*** L., S

***Bellis sylvestris*** Cirillo

***Bidens subalternans*** DC., A CAS

***Calendula arvensis*** (Vill.) L., S

***Carduus micropterus*** (Borbàs) Teyber subsp. ***perspinosus*** (Fiori) Arènes

***Carduus nutans*** L. subsp. ***nutans***, S

***Carduus pycnocephalus*** L. subsp. ***pycnocephalus***, S

***Carlina corymbosa*** L., S

***Carthamus lanatus*** L.

***Centaurea calcitrapa*** L., S

***Centaurea jacea*** L. subsp. ***angustifolia*** (DC.) Gremli, S

***Centaurea napifolia*** L., S

***Centaurea solstitialis*** L. subsp. ***solstitialis***, S

***Chondrilla juncea*** L.

***Cladanthus mixtus*** (L.) Chevall.

***Cota tinctoria*** (L.) J.Gay subsp. ***tinctoria***, S

***Cichorium intybus*** L., S

***Cirsium arvense*** (L.) Scop., S

***Cirsium creticum*** (Lam.) d’Urv. subsp. ***triumfetti*** (Lacaita) Werner

***Coleostephus myconis*** (L.) Cass. ex Rchb.f.

***Cyanus segetum*** L., sS

***Crepis bursifolia*** L.

***Crepis neglecta*** L. subsp. ***neglecta***, S

***Crepis sancta*** (L.) Bornm. subsp. ***nemausensis*** (P.Fourn.) Babc., S, A NAT

***Crepis setosa*** Haller f., S

***Crepis vesicaria*** L., S

***Dittrichia graveolens*** (L.) Greuter

***Dittrichia viscosa*** (L.) Greuter subsp. ***viscosa***

***Erigeron bonariensis*** L., A NAT, S

***Erigeron canadensis*** L., A CAS, S

***Erigeron sumatrensis*** Retz., A INV, S

***Eupatorium cannabinum*** L. subsp. ***cannabinum***, S

***Filago germanica*** (L.) Huds., cS

***Galactites tomentosus*** Moench, S

***Galinsoga parviflora*** Cav., A CAS, S

***Galinsoga quadriradiata*** Ruiz et Pav., A NAT, S

***Glebionis segetum*** (L.) Fourr., S

***Helianthus tuberosus*** L., A CAS, S

***Hypochaeris achyrophorus*** L., S

***Hypochoeris radicata*** L.

***Jacobaea aquatica*** (Hill) G. Gaertn., B. Mey. & Scherb.

***Jacobaea erratica*** (Bertol.) Fourr.

***Lactuca sativa*** L. subsp. ***serriola*** (L.) Galasso, Banfi, Bartolucci & Ardenghi, S

***Leontodon tuberosus*** L.

***Matricaria chamomilla*** L., cS

***Onopordum acanthium*** L. subsp. ***acanthium***

***Onopordum illyricum*** L., S

***Pallenis spinosa*** (L.) Cass. subsp. ***spinosa***, S

***Pentanema squarrosum*** (L.) D.Gut.Larr., Santos- Vicente, Anderb., E.Rico & M.M.Mart.Ort., S

***Petasites hybridus*** (L.) G.Gaertn., B.Mey. & Scherb. subsp. ***hybridus***

***Helminthotheca echioides*** (L.) Holub

***Picris hieracioides*** L. subsp. ***hieracioides***, S

***Picris hieracioides*** L. subsp. ***spinulosa*** (Guss.) Arcang.

***Pulicaria dysenterica*** (L.) Bernh., S

***Reichardia picroides*** (L.) Roth, S

***Rhagadiolus stellatus*** (L.) Gaertn., S

***Scolymus hispanicus*** L., S

***Senecio leucanthemifolius*** Poir., S

***Senecio vulgaris*** L., S

***Silybum marianum*** (L.) Gaertn., S

***Sonchus asper*** (L.) Hill subsp. ***asper***, S

***Sonchus oleraceus*** L., S

***Sonchus tenerrimus*** L.

***Symphyotrichum squamatum*** (Spreng.) G.L.Nesom, A NAT

***Taraxacum*** F.H.Wigg. sect. **Taraxacum**, S

***Tragopogon porrifolius*** L., S

***Tussilago farfara*** L., S

***Tyrimnus leucographus*** (L.) Cass.

***Urospermum dalechampii*** (L.) F.W. Schmidt, S

***Urospermum picroides*** (L.) Scop. ex F.W. Schmidt, S

***Xanthium spinosum*** L., A NAT

**Xanthium italicum** Moretti, S

VIBURNACEAE Rafinesque

***Sambucus ebulus*** L.

***Sambucus nigra*** L.

***Viburnum tinus*** L. subsp. ***tinus***

CAPRIFOLIACEAE Juss.

***Lonicera etrusca*** Santi

***Lonicera japonica*** Thunb., A NAT

DIPSACACEAE Juss.

***Dipsacus fullonum*** L.

***Knautia arvensis*** (L.) Coult., S

***Knautia collina*** Jord.

***Knautia integrifolia*** (L.) Bertol. subsp. ***integrifolia***, S

***Sixalix atropurpurea*** (L.) Greuter et Burdet

PITTOSPORACEAE R.Br.

***Pittosporum tobira*** (Thunb.) W.T. Aiton, A CAS

VALERIANACEAE Batsch

***Centranthus ruber*** (L.) DC. subsp. ***ruber***

***Valerianella eriocarpa*** Desv., cS

ARALIACEAE Juss.

***Hedera helix*** L. subsp. ***helix***

APIACEAE Lindl.

***Ammi majus*** L., S

***Ammoides pusilla*** (Brot.) Breistr.

***Anethum piperitum*** Ucria, S

***Angelica sylvestris*** L.

***Anthriscus sylvestris*** (L.) Hoffm.

***Chaerophyllum temulum*** L., S

***Conium maculatum*** L., S

***Daucus carota*** L. subsp. ***carota***, S

***Daucus carota*** L. subsp. ***maximus*** (Desf.) Ball, S

***Eryngium campestre*** L., S

***Ferula communis*** L. subsp. ***communis***, S

***Ferula glauca*** L.

***Helosciadium nodiflorum*** (L.) W.D.J.Koch subsp. ***nodiflorum***

***Oenanthe pimpinelloides*** L., S

***Oenanthe silaifolia*** M.Bieb.

***Opopanax chironium*** (L.) W.D.J.Koch

***Smyrnium olusatrum*** L.

***Tordylium apulum*** L., S

***Tordylium maximum*** L., S

***Torilis arvensis*** (Hudson) Link subsp. ***arvensis***, S

***Torilis japonica*** (Houtt.) DC., S

## Appendix B

Selected specimens collected during the field surveys. The list (alphabetical order of the scientific names) refers to floristic novelties, notable species, no longer recorded taxa, and taxa having *loci classici* and/or nomenclatural types collected in Appia Antica Regional Park (see Section “4.4. Floristic Notes”).

***Aloe maculata* All. subsp. *maculata***

Italy, Latium, Roma, Parco Regionale dell’Appia Antica, Valle della Caffarella, incolto, 27 m a.s.l., 22 April 2012, *G. Nicolella s.n.* (RO); *ibidem*, 23 April 2017, *D. Iamonico* (RO).

***Amaranthus hypochondriacus* L.**

Lazio, Rome Province, Rome city, Appia Antica Regional Park, locality Acquedotti, channels, 56 m a.s.l., 6 October 2016, *D. Iamonico s.n.* (RO!); ibidem, 18 October 2020 (RO!).

***Anredera cordifolia* (Ten.) Steenis**

Italy, Latium, Rome, Appia Antica Regional Park, Acquedotti locality, on *Rubus ulmifolium* dominated community, 41°51′04′′ N, 12°33′12′′ E, 6 October 2019, *D. Iamonico s.n.* (RO).

***Biarum tenuifolium* (L.) Schott. subsp. *tenuifolium*.**

Italy. Lazio region, Rome Province, Rome city, Appia Antica Regional Park, shrubs vegetation dominated by *Paliurus spina-christi* Mill., 40–42 m a.s.l., 20 August 2015, *D. Iamonico s.n.* (HFLA No. 4861!).

***Bidens subalternans* DC.**

Italy, Latium, Rome, Appia Antica Regional Park, Acquedotti locality, on a little bridge of channel Acqua Mariana, 41°51′02′′ N, 12°33′21′′ E, 23 November 2020, *D. Iamonico s.n.* (RO).

***Campsis radicans* (L.) Bureau**

Roma, P.R. dell’Appia Antica, Valle della caffarella, marrana, 23 April 2011, *D. Iamonico s.n.* (RO!); ibidem, 1 September 2013 (RO!); Acquedotti locality, 10 August 2021, *D. Iamonico s.n.* (RO!).

***Canna indica* L.**

Italy, Latium, Rome, Appia Antica Regional Park, Acquedotti locality, banks of channel Acqua Mariana, 15 June 2018, *D. Iamonico s.n.* (RO).

***Catabrosa aquatica* (L.) P.Beauv.**

Italy, Latium, Roma, Via Appia Nuova, lungo fossetto tra Ciampino e S. Maria delle Mole. Luoghi aquitrinosi, 20 April 1944, *G. Montelucci s.n.* (RO-Herbarium Montelucci, sub *Aira aquatica* L.).

***Chlorophytum comosum* (Thumbs.) Jacques**

Italy, Latium, Rome, Appia Antica Regional Park, Acquedotti locality, banks of channel Acqua Mariana, 15 May 2019, *D. Iamonico s.n.* (RO).

***Colocasia esculenta* (L.) Schott**

Lazio region, Rome, Appia Antica Regional Park, locality Acquedotti, channels, 58 m a.s.l., 8 August 2015, *D. Iamonico s.n.* (HFLA!); ibidem, 19 m a.s.l., 9 May 2020 (RO!).

***Cyperus alternifolius* L. subsp. *flabelliforme* Kük.**

Lazio region, Rome, Appia Antica Regional Park, Caffarella valley, channels, 25 May 2017 *D. Iamonico s.n.* (RO!); locality Acquedotti, channels, 56 m a.s.l., 15 May 2022, *D. Iamonico s.n.* (RO!).

***Denisophytum**bessac* (Choiv.) E.Gagnon & G.P.Lewis**

Italy, Latium, Rome, Appia Antica Regional Park, via Appia Pignatelli, shrubs, 24 April 2017, *D. Iamonico s.n.* (RO); *ibidem*, 25 May 2022, *D. Iamonico s.n.* (RO).

***Diospyrus kaki* L.**

Italy, Latium, Rome, Appia Antica Regional Park, Acquedotti locality, banks of channel Acqua Mariana, 5 October 2020, *D. Iamonico s.n.* (RO).

***Ehrharta erecta* Lam.**

Italy, Latium, Roma, Valle della Ninfa Egeria, 17 March 1876, *G. G.. Cuboni s.n.* (RO-Herbarium Romano no. 51234).

***Epilobium lanceolatum* Sebast. & Mauri**

Italy, Lazio, Roma nei prati della Caffarella, 3 June 1812, *F. A. Sebastiani* (RO-Herbarium Romano, n. 19436); Lazio, Via delle Vigne presso Albano, June 1853, *E. Rolli* (RO-Herbarium Romano, n. 19443).

***Euphorbia pulcherrima* Willd. ex Klotzsch**

Italy, Latium, Rome, Appia Antica Regional Park, Acquedotti locality, on riverbed of channel Acqua Mariana, 3 June 2022, *D. Iamonico s.n.* (RO).

***Heliotropium amplexicaule* Vahl.**

Italy, Latium, Rome, April 1928, *s.c.* (RO-HG, no. 32109); Rome, Appia Antica Regional Park, along on central reservation of the Appia Nuova street, 3 June 2022, *D. Iamonico s.n.* (RO).

***Hydrangea macrophylla* (Thunb.) Ser.**

Italy, Latium, Rome, Appia Antica Regional Park, Acquedotti locality, on riverbed of channel Acqua Mariana, 7 August 2016, *D. Iamonico s.n.* (RO); *ibidem*, 29 December 2020, *D. Iamonico s.n.* (RO).

***Kalanchoe daigremontiana* Raym.**

Lazio region, Rome, Appia Antica Regional Park, Caffarella valley, *Arando donax* L. dominated community, 20 June 2020, *D. Iamonico s.n.* (RO!).

***Lemna minuta* Kunth**

Roma, Parco Regionale dell’Appia Antica, loc. Vaccareccia Caffarella, channel and artificial reservoir, 23 m a.s.l., 7 September 2007, *Iamonico s.n.* (RO!, HFLA!)

***Linaria pelisseriana* (L.) Mill.**

Italy, Latium, Roma, Via Ardeatina, 15 May 1892, *L. Salomonsohn s.n.* (RO-Herbarium Romano no. 34429).

***Lupinus albus* L. subsp. *graecus* (Boiss. et Spruner) Franco & Pinto da Silva**

Italy, Latium, Rome, Appia Antica Regional Park, Caffarella valley, meadows, 25 April 2021, *D. Iamonico s.n.* (RO).

***Melia azedarach* L.**

Lazio region, Rome, Appia Antica Regional Park, Caffarella valley, shrubs, 25 May 2018, 22 m a.s.l., *D. Iamonico s.n.* (RO!); locality Acquedotti, anthropogenic meadows, 57 m a.s.l., 2 July 2022, *D. Iamonico s.n.* (RO!).

***Papaver somniferum* L.**

Lazio region, Rome, Appia Antica Regional Park, Caffarella valley, meadows, 7 May 2016, *D. Iamonico s.n.* (RO!).

***Parapholis cylindrica* (Willd.) Romero Zarco**

Italy, Latium, Roma, Alla Caffarella, s.d. (XIX century), *s.c. s.n.* (RO-Herbarium Romano no. 55564, sub *Rottboellia subulata* Savi).

***Passiflora caerulea* L.**

Italy, Latium, Rome, Appia Antica Regional Park, Claudio’s aqueduct (Porta Furba), 7 June 2022, *D. Iamonico s.n.* (RO).

***Plumbago auriculata* Lam.**

Italy, Latium, Rome, Appia Antica Regional Park, Caffarella valley, *Rubus ulmifolius* dominated community, 20 June 2020, *D. Iamonico s.n.* (RO).

***Polycnemum heuffelii* Láng**

Italy, Latium, Roma, lungo la via Appia Pignatelli, July 1980, *B. Anzalone s.n.* (RO-Herbarium Romano no. 5993).

***Punica granatum* L.**

Italy, Latium, Rome, Appia Antica Regional Park, Caffarella valley, channels, 23 April 2011, *D. Iamonico s.n.* (RO); *ibidem*, 27 June 2012, *D. Iamonico s.n.* (RO); *ibidem*, 01 September 2013, *D. Iamonico s.n.* (RO), *ibidem*, 29 June 2022, *D. Iamonico s.n.* (RO).

***Rosa chinensis* Jacq. var. *semperflorens* (Curtis) Koehne**

Italy, Latium, Rome, Appia Antica Regional Park, Caffarella valley, shrub community with *Rubus ulmifolius* Schott and *Parthenocissus quinquefolia* Planch, 23 April 2017, *D. Iamonico s.n.* (RO).

***Ruellia simplex* C.Wright**

Italy, Latium, Rome, Appia Antica Regional Park, Acquedotti locality, banks of channel Acqua Mariana, 3 June 2022, *D. Iamonico s.n.* (RO).

***Sagina apetala***

Italy, Lazio region, Rome city, Appia Antica Region Park, Acquedotti sector, among cobblestones (“basolato”) of the ancient Roman road “Latina”, 19 May 2020, *Iamonico s.n.* (RO).

***Silene gallinyi* Rchb.**

Italy, Latium, Roma, alla Caffarella (presso catacombe di Pretestato), 6 August 1966, *A. Cacciato s.n.* (RO-Herbarium Anzalone no. 13276), sub *Silene trinervia* Sebast. & Mauri).

***Stachys germanica* L. subsp. *germanica***

Italy, Latium, Roma, Caffarella, May 1829, *P. Sanguinetti s.n.* (RO-Herbarium Romano no. 38523); incolti aridi della Via Appia Antica pr. La Torre di Cecilia Metella, 3 June 1922, *G. Lusina s.n.* (RO-Herbarium Romano no. 38548); *ibidem* (RO-Herbarium Romano no. 38549); *ibidem* (RO-Herbarium Romano no. 38550); *ibidem* (RO-Herbarium Romano no. 38551).

***Tarenaya spinosa* (Jacq.) Raf.**

Italy, Latium, Roma, macerie a via Appia Pignatelli, 6 September 1966, *B. Anzalone s.n.* (RO-Herbarium Romano s.n., sub *Cleome pungens* Willd); Roma, in via Appia Pignatelli, 12 August 1966, *De Persio s.n.* (RO-Herbarium Romano s.n., sub *Cleome pungens* Willd); Roma, sull’Appia Pignatelli, 14 Semptember 1967, *A. Cacciato s.n.* (RO-Herbarium Romano s.n., sub *Cleome pungens* Willd).

***Trachelospermum jasminoides* (Lindl.) Lem.**

Italy, Latium, Rome, Appia Antica Regional Park, Acquedotti locality, on cliff of channel Acqua Mariana, 15 May 2019, *D. Iamonico s.n.* (RO).

***Typha latifolia* L.**

Italy, Lazio region, Rome administrative province, Appia Antica Regional Park, Caffarella valley, wetlands, 20 m a.s.l., 14 October 2020, *D. Iamonico s.n.* (RO).

***Zantedeschia aetiopica* (L.) Spreng.**

Italy, Latium, Rome, Appia Antica Regional Park, Acquedotti locality, banks of channel Acqua Mariana, 16 May 2019, *D. Iamonico s.n.* (RO).
